# Transcriptome Analysis of Adipose Tissues from Five Sheep Breeds Reveals Key Genes Involved in Fat Deposition

**DOI:** 10.3390/genes17010093

**Published:** 2026-01-17

**Authors:** Yi Yu, Sirui Liu, Ji Yang, Songsong Xu

**Affiliations:** Frontiers Science Center for Molecular Design Breeding (MOE), State Key Laboratory of Animal Biotech Breeding, College of Animal Science and Technology, China Agricultural University, Beijing 100193, China; yuyi15179315544@cau.edu.cn (Y.Y.); cauliusirui@163.com (S.L.); yangji_omics@cau.edu.cn (J.Y.)

**Keywords:** sheep, transcriptome, lipid metabolism, tail types

## Abstract

Background: Sheep (*Ovis aries*) exhibit significant diversity in adipose tissue deposition, which influences meat quality, environmental adaptation, and economic value. Tail fat, in particular, varies widely among breeds, yet the transcriptomic basis of this variation remains incompletely understood. This study aims to systematically compare the transcriptional profiles of five adipose depots across five sheep breeds to identify molecular mechanisms underlying fat deposition and tail phenotype divergence. Methods: We analyzed 250 publicly available RNA-seq samples from five adipose tissues (caul, subcutaneous, perirenal, intermuscular, and tail fat) of five sheep breeds (Altay, Tibetan, Merino, Wadi, Small-tailed Han). Data were processed using FastQC, STAR, and featureCounts. Differential expression analysis was performed with DESeq2, followed by GO and KEGG enrichment analyses. Breeds were grouped into three tail phenotypes: fat-tailed, short fat-tailed, and thin-tailed. Cross-tissue and phenotype-specific pathway analyses were conducted to identify key regulatory genes. Results: Transcriptional divergence was most pronounced in subcutaneous and intermuscular fat, while tail fat exhibited both conserved and phenotype-specific pathways. Fat-tailed breeds showed enrichment in mitochondrial oxidative phosphorylation and lipid biosynthesis genes (*TAFAZZIN*, *GPAM*, *COQ* family). Short fat-tailed breeds were characterized by extracellular matrix remodeling genes (*MMP9*, *MMP12*, *MMP19*). Thin-tailed sheep lacked these pro-lipogenic and structural remodeling pathways. A dual-axis model of tail fat development is proposed to explain phenotypic diversity. Conclusions: This study reveals that distinct molecular mechanisms underpin tail fat phenotypes in sheep: fat-tailed breeds prioritize metabolic efficiency, short fat-tailed breeds rely on ECM remodeling, and thin-tailed breeds lack these enhancements. The identified candidate genes may serve as potential targets for molecular breeding strategies aimed at optimizing fat deposition and adaptive traits in sheep.

## 1. Introduction

Sheep (*Ovis aries*) represent one of the earliest domesticated livestock species, providing meat, milk, and wool for human needs [1]. Following domestication, they exhibit pronounced phenotypic diversity in body conformation and productivity among breeds in different ecological and production system [2]. In particular, the amount and distribution of adipose tissues have been the targets of breeding and selection [3].

Different sheep breeds have different fattening efficiency, which affects meat quality, reproductive performance, and environmental adaptability [4]. In sheep, adipose tissues include subcutaneous, perirenal, caul, intermuscular and tail fat [5], and they differ in cellular composition, endocrine activity, and metabolic roles [6], Thus, these various adipose tissues provide a foundational resources for dissecting the different molecular mechanisms underlying fat deposition [7]. Different from other livestock and model animals, tail fat in sheep represents a specialized fat deposition with adaptive and economic importance [8]. Sheep have been categorized into thin-tailed, short fat-tailed, and fat-rumped types based on the extent and distribution of fat deposition [9]. The diversity of tail phenotype is widely considered to be the results of combined natural and artificial selection, reflecting the interplay among energy metabolism, environmental tolerance, and human preference [10]. However, the molecular basis of tail fat deposition in sheep remains insufficiently understood, and only a limited number of studies have specifically revealed the transcriptomic profiles in different types of tail fats [11]. Despite these advances, a systematic characterization for the transcriptomic difference among breeds of different tail types and with different fat deposition efficiency remains scarce, especially with adequate biological replicates to ensure robust statistical inference [12]. Therefore, further exploration on the molecular mechanisms underlying tail fat deposition in sheep of different tail types will enhance the understanding of lipid metabolism biology and provide molecular tools for future sheep breeding and production.

To fill this gap, we conducted a large-scale transcriptomic analysis across five adipose depots (caul, subcutaneous, perirenal, tail, intermuscular fat) in five sheep breeds (Altay sheep, Tibetan sheep, Merino sheep, Wadi sheep, Small-tailed Han sheep), consisting of 250 RNA-seq samples, with 10 biological replicates per depot per breed, enabling a comprehensive examination of breed- and depot-specific transcriptional regulation. Differential expression gene and GO/KEGG enrichment analyses were implemented to explore shared and unique mechanisms associated with fat deposition. We highlight candidate genes and transcription factors (TFs) essential for the tail fat metabolism and tail-type divergence among breeds. Our results provide insights into genetic mechanisms of fat metabolism in different adipose tissues and lay an important foundation for sheep breeding in future.

## 2. Materials and Methods

### 2.1. Adipose Tissue Collection

A total of 13,942 RNA-seq of multiple tissues of *O*. *aries* were retrieved from the NCBI SRA database (https://www.ncbi.nlm.nih.gov/sra, accessed on 15 May 2025). The samples with complete information on sex, breed, and other relevant attributes over the past 10 years have been screened. The selection of these five breeds was based on their distinct phenotypes in adipose deposition and tail types. We only selected Altay sheep, Small-tailed Han Sheep, Tibetan sheep, Wadi sheep and Merino sheep, with all sheep being sexually mature females, and the tissues of “caul fat, tail fat, intermuscular fat, subcutaneous fat and perirenal fat”. The selection of these five breeds was based on their distinct phenotypes in adipose deposition and tail types. For each breed and each tissue type, we filtered those with less than 10 replicate samples. We obtained a final dataset of 250 transcriptomic samples. The corresponding metadata for each accession, including individual sheep information such as age, dietary conditions, and original sample identifiers, were included in Table S4.

### 2.2. Quality Control and Mapping of Reads

After we obtained the FASTQ data, the reads quality was assessed using FastQC v0.12.1 (Babraham Bioinformatics, Cambridge, UK) [13]. Metrics including read counts, percent rRNA, duplication levels, 5′–3′ bias, insert size distribution, and read length distribution were evaluated, and results were aggregated using MultiQC (European Molecular Biology Laboratory, Heidelberg, Germany) for comprehensive visualization and outlier detection. All samples passed our predefined QC thresholds and none were excluded. A detailed, interactive MultiQC summary report will be made publicly available as a persistently accessible web link upon publication and will be listed as Online Supplementary Material. The reads were next mapped to the sheep reference genome Oar_rambouillet_v2.0 (accessed from the National Center for Biotechnology Information Assembly database under accession GCA_016772045.1) by the program STAR v2.7.11b (Cold Spring Harbor Laboratory, Laurel Hollow, NY, USA) [14] with the settings (-quantMode TranscriptomeSAM, -outSAMtype BAM SortedByCoordinate, -outSAMunmapped Within, -outFilterMismatchNmax 3, -outFilterMultimapNmax 10). Properly paired and uniquely mapped reads were extracted using SAMtools v1.21 (Wellcome Sanger Institute, Hinxton, UK) [15] with the command (view -f 2). Gene counts were generated by the featureCounts program from the Subread package suite v2.0.8 (Walter and Eliza Hall Institute of Medical Research, Melbourne, Australia) [16].

### 2.3. Differential Expression Analysis and Functional Annotation

To improve accuracy, genes with very low expression were filtered out; specifical-ly, we retained only genes that had counts ≥5 in at least 20% of the samples within each adipose depot comparison group. To gain a global view on the differences in gene expression across the five adipose tissues of these breeds, pairwise comparisons were conducted within each of the five adipose tissues. For example, in tail fat, a comparison was made between Altay sheep and Tibetan sheep.

Analysis of differentially expressed genes (DEGs) was performed by R package DESeq2 v1.46.0 [17]. Reads were aligned to the *O*. *aries* reference genome assembly Oar_rambouillet_v2.0 using STAR v2.7.11b [14], with the corresponding annotation GTF file from Ensembl (Ensembl release 113, European Molecular Biology Laboratory’s European Bioinformatics Institute, Hinxton, UK). And the false discovery rate (FDR) of each gene in a pair-wise comparison was determined using the Benjamini–Hochberg method. Cutoffs for differential gene expression were selected as FDR < 0.05 and log_2_fold change (log_2_|FC|) > 1. To detect biological functions of DEGs, R package clusterProfiler v4.14.6 [18] was used to conduct the GO functional enrichment analysis and KEGG pathway enrichment analysis. Throughout the analysis, Ensembl gene IDs were used. The clusterProfiler package performed automatic identifier mapping between Ensembl IDs and the annotations required for GO and KEGG analyses using the appropriate OrgDb. The *p*-value of <0.05 (calculated using Fisher’s exact test) was set as the cutoff criteria for the GO and KEGG pathway functional enrichment analysis. All enrichment analyses were performed using the clusterProfiler R package. Terms and pathways with an adjusted *p*-value (Benjamini–Hochberg method) less than 0.05 were considered to be significantly enriched. To minimize inconsistencies in pathway naming across databases and software outputs, all GO/KEGG term names were standardized by converting to lowercase, removing species annotations in parentheses, and trimming redundant spaces. For cross-tissue comparisons, significant GO and KEGG pathways for each depot were merged into a union set. A binary presence–absence matrix was then constructed (rows representing pathways and columns representing breeds), where entries were coded as 0/1 to indicate whether a given pathway reached the significance threshold in the breed. The R package ComplexUpset v1.3.3 was employed to visualize the overlap and divergence of pathways among breeds.

To address potential batch effects present in public RNA-seq data, we implemented the following steps to control and correct for their influence: First, we documented the study/batch source of each sample in Supplementary Table S4. Second, in differential expression analysis within each adipose depot, we incorporated “study/batch” as a covariate in the DESeq2 design formula. Finally, to further verify the robustness of our findings, we performed batch correction on the raw count matrix using the ComBat-seq method (based on a negative binomial model) and repeated the differential expression analysis. The core breed-specific expression patterns and enriched pathways remained consistent before and after correction, indicating that our main conclusions were not substantially confounded by batch effects.

**Figure 1:**
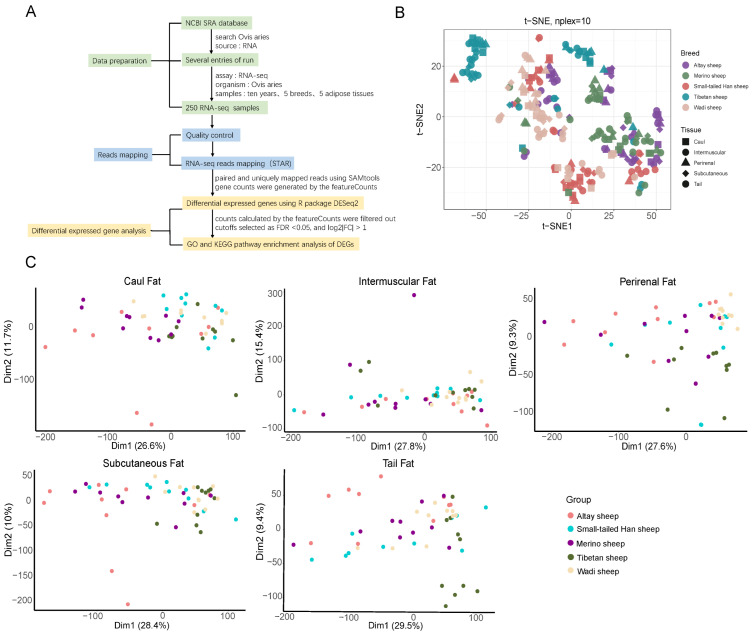
Transcriptome analysis pipeline and tissue clustering. (**A**) Schematic representation of the bioinformatics pipeline employed for transcriptome analysis across sheep adipose depots. (**B**) t-distributed stochastic neighbor embedding (t-SNE) clustering of 250 RNA-Seq samples. (**C**) Principal component analysis performed on the five adipose tissues shows tissue-specific clustering and separation of samples from five sheep breeds.

For dimensionality reduction analyses, including t-distributed stochastic neighbor embedding (t-SNE) and principal component analysis (PCA), gene expression counts were normalized and variance-stabilized using the Variance Stabilizing Transformation (VST) implemented in DESeq2. To account for potential batch effects arising from multiple public sequencing studies, the “study/batch” variable was included as a covariate in the DESeq2 model design, and VST-transformed values were derived from this batch-aware model. The resulting PCA plot (Figure 1C) was regenerated using these adjusted values, and the percentage of variance explained by each principal component is reported in the figure legend.

### 2.4. Phenotype Classification and Candidate Gene Identification

In the analysis of tail fat, the five representative breeds were further classified into three phenotypic categories: fat-rumped (Altay sheep) [19], short fat-tailed (Small-tailed Han sheep and Wadi sheep) [20], and thin-tailed (Merino sheep and Tibetan sheep) [21]. We then compared the distribution of shared and phenotype-specific pathways and categorized the enriched pathways into functional themes, including lipid metabolism, fatty acid oxidation, immune and inflammatory signaling, and extracellular matrix remodeling. Additionally, we identified core genes that were recurrent across multiple enriched pathways. Candidate genes were defined as those enriched in two or more pathways and functionally associated with lipid biosynthesis, fatty acid oxidation, or ECM remodeling. The final results were visualized using bubble plots and dot plots.

### 2.5. Statistical Analysis

All statistical analyses were performed using R (v4.3.1). Principal component analysis (PCA) was applied to visualize global transcriptional differences among breeds and tissues based on normalized gene expression counts. For differential expression analysis, DESeq2 was used with the Benjamini–Hochberg procedure to control the false discovery rate (FDR). Functional enrichment analysis of Gene Ontology (GO) and KEGG pathways was conducted using Fisher’s exact test, with an adjusted *p*-value < 0.05 considered significant. For comparisons of gene expression levels across multiple breeds, one-way ANOVA test was used to assess statistical significance among groups. All plots were generated using ggplot2 (v3.5.0) and related visualization packages. t-SNE analysis was performed using the Rtsne package with default parameters and no fixed random seed was set. No subsampling of the data was in-volved in this analysis. The full statistical details for every DEG in all pairwise comparisons—including gene name, log_2_ fold change, raw *p*-value, and adjusted *p*-value—are available in Table S6.

## 3. Results

### 3.1. RNA-Seq Data and Processing

We conducted 250 publicly available RNA-seq samples from the NCBI SRA database, generating approximately 6 billion reads. Table S1 presents the basic statistics for the RNA sequencing of the five adipose tissues across five breeds (i.e., Altay sheep, Small-tailed Han Sheep, Tibetan sheep, Wadi sheep and Merino sheep).

Figure 1A outlines the RNA-seq analysis pipeline, from data acquisition and quality control to read alignment (STAR), gene quantification (featureCounts), differential expression analysis (DESeq2), and functional enrichment of GOs. The unique mapping rate across all samples ranged from 80.15% to 95.15% when aligned with the sheep reference genome, with all values exceeding the typical threshold of 80%. Therefore, theses high-quality reads ensure the reliability of subsequent analyses and results.

To assess the impact of batch effects on transcriptomic patterns, we first performed batch correction on the raw count matrix using ComBat-seq and repeated the principal component analysis (PCA) and differential expression analysis. The corrected PCA results were consistent with the original analysis, with samples still clustering clearly by adipose tissue and breed, indicating that tissue and breed were the primary drivers of transcriptional variation. Furthermore, the numbers of differentially expressed genes (DEGs) and the patterns of enriched pathways from the batch-corrected data were highly concordant with the uncorrected analysis. The expression trends and significance of core candidate genes were not substantially altered. These results demonstrate that the breed- and tissue-specific transcriptional patterns identified in this study are robust despite potential batch confounding.

### 3.2. Differentially Expressed Gene Analysis

**Figure 2:**
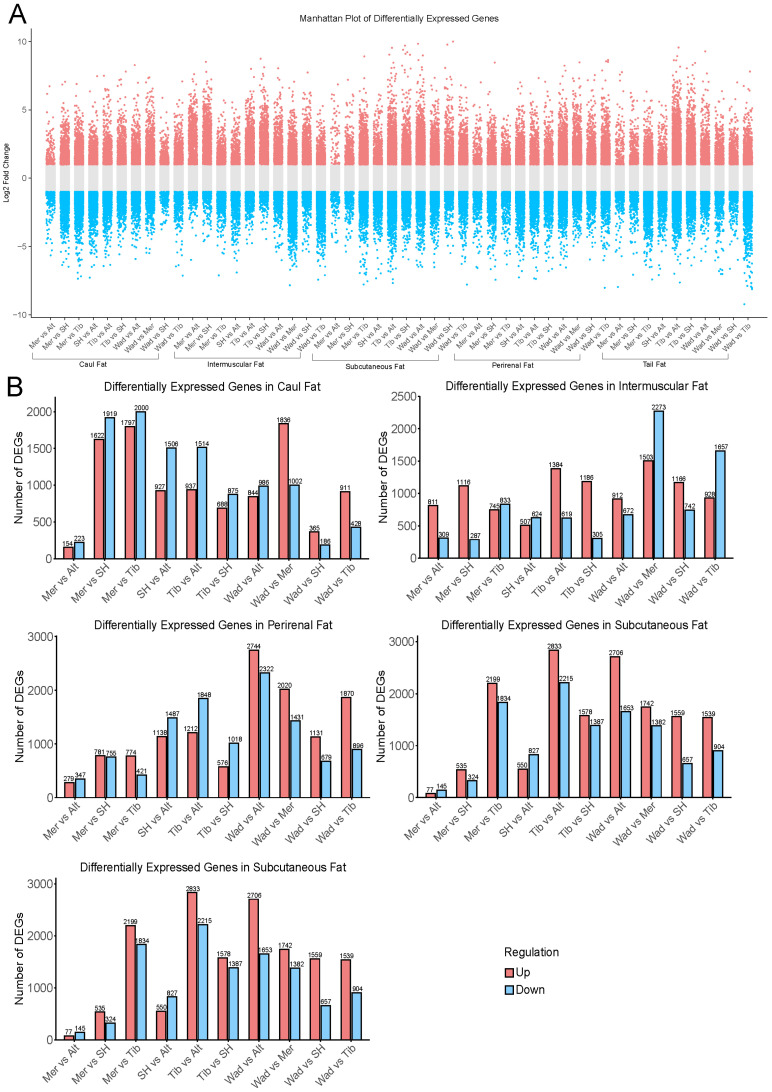
Differential gene expression across breeds and tissues. (**A**) Overview of up and down regulated DEGs across all pairwise comparisons. (**B**) Counts of DEGs identified from pairwise breed comparisons within each of the five adipose tissues; pink represents upregulation, blue represents downregulation.

Following RNA-seq preprocessing and rigorous filtering, we obtained a finalized gene expression matrix comprising 19,440 genes. These genes were considered expressed and used for further analysis. To characterize the global expression patterns of all tissues, we first applied t-distributed stochastic neighbor embedding (t-SNE) analysis based on the gene expression profiles of samples. The resultant sample clustering recapitulated the different tissues and different breeds (Figure 1B). Next, principal component analyses (PCA) were conducted individually for each adipose tissue type, revealing pronounced breed-specific differences across the different fat depots (Figure 1C). Subsequent analyses focused on five adipose depots (i.e., caul fat, tail fat, intermuscular fat, subcutaneous fat, and perirenal fat), where pairwise comparisons were conducted across all the combinations of five sheep breeds. This comparison generated 10 sets of DEGs per adipose depot. Figure 2A presents the comprehensive profile of DEGs, both up and downregulated, across all pairwise comparisons. Considerable variation exists in the number of DEGs for each adipose depot across different breed comparisons, highlighting distinct gene expression pattern in various adipose tissues among the five sheep breeds.

Figure 2B shows the number of DEGs identified in pairwise breed comparisons across five adipose tissues. A closer examination of individual adipose tissues revealed distinct patterns of breed-related transcriptional divergence. In the caul fat, the number of DEGs identified in pairwise breed comparisons was relatively modest compared to other adipose tissues. For example, the highest DEG count was observed in the comparison between Merino and Tibetan sheep, with 3797 DEGs (1797 upregulated, 2000 downregulated). Similarly, the comparisons of Small-tailed Han sheep vs. Altay sheep and Merino vs. Small-tailed Han sheep yielded 2433 and 3541 DEGs, respectively. However, several comparisons, such as Wadi sheep vs. Small-tailed Han sheep and Merino sheep vs. Altay sheep, showed very few DEGs, suggesting minimal transcriptional divergence in caul fat between those breeds. Overall, these results indicate that caul fat exhibits moderate breed-dependent expression differences, with notable divergence primarily involved with Altay sheep. Taken together, these findings highlight that the extent of differential gene expression is strongly influenced by both adipose tissue type and breed identity.

To ensure the statistical rigor of our differential expression analysis, we systematically evaluated the 10 pairwise comparisons within each adipose depot. Volcano plots visually illustrate the distribution of up- and down-regulated genes along with their significance (Figures S1–S5), while MA plots depict the relationship between fold change and mean expression (Figures S6–S10). *p*-value histograms reveal a clear peak near zero in most comparisons, consistent with expected distributions for differential expression analysis (Figures S11–S15). Detailed statistical summaries for each comparison, including the total number of genes tested and significant DEG counts, are provided in Table S5. Together, these results confirm the reliability of our differential expression analysis under appropriate multiple testing control.

### 3.3. Pathway Enrichment Analysis of DEGs

GO enrichment analysis revealed that DEGs identified in each adipose tissue were significantly enriched in multiple biological processes (BPs), molecular functions (MFs), and cellular components (CCs) about fat metabolism. Notably, terms related to lipid biosynthetic process, fatty acid metabolic process, and organic acid metabolic process were recurrently enriched across tissues, albeit with tissue-specific patterns.

Further analysis of the specific enrichment results for each adipose tissue revealed that in subcutaneous fat, DEGs were predominantly associated with fatty acid metabolic process, organic acid metabolic process, and lipid biosynthetic process (GO:0006631—fatty acid metabolic process, GO:0008610—lipid biosynthetic process). Similarly, KEGG pathway analysis indicated strong enrichment in pathways such as fatty acid metabolism, fatty acid biosynthesis, and fatty acid elongation (oas01212—fatty acid metabolism). In contrast, caul fat did not show as many enriched terms. The more prominent GO terms in caul fat included regulation of immune system process, activation of immune response, and immune response-activating signaling pathway, suggesting a role in immune regulation. KEGG pathways such as intestinal immune network for IgA production and ribosome were more prominently enriched in these tissues. In addition, breed-level comparison revealed notable differences in enriched pathways. For instance, DEGs in Wadi sheep and Merino were frequently enriched in immune regulation. Meanwhile, comparisons involving Altay consistently showed strong enrichment for lipid metabolism and adipogenesis-related terms, being consistent with its adaptation to cold climates and fat deposition. In summary, the enrichment results revealed shared biological processes across different adipose tissues, such as fatty acid metabolic processes and lipid biosynthetic processes, among others. However, each tissue also exhibited unique enrichment signatures, reflecting its functional heterogeneity. For instance, terms related to immune system processes were strongly enriched in intermuscular fat. Full lists of enriched terms, including enrichment scores and gene counts, are provided in Table S2.

**Figure 3:**
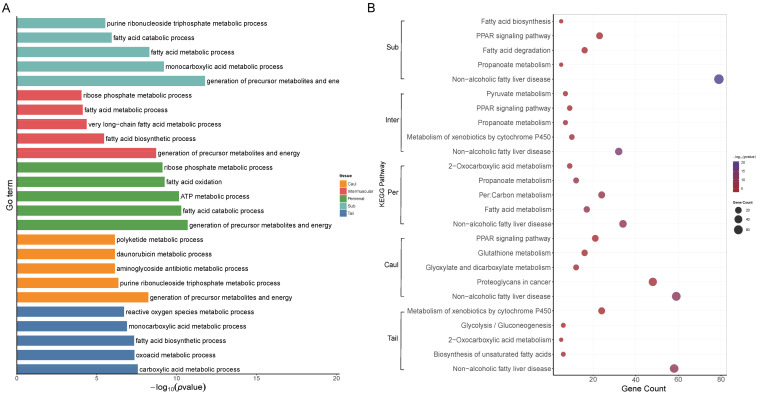
Functional and metabolic pathway enrichment in adipose tissues. (**A**) The significantly enriched GO enrichment process terms of five adipose tissues. (**B**) Enrichment results of key KEGG metabolic pathways for each tissue.

The top five enriched GO terms in each category of DEG sets were visualized as bar plots. For each of the five adipose tissues, we selected pathways related to lipid metabolism from the ten comparison groups as representative sets, displaying only the five most significant results from each relevant subset. Key biological process (BP) terms revealed that the pathways involved in each adipose tissue were consistent with our previous findings (Figure 3A). Further, perirenal adipose tissue was also associated with pathways such as ATP metabolic process. For the KEGG enrichment results, we also selected comparisons from each adipose tissue (Figure 3B). The top five significantly enriched pathways were extracted from each selected comparison. These pathways included those related to lipid metabolism, such as oxidative phosphorylation and carbon metabolism, as well as the PPAR signaling pathway. Furthermore, several disease-associated pathways were observed, including non-alcoholic fatty liver disease.

**Figure 4:**
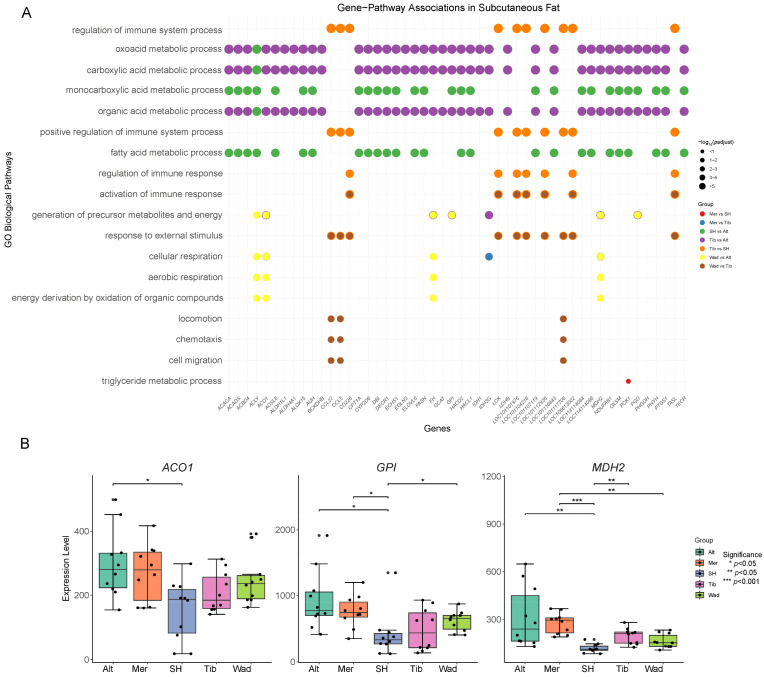
Shared core Genes and their expression validation. (**A**) Pathway–gene matrix displaying the top 50 most frequent genes from the top five significantly enriched GO biological process terms (*p*-adjusted < 0.05) per comparison. Bubbles of the same color represent genes and pathways shared across multiple groups. (**B**) Expression of *ACO1*, *GPI*, and *MDH2* in subcutaneous fat across breeds.

Using subcutaneous fat as an example, we identified significantly enriched GO terms (Biological Process ontology, *p*-adjusted < 0.05) and selected the top five enriched pathways from each comparison group. The 50 most frequently occurring genes across these pathways were subsequently analyzed. By integrating gene frequency and *p*-adjusted values, a pathway–gene bubble plot was generated to visualize the relationships between key genes and biological pathways among different groups. (Figure 4A). Due to the screening criteria applied, some comparison groups were excluded during this process. Subsequently, further filtering was performed among representative genes associated with lipid metabolism pathways, and their expression levels were compared between groups. In the subcutaneous fat tissues of the five sheep breeds, three key lipid metabolism-related genes (*ACO1*, *GPI*, and *MDH2*) exhibited significant inter-breed differences (Figure 4B). Specifically, ACO1 showed a significant difference between Altay sheep and Small-tailed Han sheep (*p* < 0.05). *ACO1* encodes aconitase, a key enzyme of the tricarboxylic acid (TCA) cycle that catalyzes the reversible conversion between citrate and isocitrate. This step plays a central role in determining the allocation of carbon flux between energy metabolism and fatty acid synthesis, thereby influencing the efficiency of lipid deposition. The observed breed-specific expression suggests potential metabolic partitioning in energy utilization and lipid biosynthesis. *GPI* displayed significant differences among Altay, Merino, and Small-tailed Han, as well as between Altay and Wadi (*p* < 0.05). *GPI* encodes glucose-6-phosphate isomerase, a rate-limiting enzyme in glycolysis and gluconeogenesis, which is crucial for regulating glucose utilization and energy supply. Variations in *GPI* expression may affect the availability of glycolytic intermediates required for triglyceride synthesis, thereby modulating breed-specific fat deposition capacities. Notably, *MDH2* showed highly significant differences between Altay and the other four breeds (Merino sheep, Small-tailed Han sheep, Tibetan sheep, and Wadi sheep) (*p* < 0.01 or *p* < 0.001). *MDH2* encodes mitochondrial malate dehydrogenase, which catalyzes the interconversion of malate and oxaloacetate in the TCA cycle, playing a pivotal role in NADH production and energy homeostasis. Differences in *MDH2* expression may directly influence the energy metabolism capacity and redox balance of adipocytes, leading to breed-specific patterns of lipid deposition and metabolic traits. Taken together, these results not only reveal distinct gene expression profiles across breeds but also highlight the potential regulatory mechanisms linking carbon metabolism, energy production, and fat deposition in a breed-specific manner.

### 3.4. Cross-Tissue Integration of Significant Pathways

To systematically and comprehensively characterize the overlap and divergence of functional pathways across different adipose depots and breeds, we further integrated the GO and KEGG enrichment results of five adipose tissues based on our prior differential expression analyses. Specifically, for each tissue, we merged the sets of significantly enriched pathways from both GO and KEGG (threshold FDR ≤ 0.05) to form a union, thereby ensuring that potential biological signals would not be omitted due to redundancy between databases.

At the global level, this cross-tissue integration and UpSet visualization clearly revealed the common and breed-specific patterns of significantly enriched pathways across the five adipose depots. However, considering that tail fat represents the most distinctive and divergent adipose depot associated with fat deposition differences among sheep, and is directly linked to the morphological and metabolic basis of fat-rumped, short fat-tailed, and thin-tailed phenotypes, we focused subsequent analyses specifically on tail fat. More precisely, the five representative breeds were further grouped into three major phenotypic categories: fat-rumped, short fat-tailed, and thin-tailed breeds.

**Figure 5:**
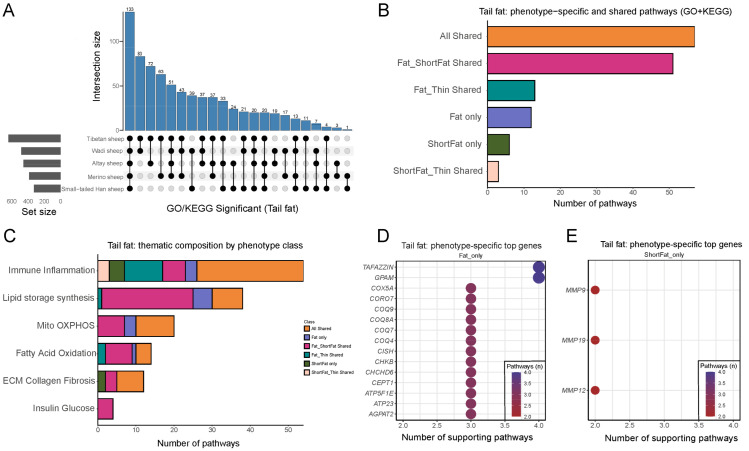
Molecular regulatory basis of distinct tail phenotypes. (**A**) UpSet plot of significantly enriched GO/KEGG pathways (FDR < 0.05) for each comparison are shown. Bubbles wrapped by different colors indicate the same group. (**B**) Distribution of pathway categories among three phenotypic groups (fat-rumped, short fat-tailed, thin-tailed). Most pathways are shared, with fat-rumped and short fat-tailed sheep showing the highest overlap. (**C**) Functional classification of pathways across phenotypes. Lipid synthesis and storage are enhanced in fat-rumped and short fat-tailed sheep, oxidative phosphorylation is enriched in fat-rumped sheep, while ECM remodeling is prominent in short fat-tailed sheep. (**D**) Fat-rumped sheep specific genes, including *TAFAZZIN*, *GPAM*, *GCDH*, *ETFDH*, *COX5A*, *COQ* family, and *ATP5F1E*, mainly involved in mitochondrial metabolism and lipid biosynthesis. (**E**) Short fat-tailed sheep specific genes, including *MMP9*, *MMP12*, and *MMP19*, highlighting ECM remodeling as a key regulatory feature.

Figure 5A presents the overlap and divergence of significantly enriched GO/KEGG pathways (FDR ≤ 0.05) in the tail fat of the five sheep breeds. The tallest bar corresponds to 133 pathways shared by all five breeds, indicating a set of core pathways widely involved in lipid metabolism and signaling regulation, serving as a conserved molecular foundation across breeds. In addition, several other large intersection bars demonstrate that subsets of pathways are consistently present in the majority of breeds. Meanwhile, a number of smaller intersections correspond to pathways unique to specific breeds, or shared only between two breeds. Overall, Figure 5A highlights that tail fat harbors both a broad spectrum of conserved metabolic pathways and a certain number of breed-specific or partially shared pathways. These unique pathways may be closely associated with the formation of different tail phenotypes and their metabolic features.

### 3.5. Identification of Tail Fat Specific Pathways and Phenotype Associated Genes

After classifying the enriched pathways in tail fat according to the three phenotypic groups, we first compared the overall distribution of pathway categories (Figure 5B). The results showed that the majority of significant pathways belonged to the universally shared category, indicating the presence of a core set of conserved networks broadly involved in lipid metabolism and signaling regulation across all tail types. In pairwise comparisons, fat-rumped and short fat-tailed sheep exhibited the highest number of shared pathways, whereas overlaps involving thin-tailed sheep were markedly fewer, underscoring the closer functional resemblance between fat-rumped and short fat-tailed phenotypes. This pattern is consistent with their shared phenotypic trait of higher fat deposition. In contrast, thin-tailed sheep exhibited fewer overlapping pathways with the other two groups, highlighting its distinctiveness in tail fat metabolic pathways. Although phenotype-specific pathways were fewer in number, they often converged on a limited set of key pathways, suggesting that these unique signals may exert stronger phenotype-driving effects.

To further dissect these differences, we categorized the significant pathways of tail fat into major functional themes and compared their distribution across the three phenotypic groups (Figure 5C). The detailed pathway lists are provided in Table S3. Immune and inflammation-related pathways were broadly enriched across all phenotypes, with the vast majority falling into the shared category, indicating that low-grade inflammation and immune signaling constitute a common molecular background of tail fat. Lipid synthesis and storage pathways were predominantly enriched in the fat-rumped and short fat-tailed shared class, with additional fat-tail-specific signals, suggesting consistent enhancement of lipid droplet formation and triglyceride accumulation in these two groups, while such pathways were relatively attenuated in thin-tailed sheep. Mitochondrial oxidative phosphorylation pathways were also mainly shared, but with additional enrichment of sub-pathways in the fat-rumped group, reflecting a higher energy demand under increased synthetic load. Fatty acid oxidation pathways were enriched in both the shared category and fat-rumped and thin-tailed shared class, indicating that thin-tailed sheep retain a robust oxidative capacity and share certain features with fat-rumped sheep, while remaining distinct from short fat-tailed sheep. Extracellular matrix (ECM) and fibrosis-related pathways were generally present in the shared category, although short fat-tailed sheep displayed mild specificity in localized ECM remodeling pathways, potentially linked to structural tissue adaptation. Insulin and glucose metabolism pathways were the least represented and almost exclusively appeared in the shared category, suggesting they play a relatively stable role across all phenotypes and are unlikely to drive tail-type divergence. While these pathway-level differences highlight distinct molecular landscapes among phenotypes, their functional impacts are ultimately mediated through specific genes.

Subsequent analysis of genes associated with tail fat-specific pathways revealed multiple candidate genes potentially involved in tail-type determination. In fat-rumped specific pathways, 20 core genes were repeatedly identified across multiple enriched terms, such as *TAFAZZIN*, *GPAM*, *GCDH*, *ETFDH*, *COX5A*, members of the *COQ* family (*COQ4*, *COQ7*, *COQ8A*, *COQ9*), and *ATP5F1E* (Figure 5D). These genes are primarily involved in mitochondrial function, oxidative phosphorylation, fatty acid metabolism, and triglyceride biosynthesis, indicating that the formation of the fat-rumped phenotype may be closely associated with enhanced mitochondrial energy efficiency and lipid synthetic capacity. In contrast, short fat-tailed specific pathways yielded a distinct set of representative genes, mainly *MMP9*, *MMP12*, *MMP19* (Figure 5E). The *MMP* family genes are strongly associated with ECM remodeling and tissue reconstruction, suggesting that short fat-tailed sheep may rely more on ECM remodeling and localized structural adaptations rather than metabolic regulation alone to shape their tail fat phenotype. Additionally, fat-rumped sheep-specific genes predominantly cluster in energy metabolism and lipid storage processes, whereas short fat-tailed sheep-specific genes are more closely linked to structural and ECM regulation. Such mechanistic divergence may underlie the fundamental differences driving the development of these two tail types. Although thin-tailed sheep did not exhibit significantly enriched phenotype-specific genes in this analysis, our earlier findings suggest that their phenotype may be maintained through the absence of active lipid synthesis-associated genes, thereby restricting fat accumulation. Collectively, these results not only highlight distinct molecular mechanisms between fat-rumped and short fat-tailed phenotypes but also identify a set of promising candidate genes (*TAFAZZIN*, *GPAM*, *COQ* family, and *MMP* family) that may serve as potential targets for functional validation and molecular breeding aimed at regulating fat deposition and tail-type traits in sheep.

## 4. Discussion

In mammals, adipose tissue serves multiple physiological functions [22]. As a major site of energy storage, it acts as an energy buffer by accumulating lipids during periods of nutrient availability and releasing fatty acids for systemic utilization during fasting or high energy demand [23,24]. This large-scale transcriptomic study of five sheep breeds and five adipose depots provided a comprehensive view of the transcriptional basis of adipose heterogeneity and tail phenotype diversification. We showed that variation in tail morphology, ranging from fat-rumped to short fat-tailed to thin-tailed types, appeared to driven by distinct molecular pathways. A conserved set of metabolic and immune pathways was shared across all phenotypes, while fat-rumped sheep were enriched in mitochondrial oxidative phosphorylation and triglyceride synthesis genes (*TAFAZZIN*, *GPAM*, *COQ* family), and short fat-tailed sheep showed unique extracellular matrix remodeling signatures (*MMP9*, *MMP12*). These results suggest that similar fat deposition phenotypes can arise through either enhanced metabolic activity or adaptation. Thin-tailed sheep, in contrast, appeared to lacked these pro-lipogenic and ECM remodeling programs. Our findings deepened the understanding of tail fat biology and highlighted candidate genes potentially relevant for genetic improvement in sheep.

We observed marked differences in DEGs and enriched functions among caul, subcutaneous, perirenal, intermuscular, and tail fat highlighted distinct physiological roles [25]. Subcutaneous and intermuscular fat showed abundant DEGs enriched in lipid metabolism, indicating high responsiveness to genetic background and major roles in regulating energy storage and mobilization [26]. In contrast, caul and tail fat displayed greater transcriptional conservation, suggesting stricter functional regulation, with immune-related pathways in caul fat consistent with its role as a visceral depot involved in immune surveillance [27]. Such depot-specificity is essential for interpreting breed differences, as the same comparison may yield divergent results depending on the tissue examined.

The tail represents a major site of fat storage in fat-rumped sheep, and uncovering the key genes involved in this process is essential for elucidating the molecular mechanisms underlying fat-tail formation in sheep [20]. Focusing on tail fat, we discovered a complex interplay between conserved and breed-specific pathways. The large set of pathways shared across all five breeds represents the fundamental biological machinery required to establish and maintain a functional adipose tissue, including core lipid metabolism, energy production, and baseline immune function. This shared foundation underscores that tail fat, regardless of its ultimate size, is a bona fide adipose depot operating on common principles [28]. However, the true drivers of phenotypic divergence lie in the differences superimposed upon this common background.

By grouping breeds into phenotypic categories, we detected a pronounced compelling molecular stratification. The fat-rumped phenotype, represented by Altay sheep, was associated with an upregulation of mitochondrial energy metabolism and lipid biosynthetic capacity. Key genes identified in this group included *TAFAZZIN*, which is required for cardiolipin biosynthesis and maintaining mitochondrial membrane integrity [29], and *GPAM*, which catalyzes the first committed step in glycerolipid biosynthesis [30]. In addition, several members of the coenzyme Q biosynthesis family, including *COQ4*, *COQ7*, *COQ8A*, and *COQ9*, were found to be critical for sustaining electron transport chain function and efficient ATP production [31]. Previous studies have shown that *ETFDH* plays an indispensable role in fatty acid β-oxidation, thereby linking energy metabolism directly to lipid utilization [32]. The coordinated enhancement of these mitochondrial and lipid metabolic genes indicated that fat-rumped sheep may have acted as a highly efficient enhancement of the “metabolism engine”, in which robust oxidative phosphorylation not only provided sufficient energy for active lipogenesis but may also act as a regulatory signal to promote triglyceride accumulation and adipocyte hypertrophy [33,34]. These findings are consistent with previous studies emphasizing the role of transcriptional regulators such as *PPARγ* and lipid biosynthetic pathways in promoting fat deposition in sheep [35,36]. Moreover, similar observations have been reported in other livestock species, where mitochondrial activity and coenzyme Q related pathways were shown to contribute to efficient fat storage and adaptive energy regulation [37], highlighting the evolutionary significance of these mechanisms in animals exposed to cold or nutritionally challenging environments.

In contrast, the short fat-tailed phenotype, represented by Small-tailed Han sheep and Wadi sheep breeds, showed a distinct molecular profile dominated by Matrix Metalloproteinases (*MMP9*, *MMP12* and *MMP19*). These zinc-dependent endopeptidases are key regulators of extracellular matrix (ECM) degradation and remodeling [38], suggesting that tail fat deposition in this phenotype depends not only on metabolism but also on structural plasticity [39]. ECM breakdown and reorganization are essential for adipocyte hyperplasia and hypertrophy, enabling lipid accumulation and neovascularization [40]. Thus, while fat-rumped sheep achieve efficient lipid storage through metabolism, short fat-tailed sheep rely on ECM remodeling to enhance adipocyte proliferation and expansion. This highlights ECM dynamics as an underexplored regulator of tail fat deposition, with additional evidence linking tissue microenvironment and mechanical properties to adipogenesis [41]. By contrast, the thin-tailed phenotype exhibited fewer unique pathways, reflecting a lack of pro-lipogenic and ECM remodeling programs. Although fat-rumped and thin-tailed sheep shared some fatty acid oxidation pathways, Merino sheep appear to lack the anabolic drive, resulting in limited fat accumulation [42].

Our study complemented previous transcriptomic research on tail fat in sheep and provided new mechanistic insights. We confirmed the involvement of triglyceride synthesis pathways, as reported in comparisons between Small-tailed sheep and Dorset sheep [11], but further demonstrated that tail types differed in how strongly they activate these pathways, and that distinct biological processes such as extracellular matrix (ECM) remodeling also play crucial roles. Candidate genes such as *GPAM* and *COQ* family members highlighted enhanced lipid biosynthesis and mitochondrial metabolism in fat-rumped sheep, whereas *MMP* family genes suggested to structural remodeling as a hallmark of short fat-tailed phenotypes [43]. Biologically, our results support a dual-axis model of tail fat development: a metabolic axis (mitochondrial energy and lipid synthesis) and a structural axis (ECM remodeling) [44]. Different breeds likely represent different evolutionary combinations of these mechanisms, leading to the observed diversity in tail phenotypes. From an applied perspective, these candidate genes provide promising molecular targets for breeding strategies, such as enhancing metabolic drivers (*GPAM*, *COQ8A*) to promote fat deposition or modulating ECM regulators (*MMP9*, *MMP12*) to optimize tissue composition. Nevertheless, limitations must be noted. Public RNA-seq data may contain batch effects, and transcriptomics alone could not establish causality [17]. Future work should focus on functional validation of these genes and integrate multi-omics approaches under controlled environmental conditions to clarify regulatory mechanisms.

## 5. Conclusions

Based on the findings, we propose that fat-rumped sheep are primarily driven by a hyperactive metabolic engine characterized by enhanced mitochondrial energy metabolism and robust lipid biosynthetic activity, which together facilitate efficient triglyceride accumulation in adipocytes. In contrast, short fat-tailed sheep appear to rely more heavily on extracellular matrix (ECM) remodeling, providing a structural environment that promotes adipocyte proliferation and tissue expansion rather than purely metabolic enhancement. Thin-tailed sheep, however, exhibit an absence of these metabolic and structural reinforcements, leading to minimal tail fat accumulation. Beyond the tail-specific mechanisms, our multi-tissue transcriptomic survey underscores a generalized principle of adipose tissue specialization. We observed that subcutaneous and intermuscular fat depots exhibited the highest transcriptional responsiveness to breed identity, underscoring their roles as primary and malleable sites for systemic energy storage and mobilization. In contrast, caul fat demonstrated greater transcriptional conservation across breeds but a distinct enrichment for immune-related pathways, highlighting its unique role as a visceral fat depot integrally involved in metabolism. This conceptual framework refines our understanding of adipose tissue biology, highlights distinct evolutionary strategies for fat storage across tail phenotypes, and provides concrete genetic targets that can be leveraged for molecular breeding programs aimed at improving fat deposition, adaptive resilience, and production traits in sheep.

## Data Availability

The original contributions presented in the study are included in the article/Supplementary Material, further inquiries can be directed to the corresponding author.

## Supplementary Materials

The following supporting information can be downloaded at: https://www.mdpi.com/article/10.3390/genes17010093/s1, Table S1. The basic statistics for the RNA sequencing of the five adipose tissues in these breeds. Table S2. GO and KEGG enrichment results for pairwise breed comparisons across five adipose tissues. Table S3. Combining different pathways into several major pathway categories. Table S4. Metadata of the 250 RNA-seq samples used in this study. Table S5. Statistical summary of differential expression analyses for each pairwise comparison. Table S6. Complete lists of differentially expressed genes (DEGs) for all pairwise comparisons. Figures S1–S5. Volcano plots for differential expression analysis in each adipose tissue. Figures S6–S10. MA plots for differential expression analysis in each adipose tissue. Figures S11–S15. *p*-value histograms for differential expression tests in each adipose tissue.
