# Supplementary material for: Transcriptome Analysis of Adipose Tissues from Five Sheep Breeds Reveals Key Genes Involved in Fat Deposition
Source: Genes (Basel). 2026 Jan 17;17(1):93. doi: 10.3390/genes17010093 (PMC12841507; doi:10.3390/genes17010093)
Supplement: Supplementary file 1 [file genes-17-00093-s001.zip › Supplementary Figures/Supplementary Figure 11.pdf]

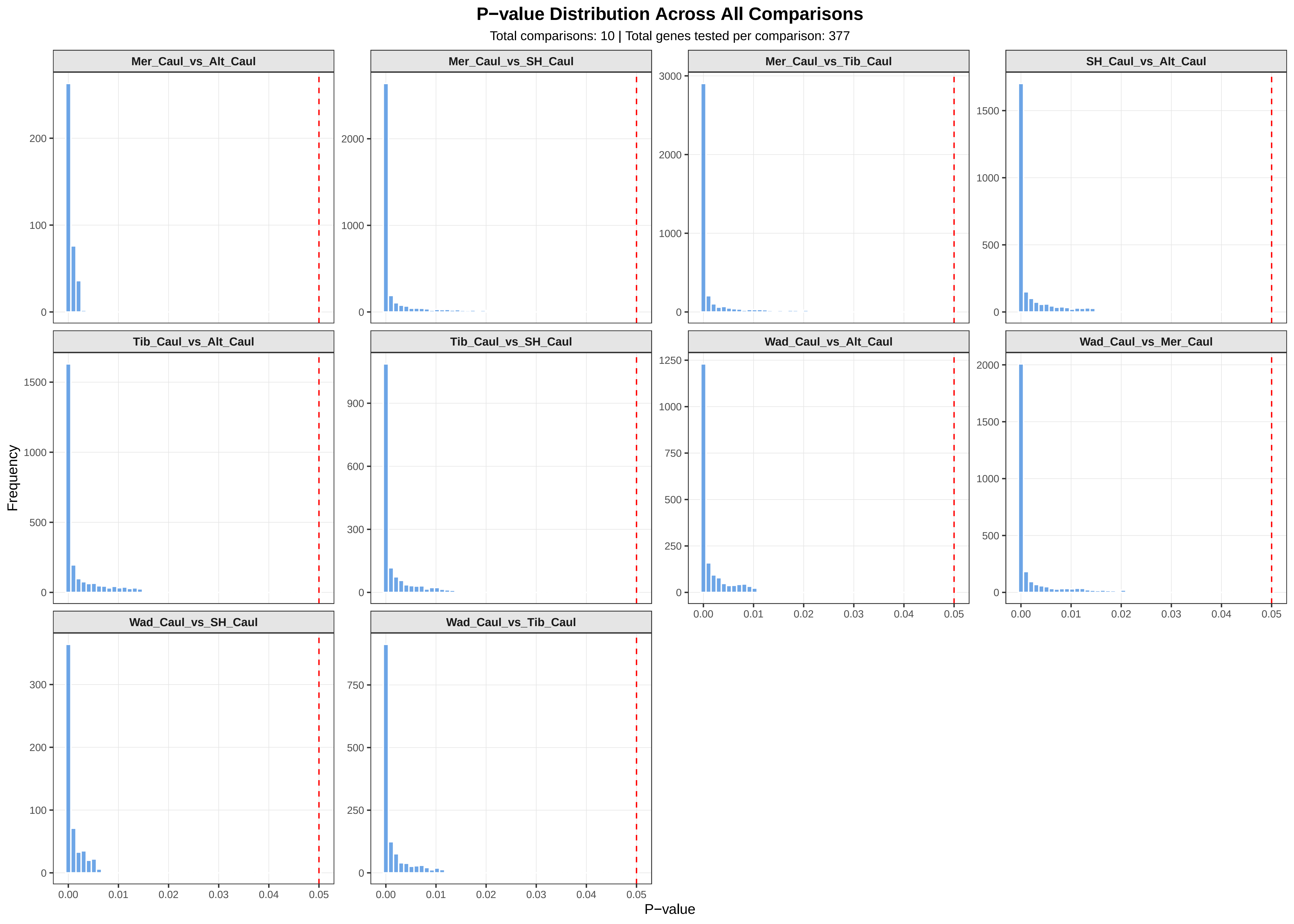

P−value Distribution Across All Comparisons
Total comparisons: 10 | Total genes tested per comparison: 377
Mer_Caul_vs_Alt_Caul
Mer_Caul_vs_SH_Caul
Mer_Caul_vs_Tib_Caul
SH_Caul_vs_Alt_Caul
Tib_Caul_vs_Alt_Caul
Tib_Caul_vs_SH_Caul
Wad_Caul_vs_Alt_Caul
Wad_Caul_vs_Mer_Caul
Wad_Caul_vs_SH_Caul
Wad_Caul_vs_Tib_Caul
Frequency
P−value
0.00
0.01
0.02
0.03
0.04
0.05
