# Supplementary material for: Transcriptome Analysis of Adipose Tissues from Five Sheep Breeds Reveals Key Genes Involved in Fat Deposition
Source: Genes (Basel). 2026 Jan 17;17(1):93. doi: 10.3390/genes17010093 (PMC12841507; doi:10.3390/genes17010093)
Supplement: Supplementary file 1 [file genes-17-00093-s001.zip › Supplementary Figures/Supplementary Figure 12.pdf]

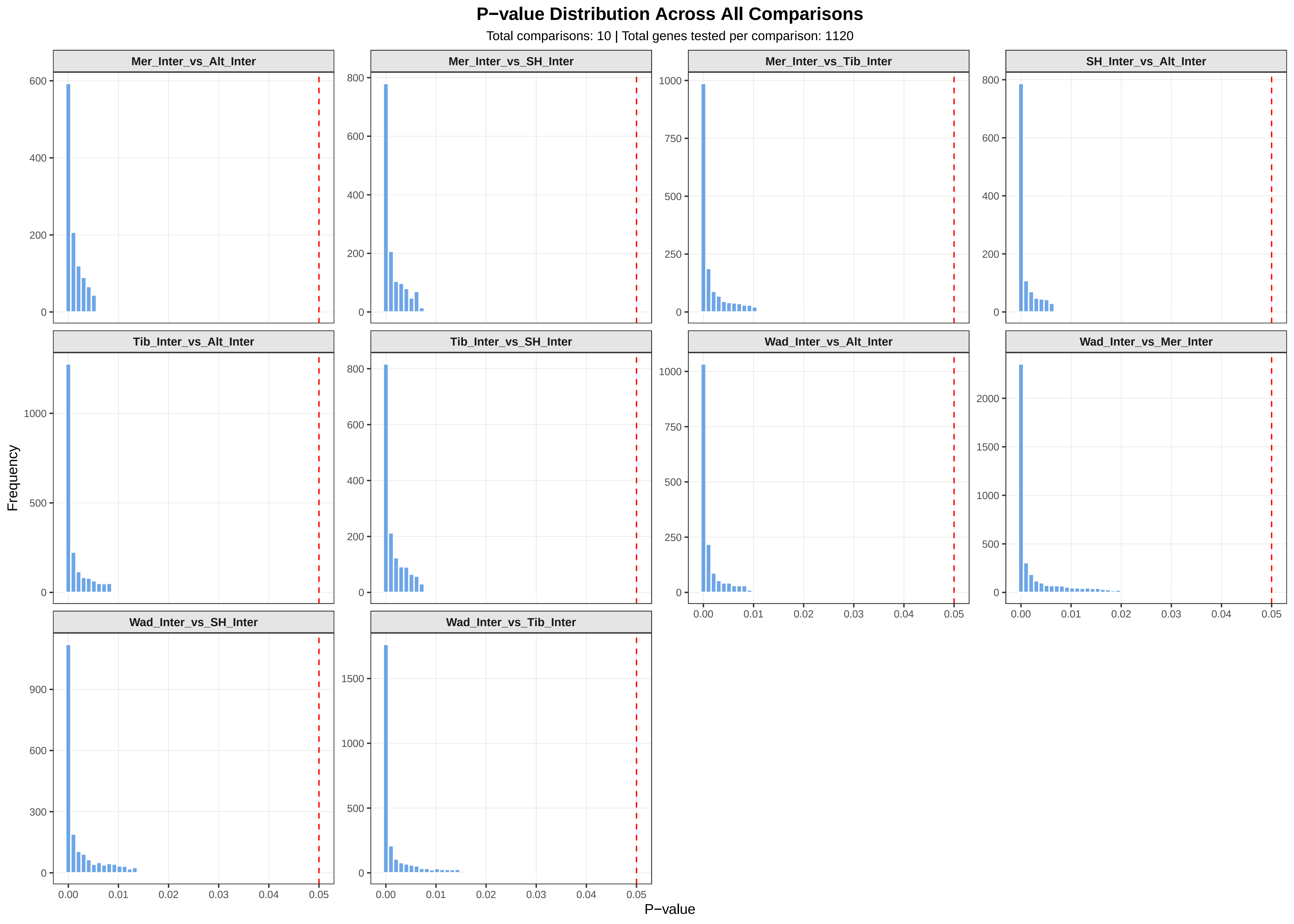

P−value Distribution Across All Comparisons
Total comparisons: 10 | Total genes tested per comparison: 1120
Mer_Inter_vs_Alt_Inter
Mer_Inter_vs_SH_Inter
Mer_Inter_vs_Tib_Inter
SH_Inter_vs_Alt_Inter
Tib_Inter_vs_Alt_Inter
Tib_Inter_vs_SH_Inter
Wad_Inter_vs_Alt_Inter
Wad_Inter_vs_Mer_Inter
Wad_Inter_vs_SH_Inter
Wad_Inter_vs_Tib_Inter
Frequency
P−value
0.00 0.01 0.02 0.03 0.04 0.05
