# Supplementary material for: Transcriptome Analysis of Adipose Tissues from Five Sheep Breeds Reveals Key Genes Involved in Fat Deposition
Source: Genes (Basel). 2026 Jan 17;17(1):93. doi: 10.3390/genes17010093 (PMC12841507; doi:10.3390/genes17010093)
Supplement: Supplementary file 1 [file genes-17-00093-s001.zip › Supplementary Figures/Supplementary Figure 13.pdf]

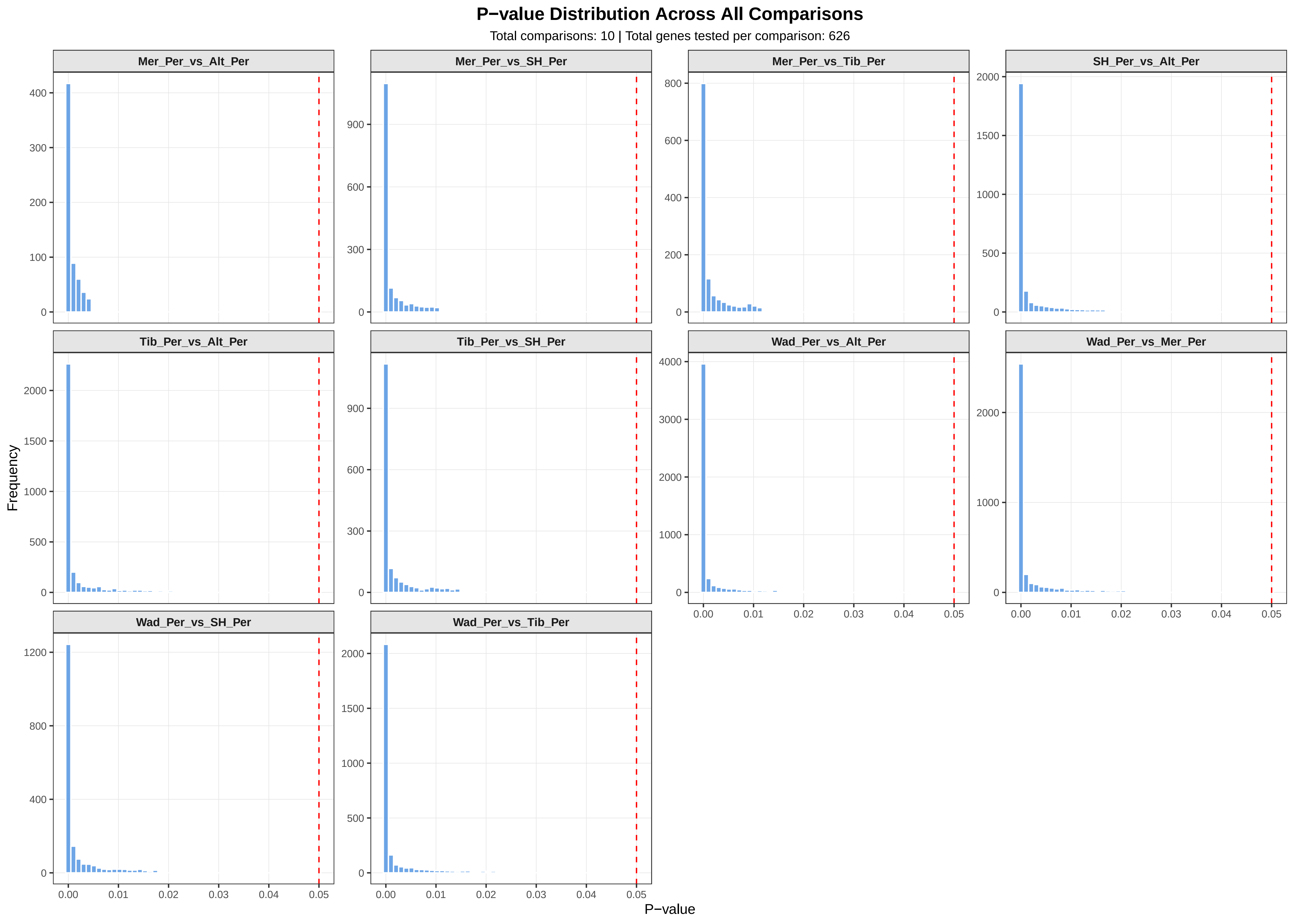
P−value Distribution Across All Comparisons
Total comparisons: 10 | Total genes tested per comparison: 626
Mer_Per_vs_Alt_Per
Mer_Per_vs_SH_Per
Mer_Per_vs_Tib_Per
SH_Per_vs_Alt_Per
Tib_Per_vs_Alt_Per
Tib_Per_vs_SH_Per
Wad_Per_vs_Alt_Per
Wad_Per_vs_Mer_Per
Wad_Per_vs_SH_Per
Wad_Per_vs_Tib_Per
Frequency
P−value
