# Supplementary material for: Transcriptome Analysis of Adipose Tissues from Five Sheep Breeds Reveals Key Genes Involved in Fat Deposition
Source: Genes (Basel). 2026 Jan 17;17(1):93. doi: 10.3390/genes17010093 (PMC12841507; doi:10.3390/genes17010093)
Supplement: Supplementary file 1 [file genes-17-00093-s001.zip › Supplementary Figures/Supplementary Figure 15.pdf]

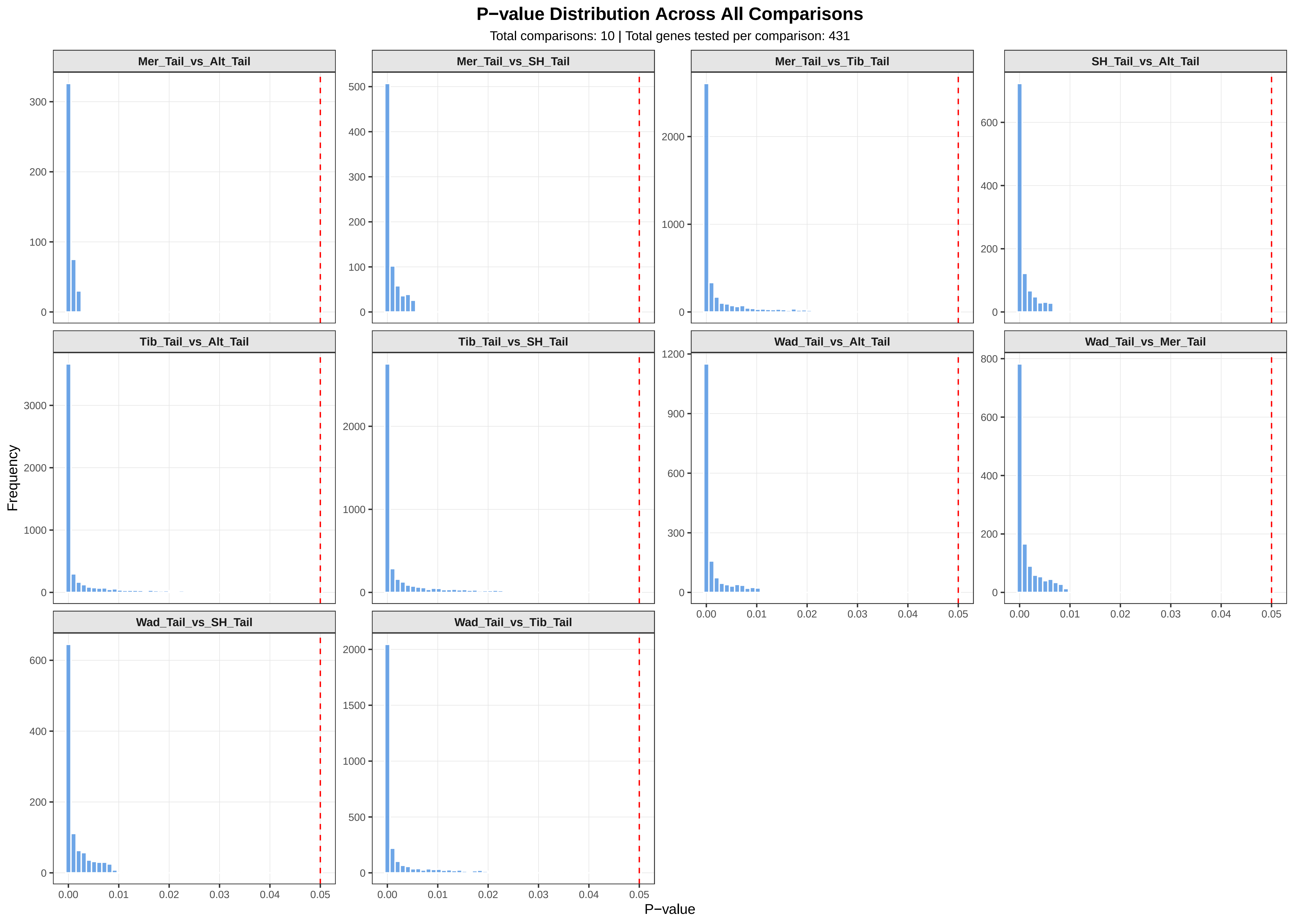

P−value Distribution Across All Comparisons
Total comparisons: 10 | Total genes tested per comparison: 431
Mer_Tail_vs_Alt_Tail
Mer_Tail_vs_SH_Tail
Mer_Tail_vs_Tib_Tail
SH_Tail_vs_Alt_Tail
Tib_Tail_vs_Alt_Tail
Tib_Tail_vs_SH_Tail
Wad_Tail_vs_Alt_Tail
Wad_Tail_vs_Mer_Tail
Wad_Tail_vs_SH_Tail
Wad_Tail_vs_Tib_Tail
Frequency
P−value
0.00
0.01
0.02
0.03
0.04
0.05
