# Supplementary figures and images for: Transcriptome Analysis of Adipose Tissues from Five Sheep Breeds Reveals Key Genes Involved in Fat Deposition
Source: Genes (Basel). 2026 Jan 17;17(1):93. doi: 10.3390/genes17010093 (PMC12841507; doi:10.3390/genes17010093)

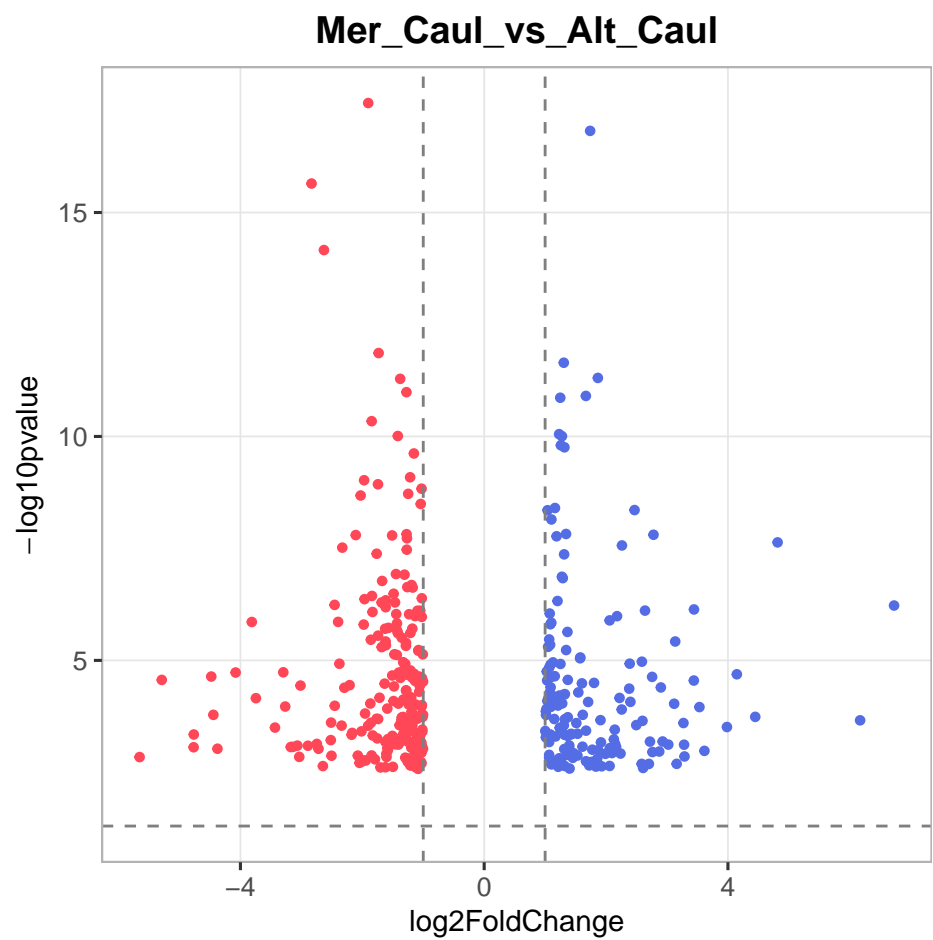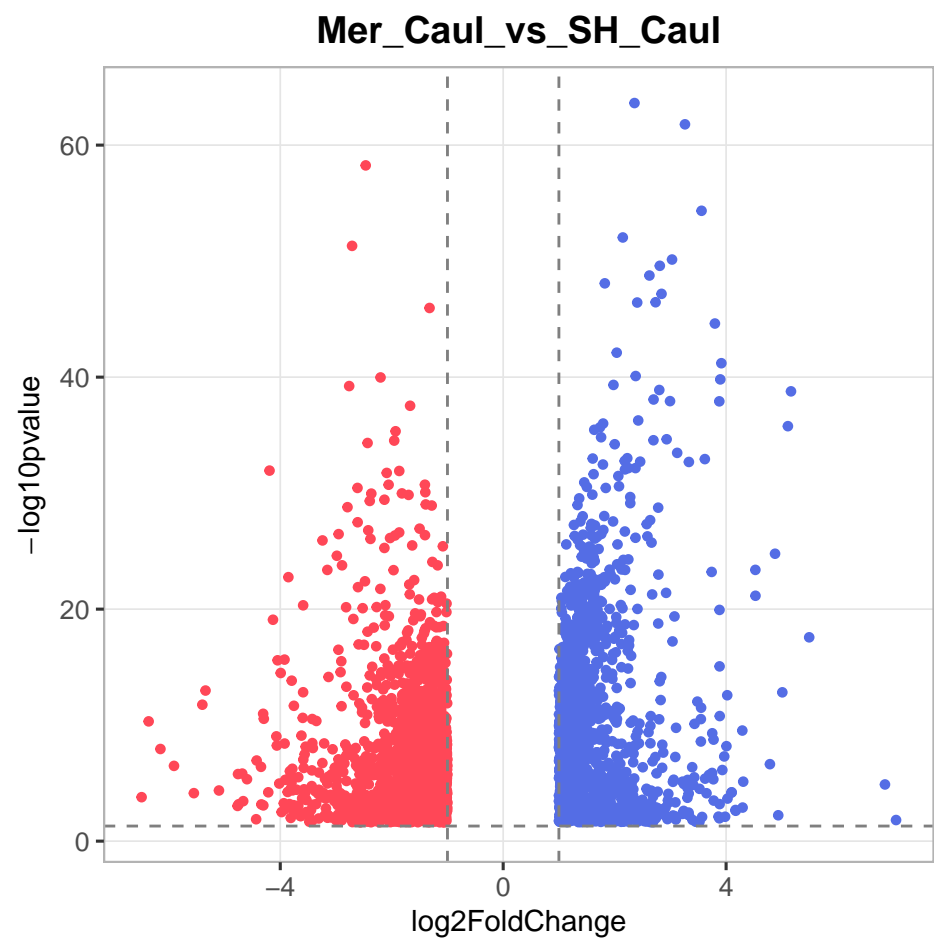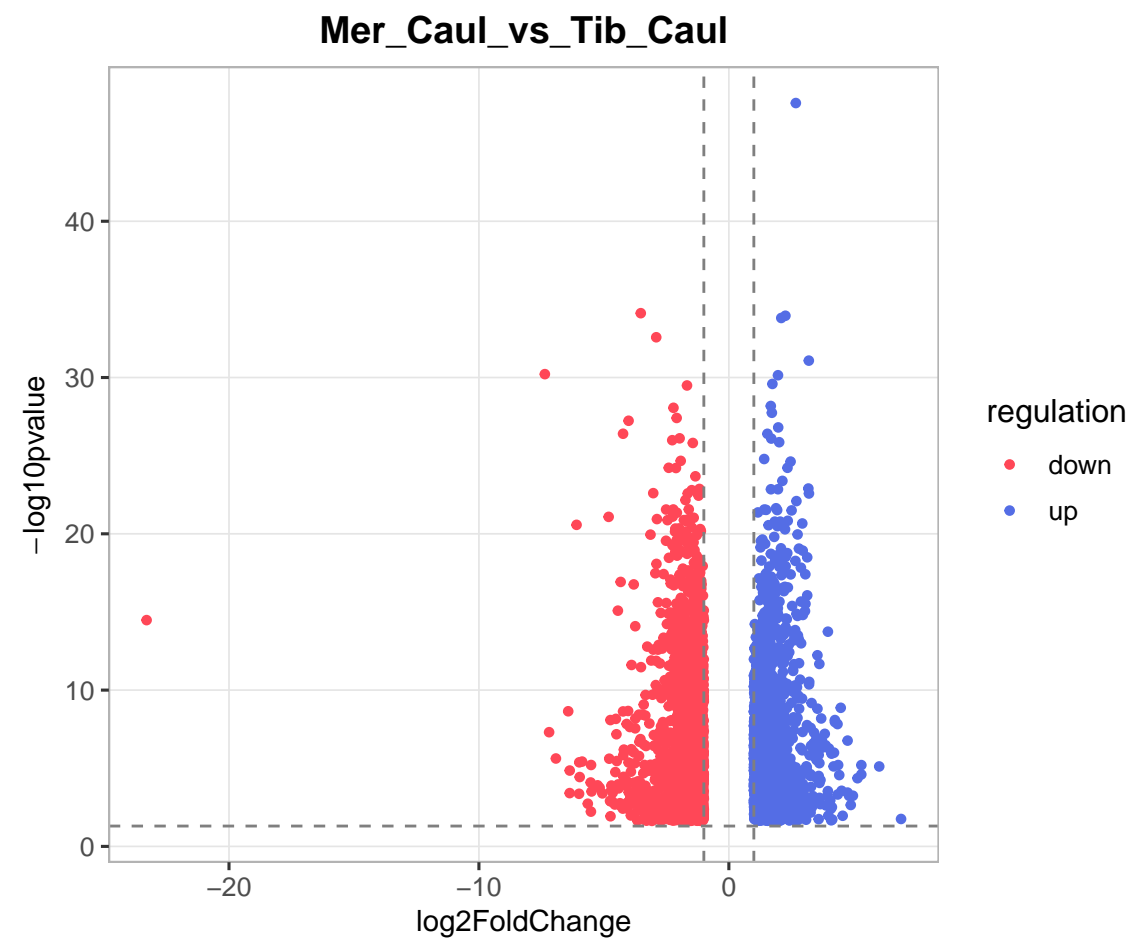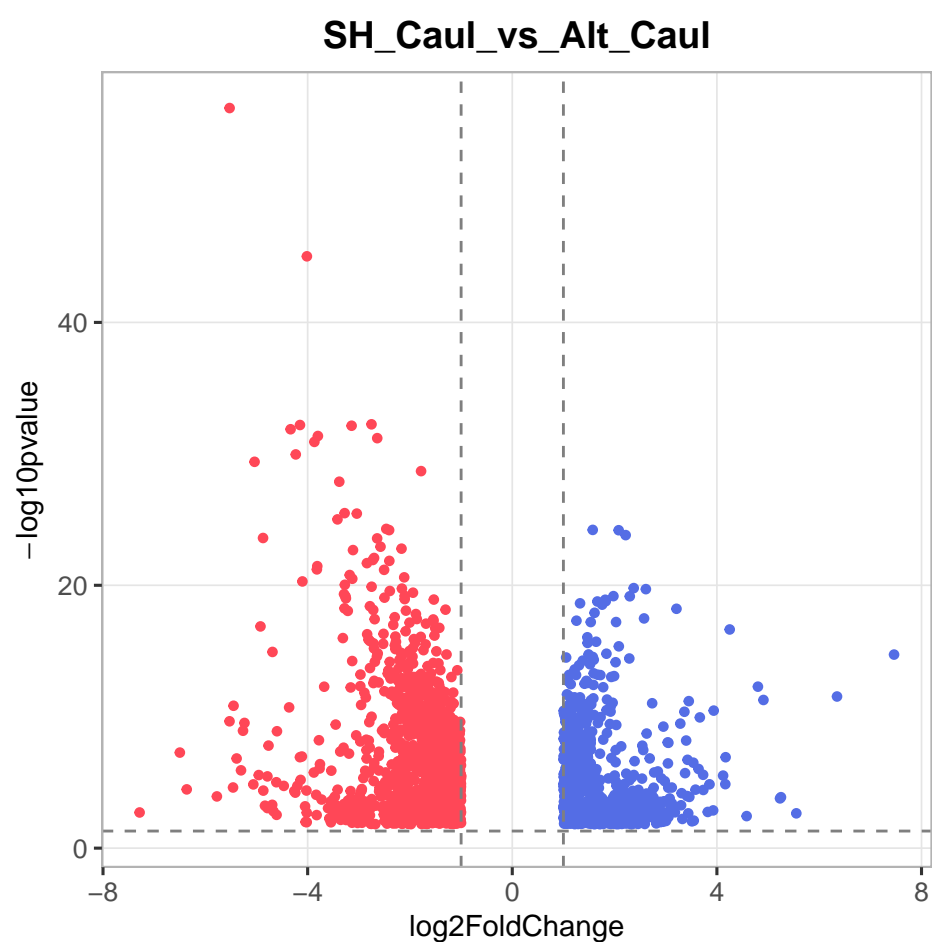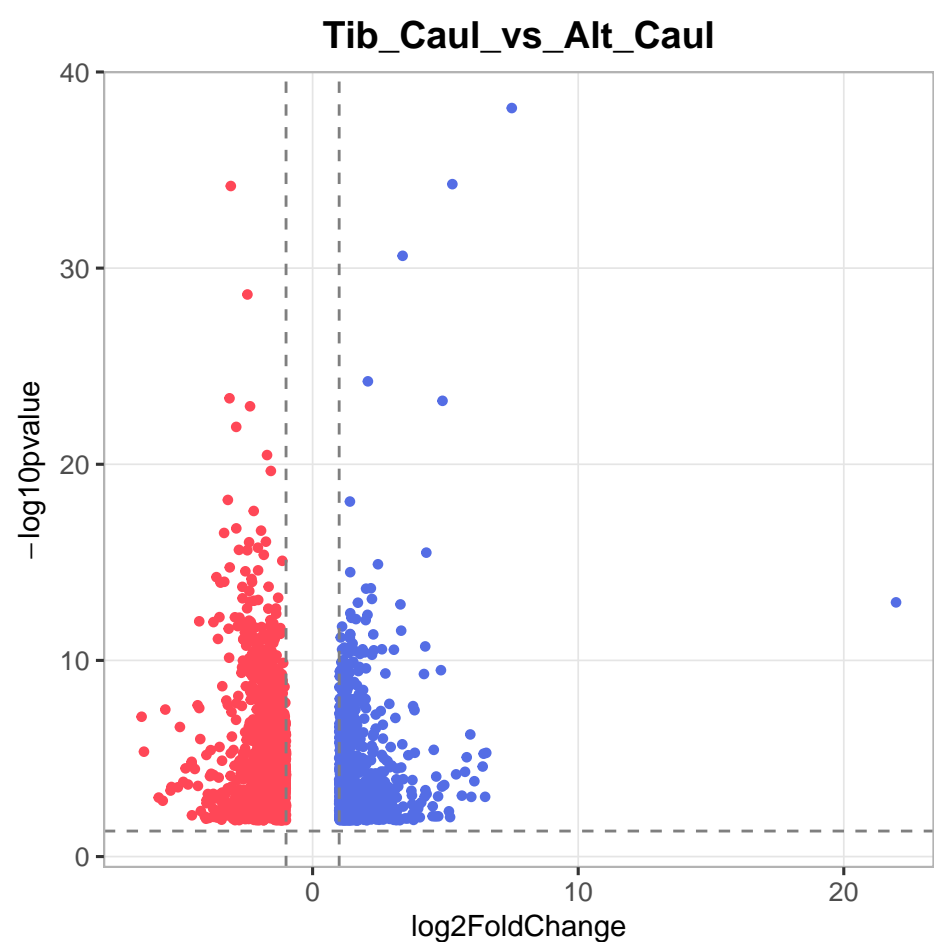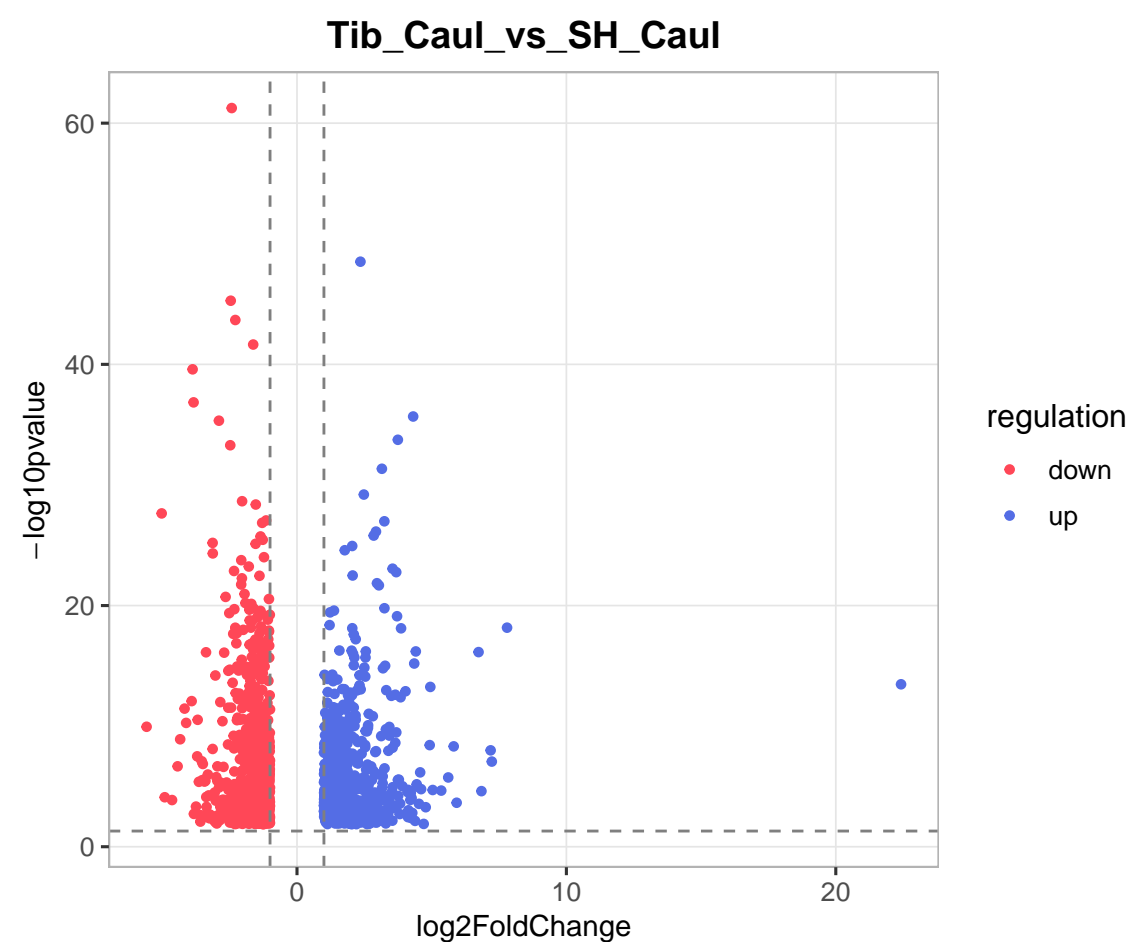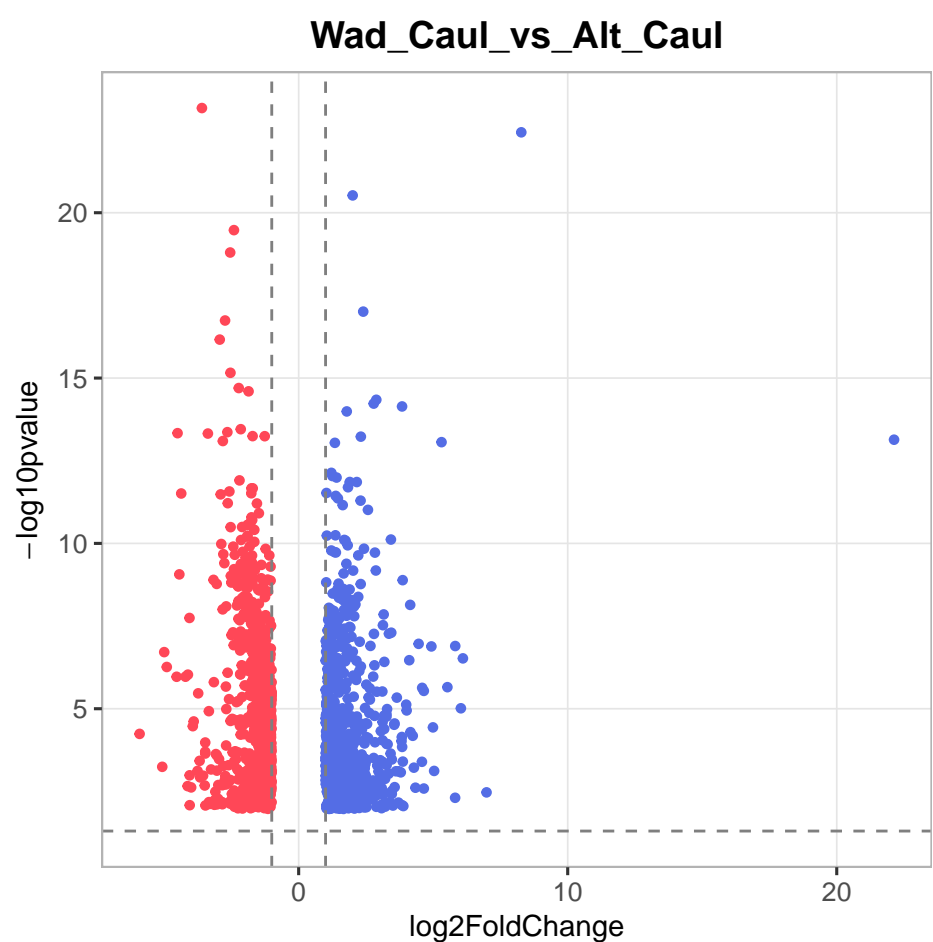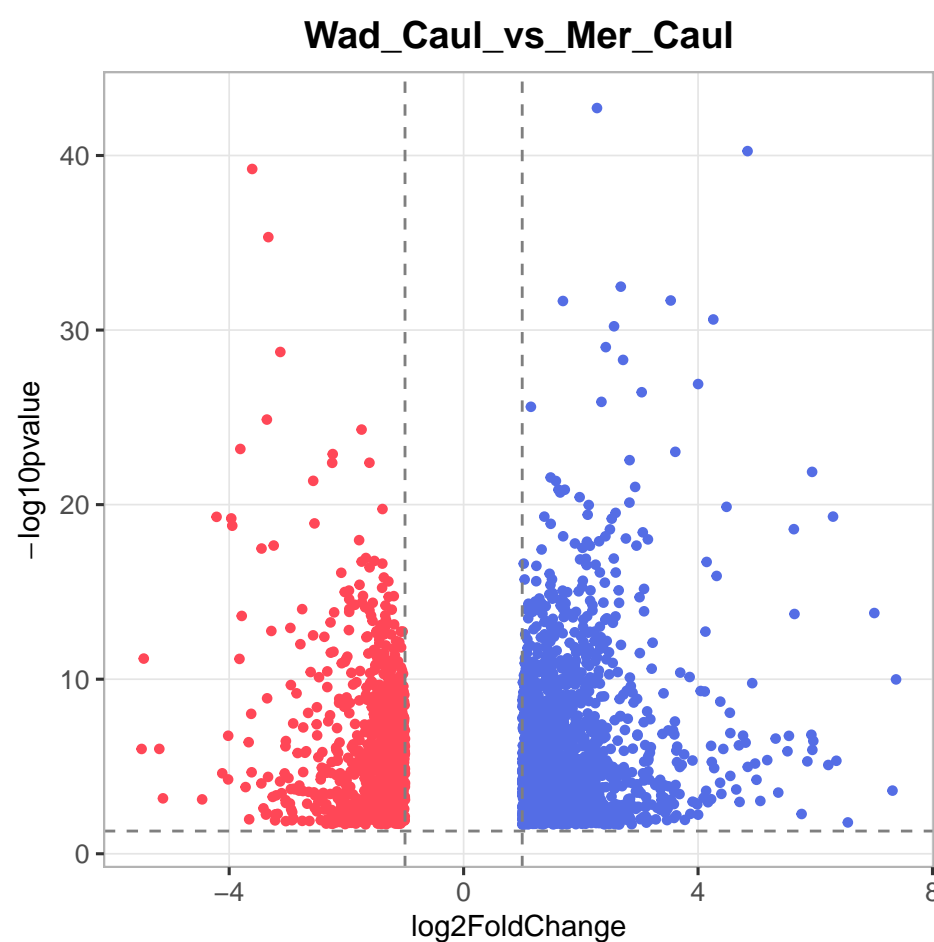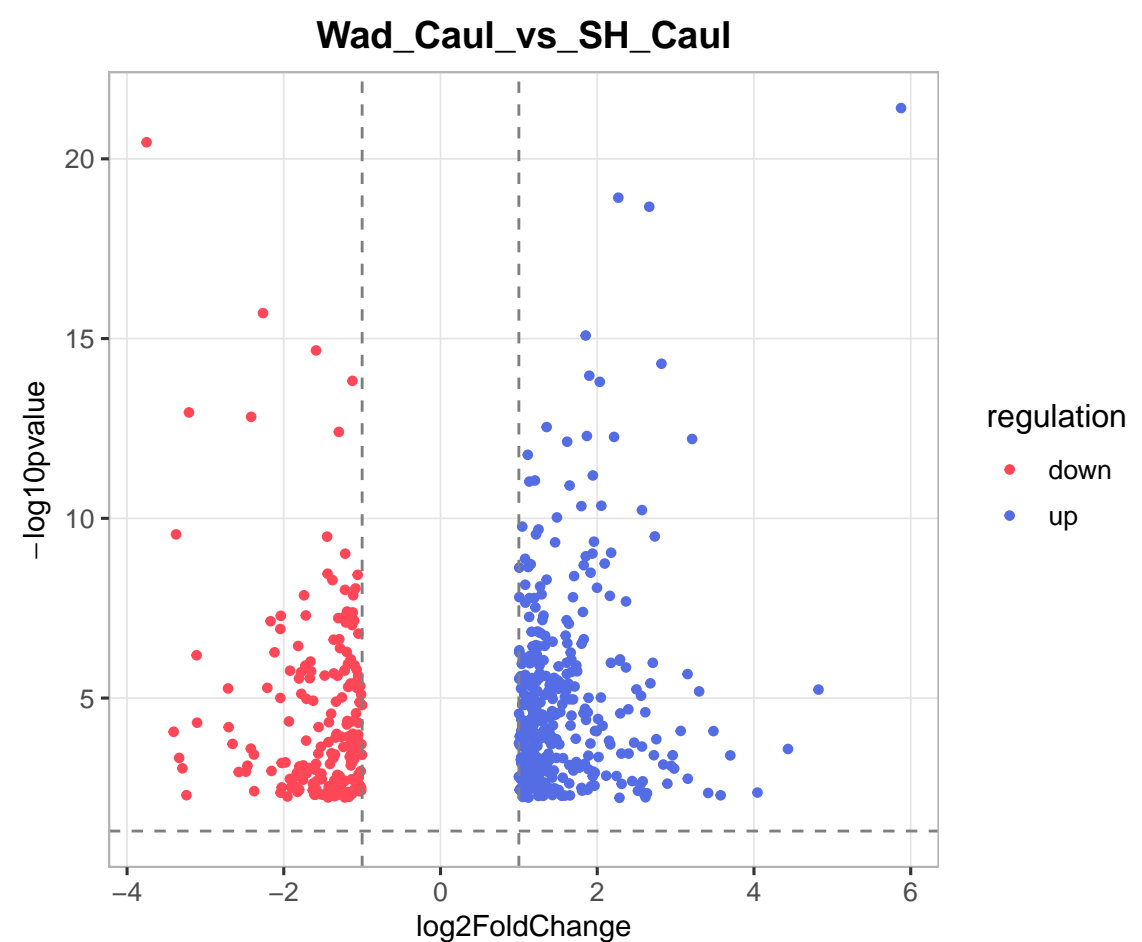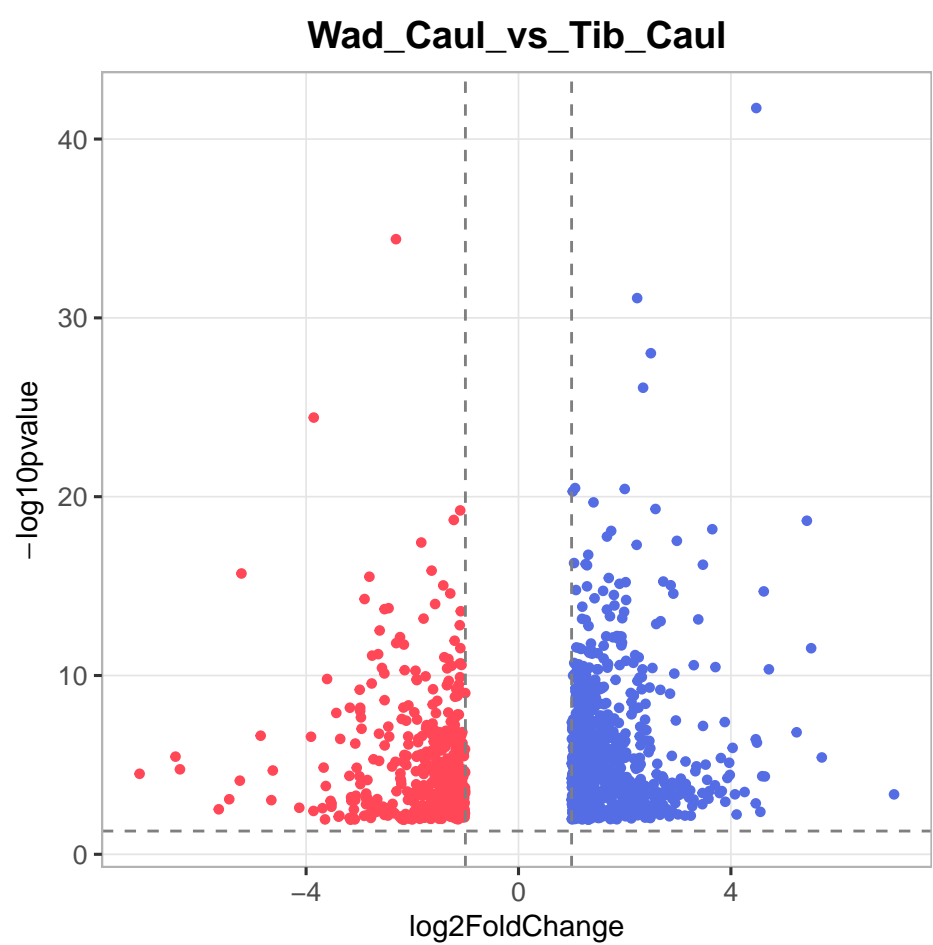

Supplement: Supplementary file 1 [file genes-17-00093-s001.zip › Supplementary Figures/Supplementary Figure 1.pdf]

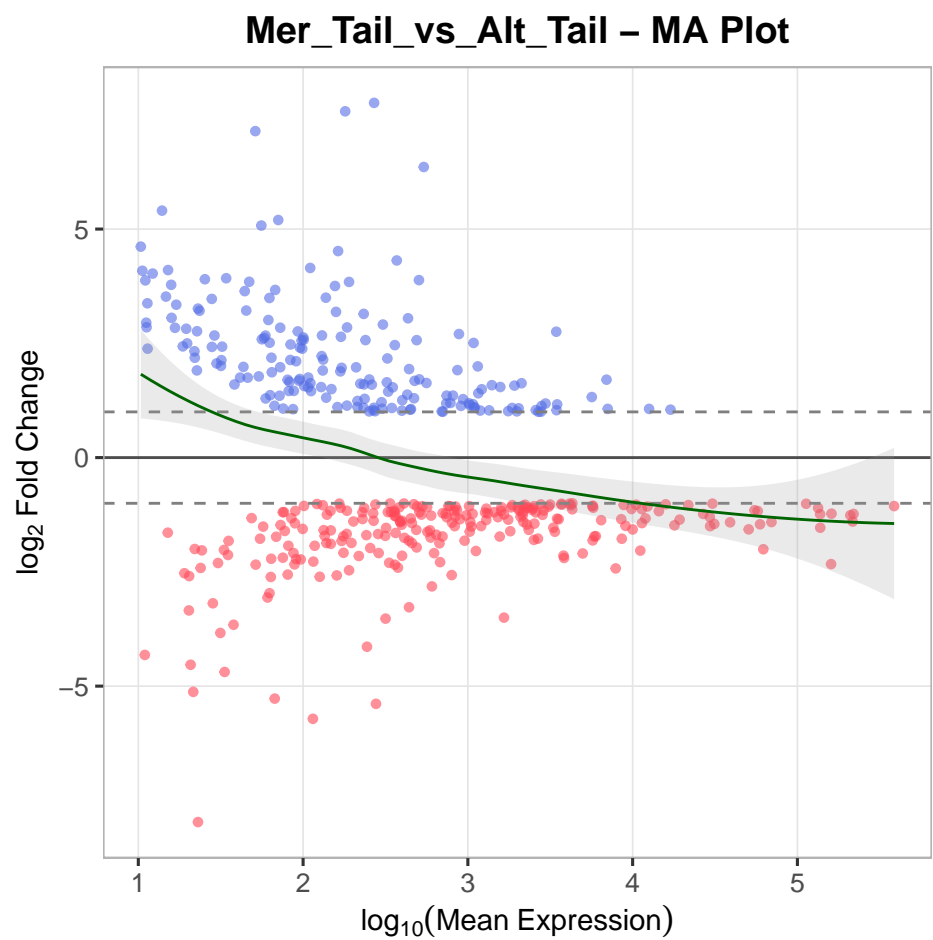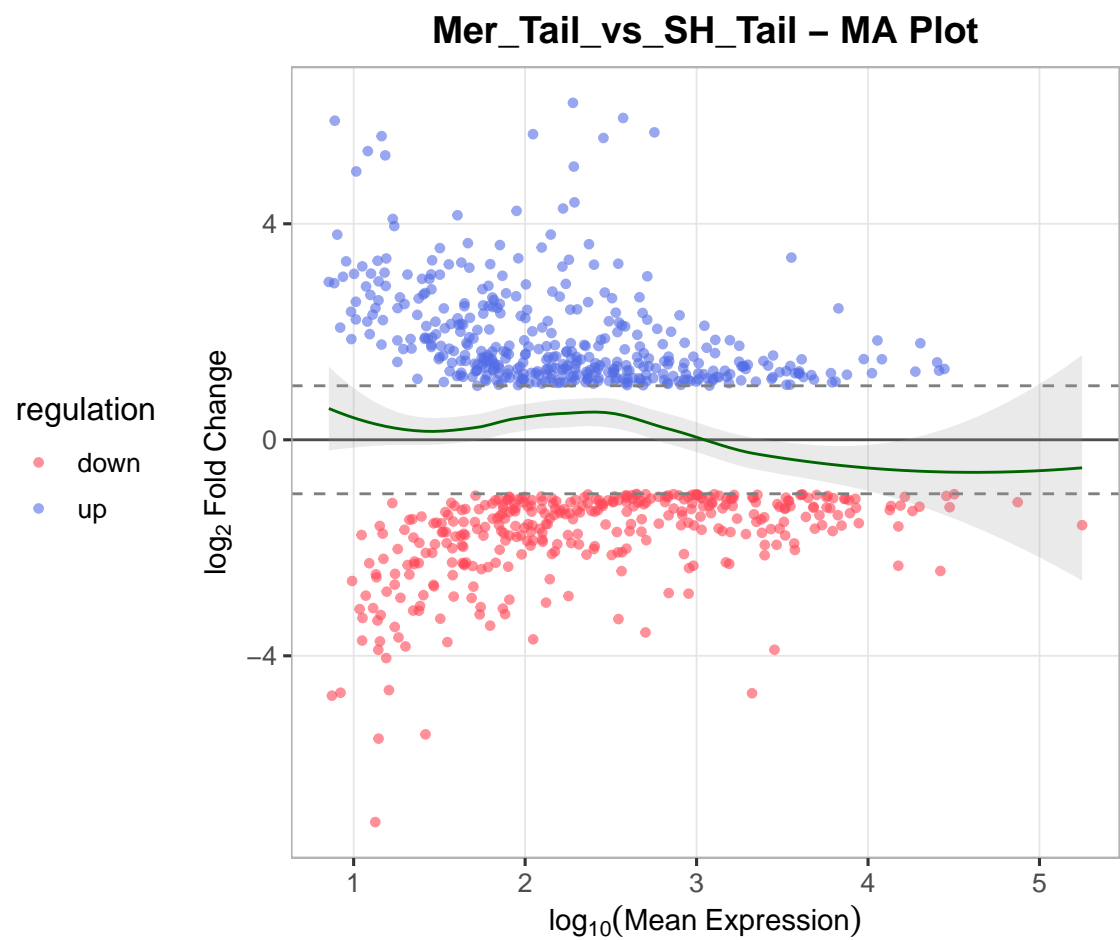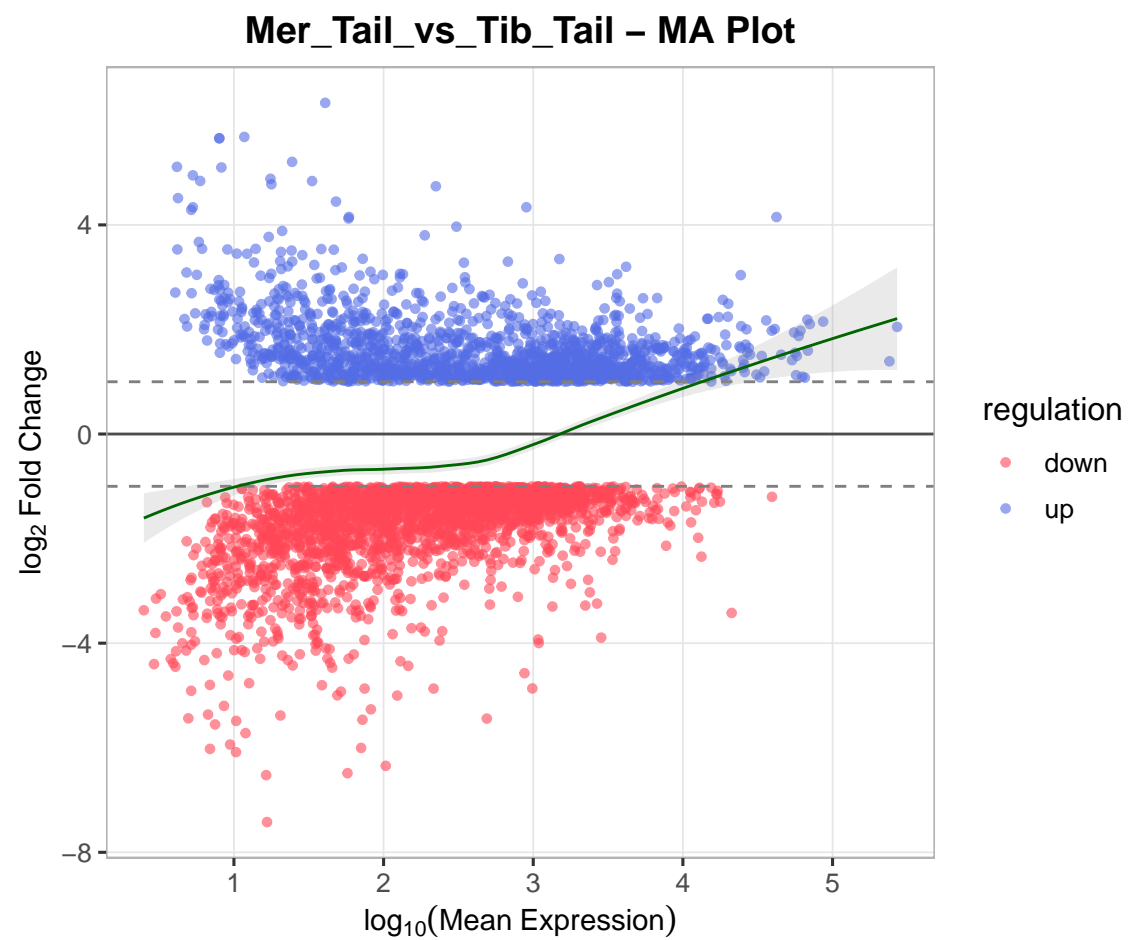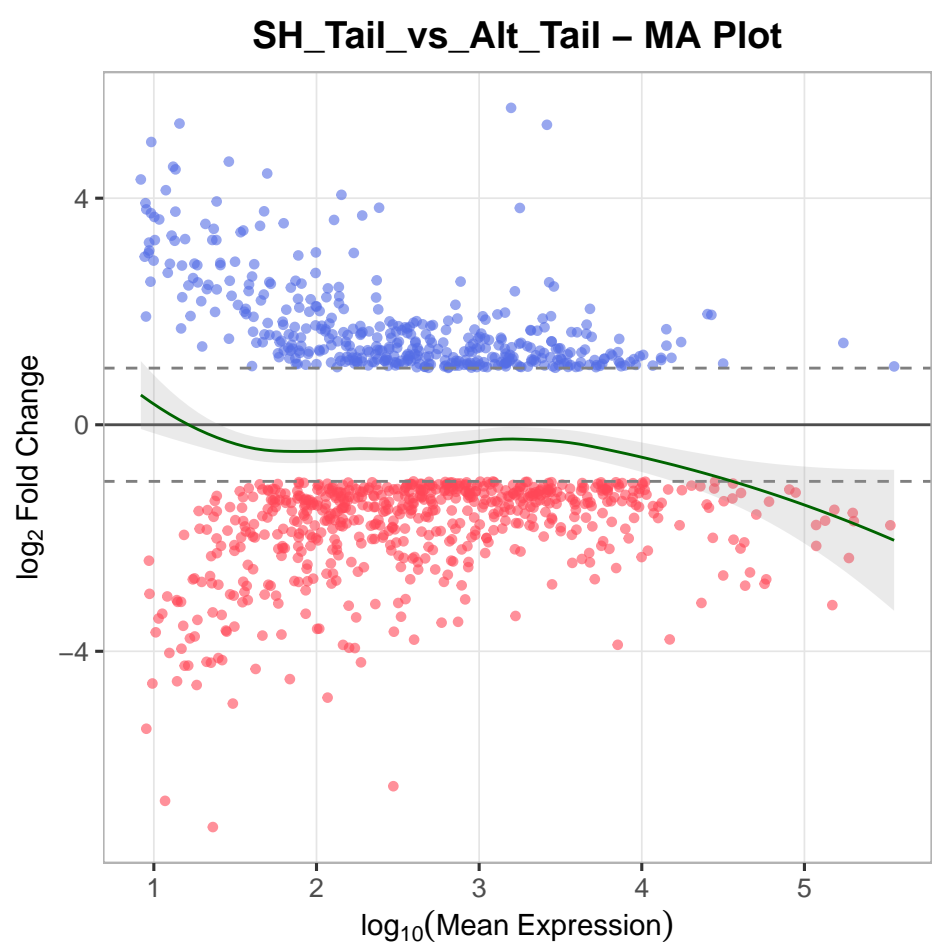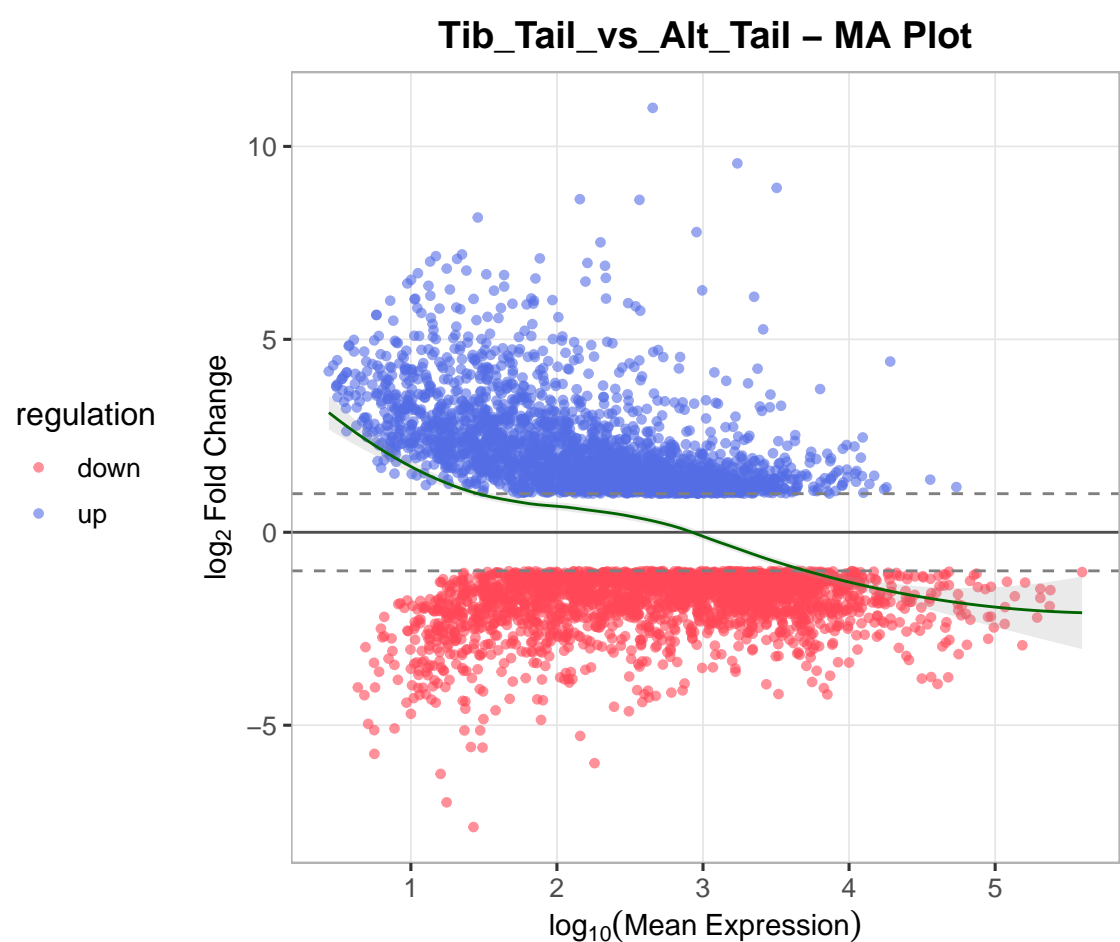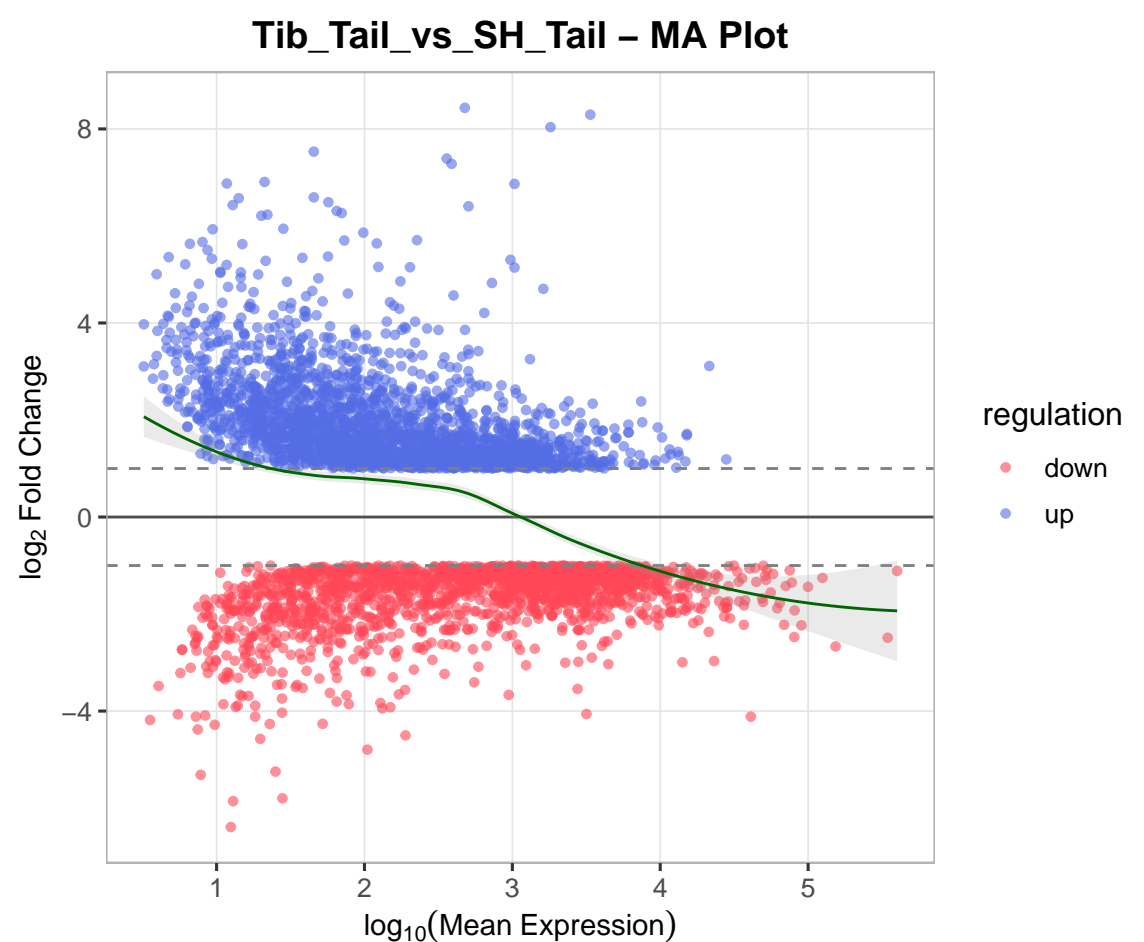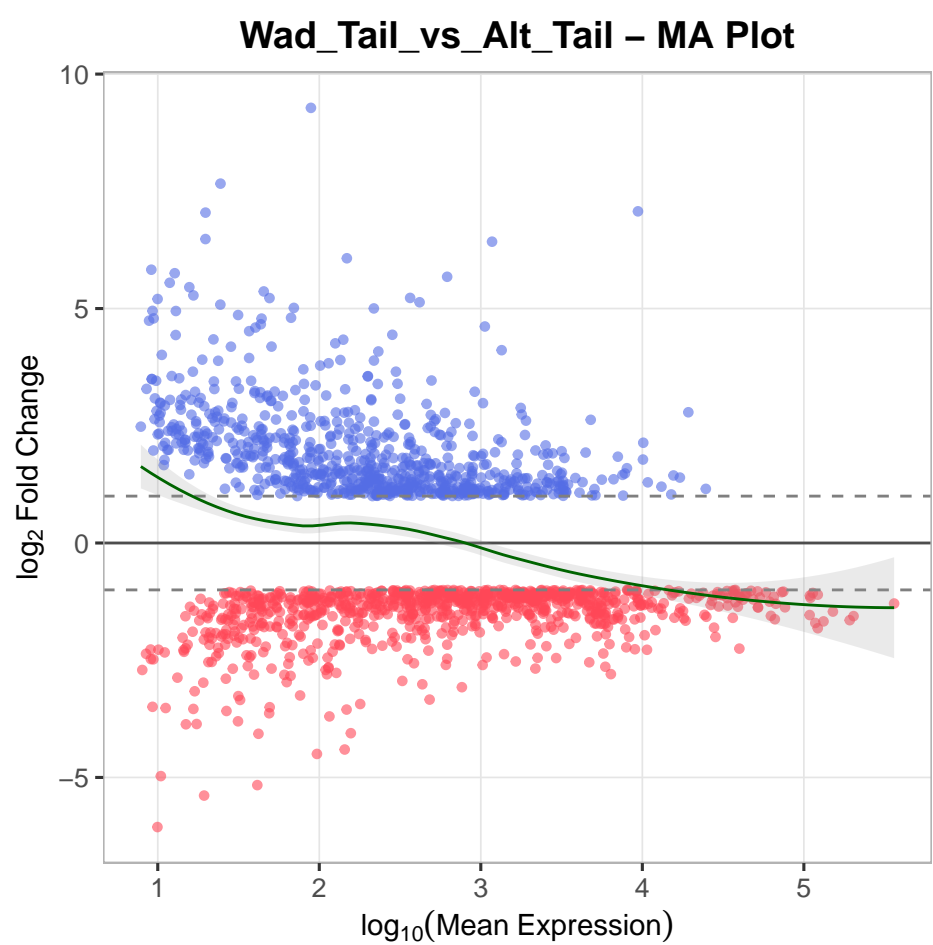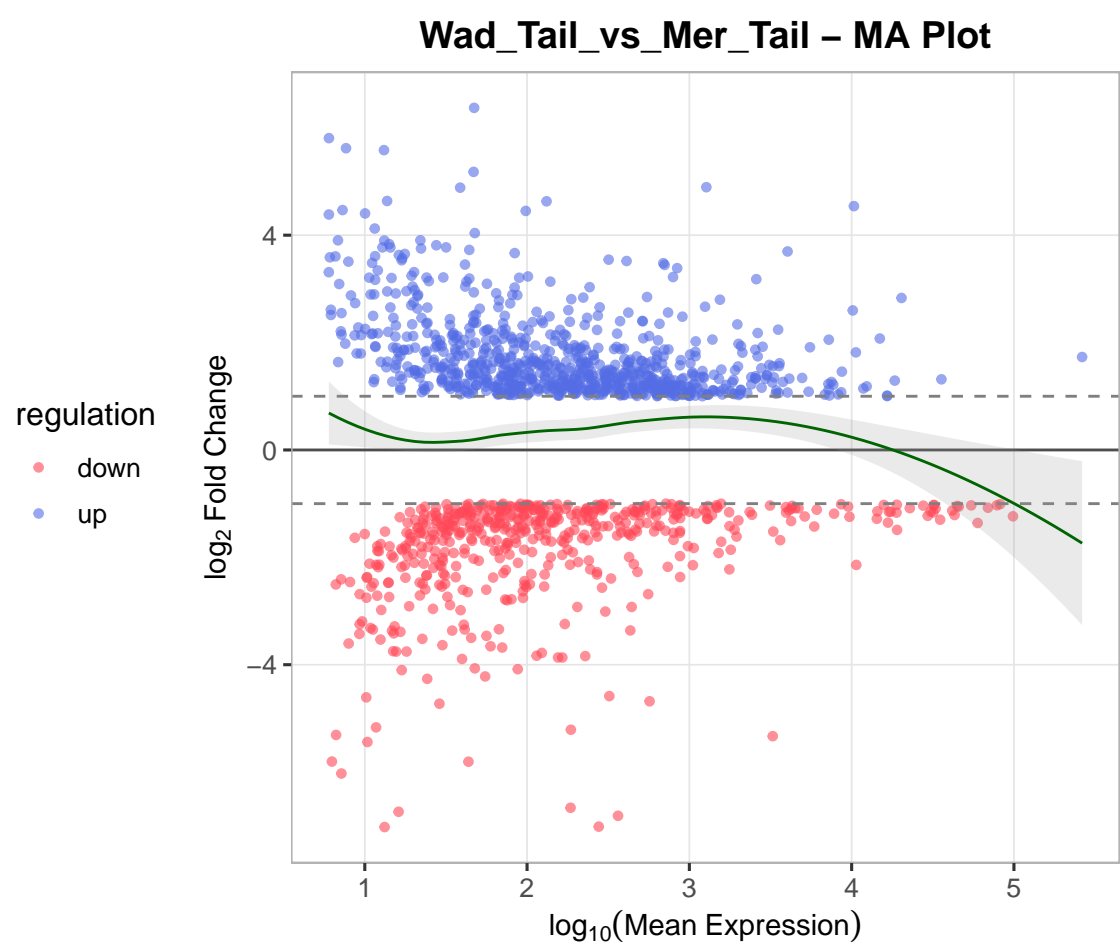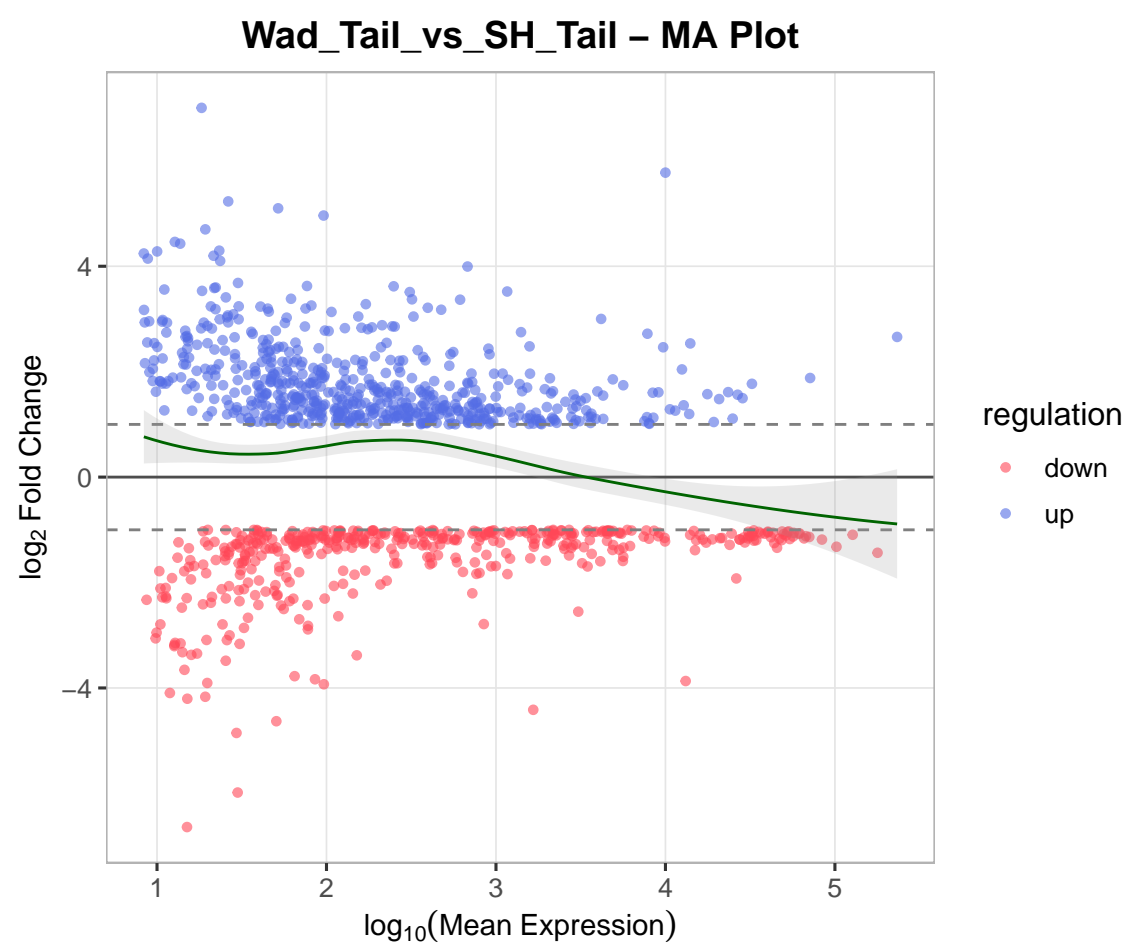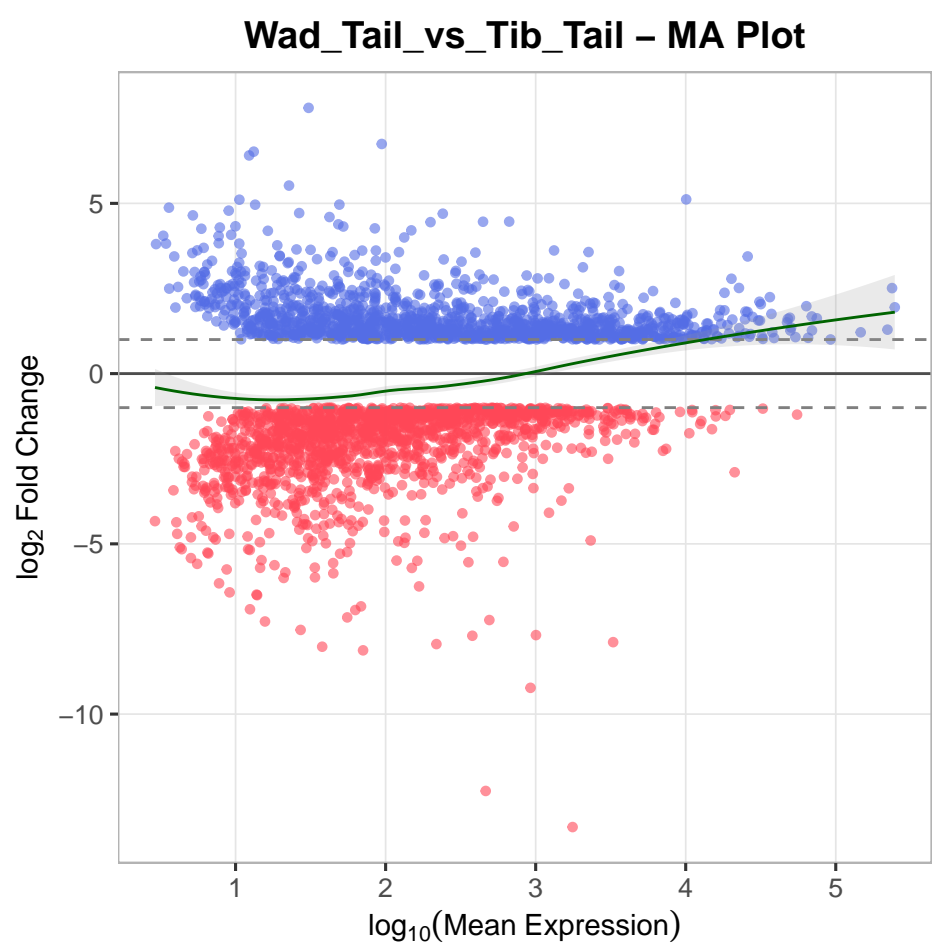

Supplement: Supplementary file 1 [file genes-17-00093-s001.zip › Supplementary Figures/Supplementary Figure 10.pdf]

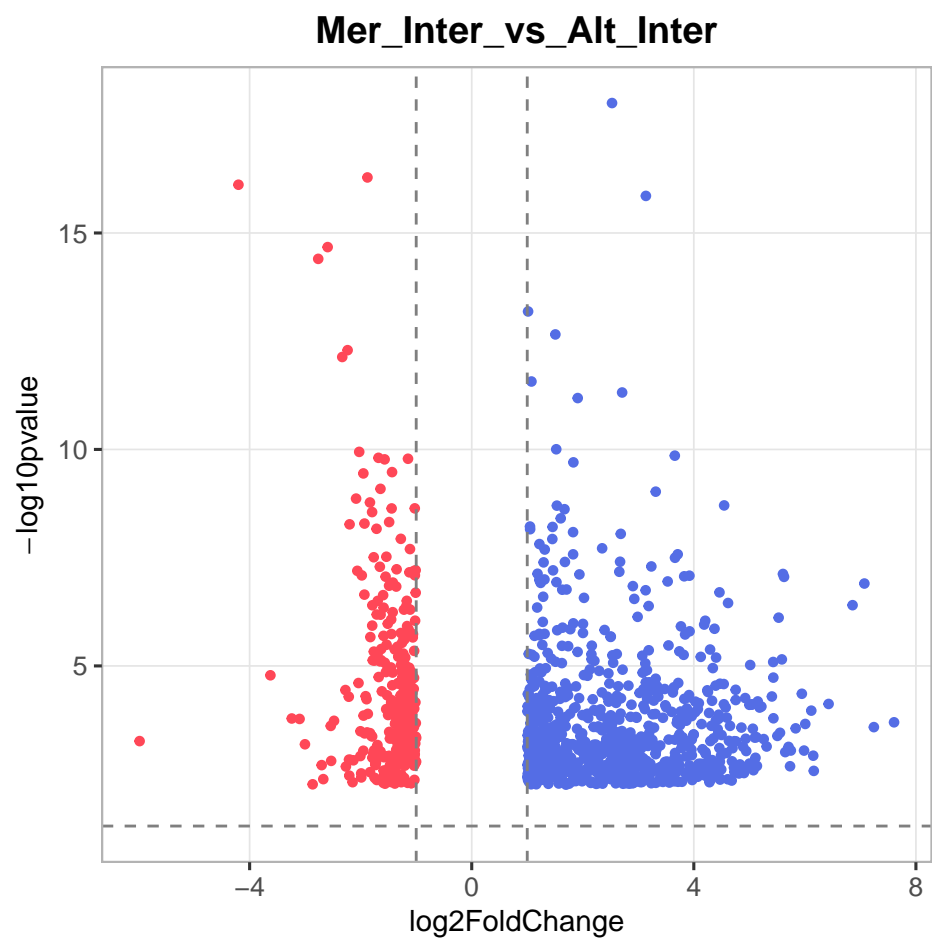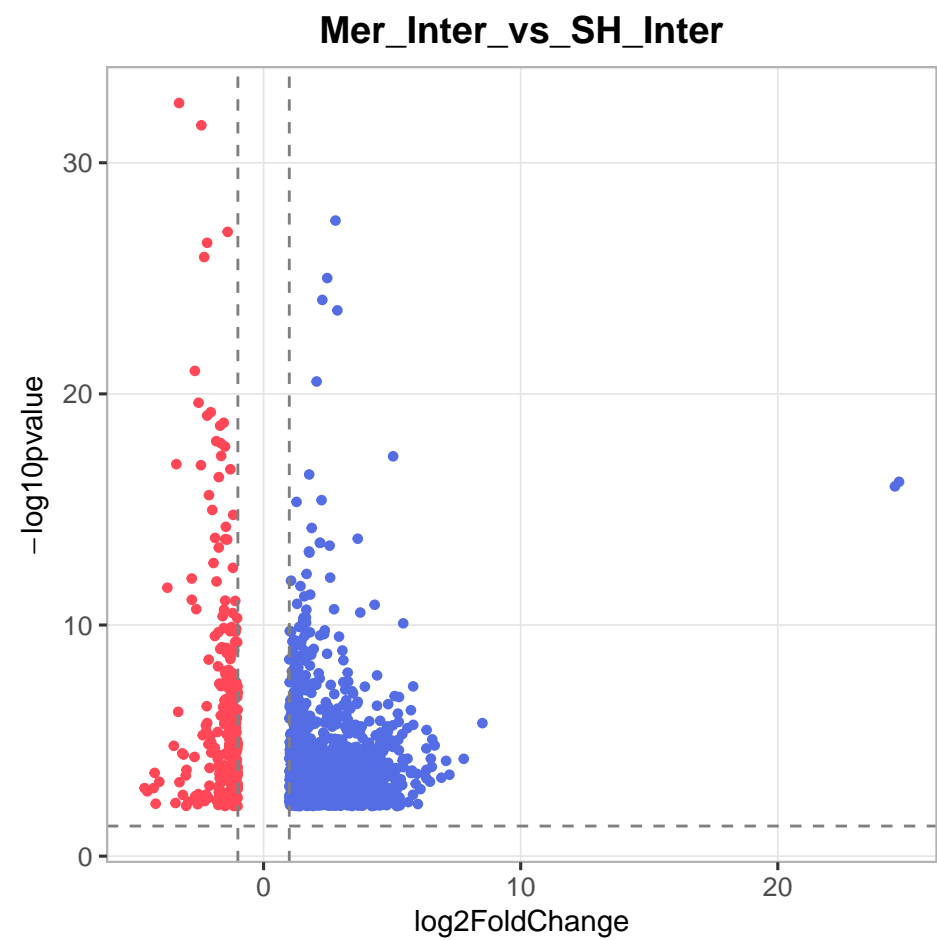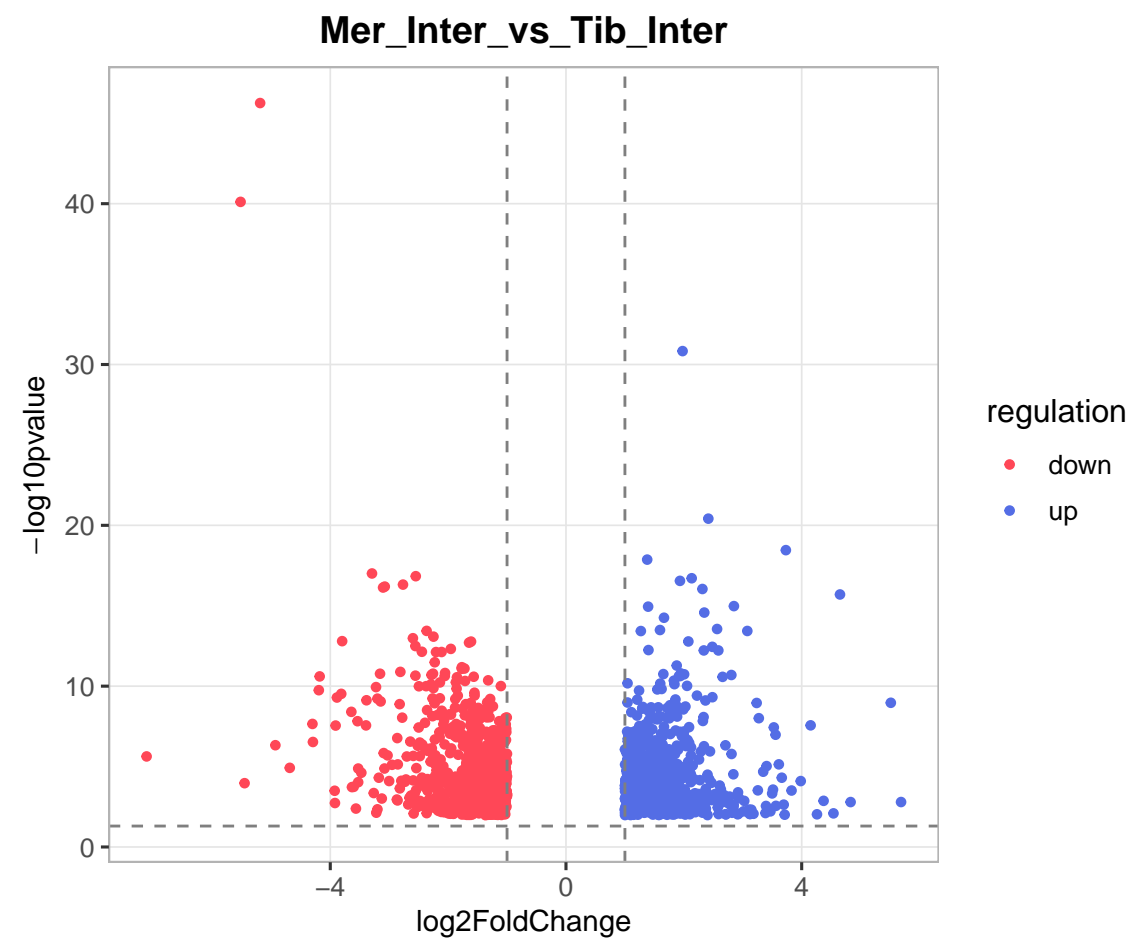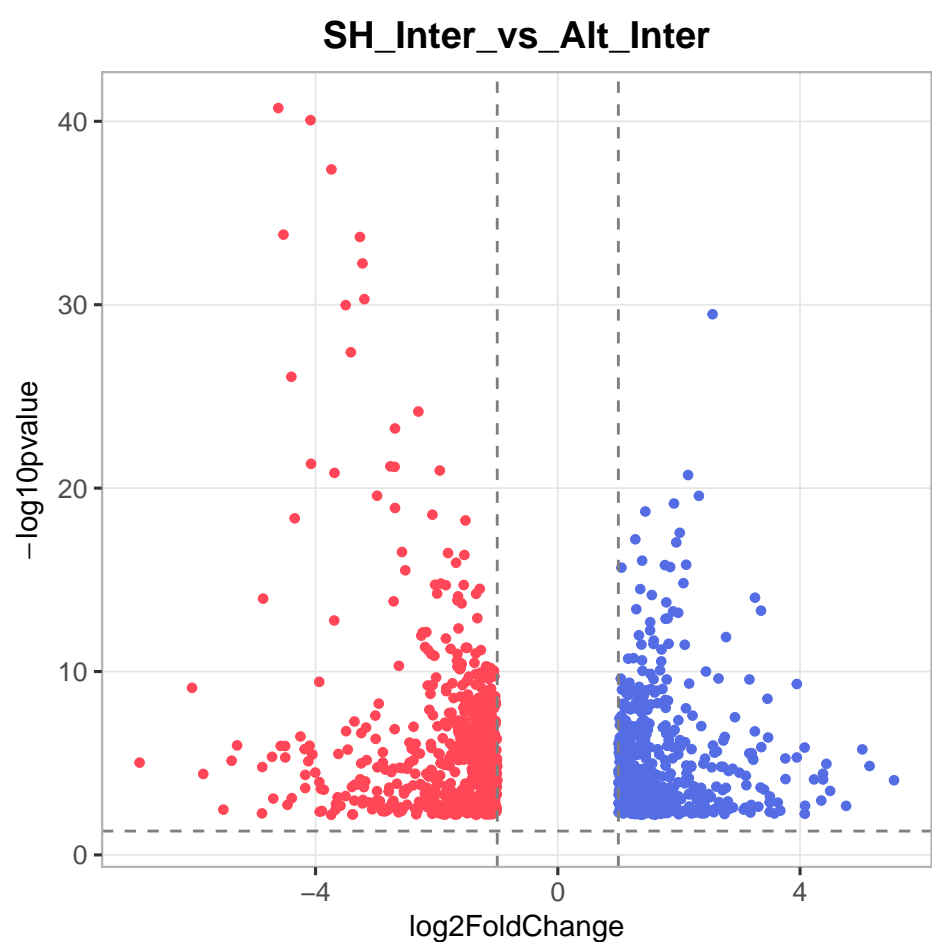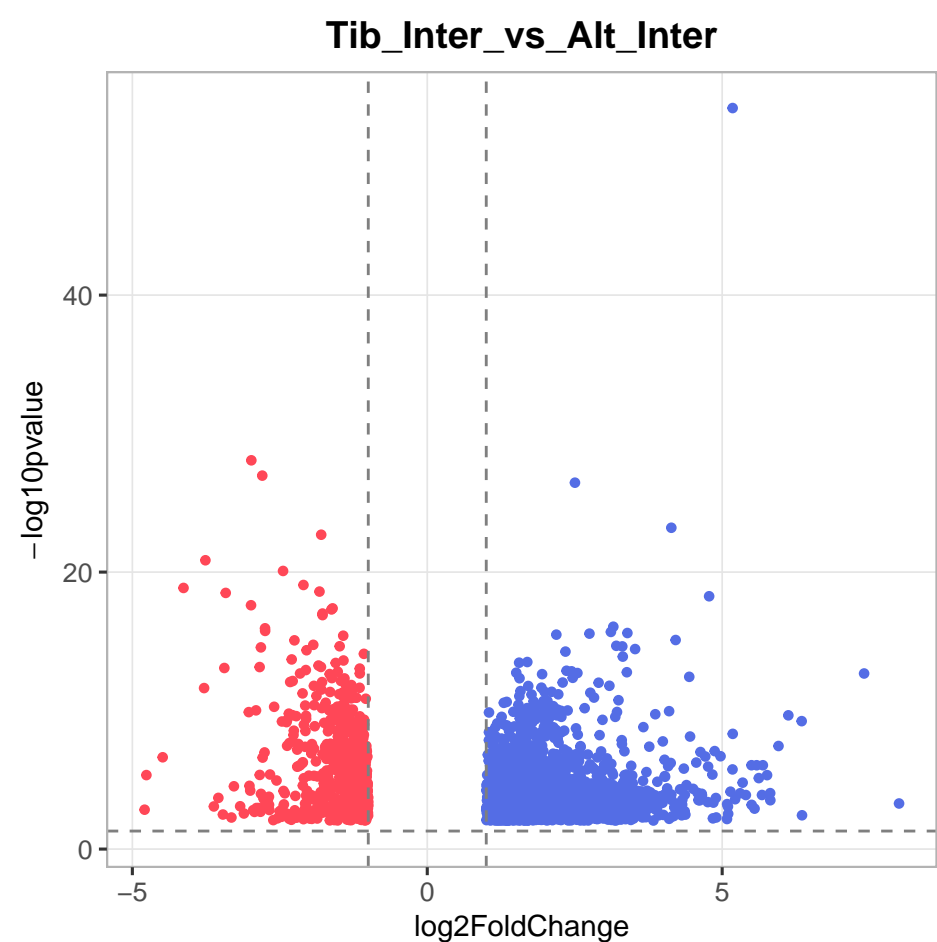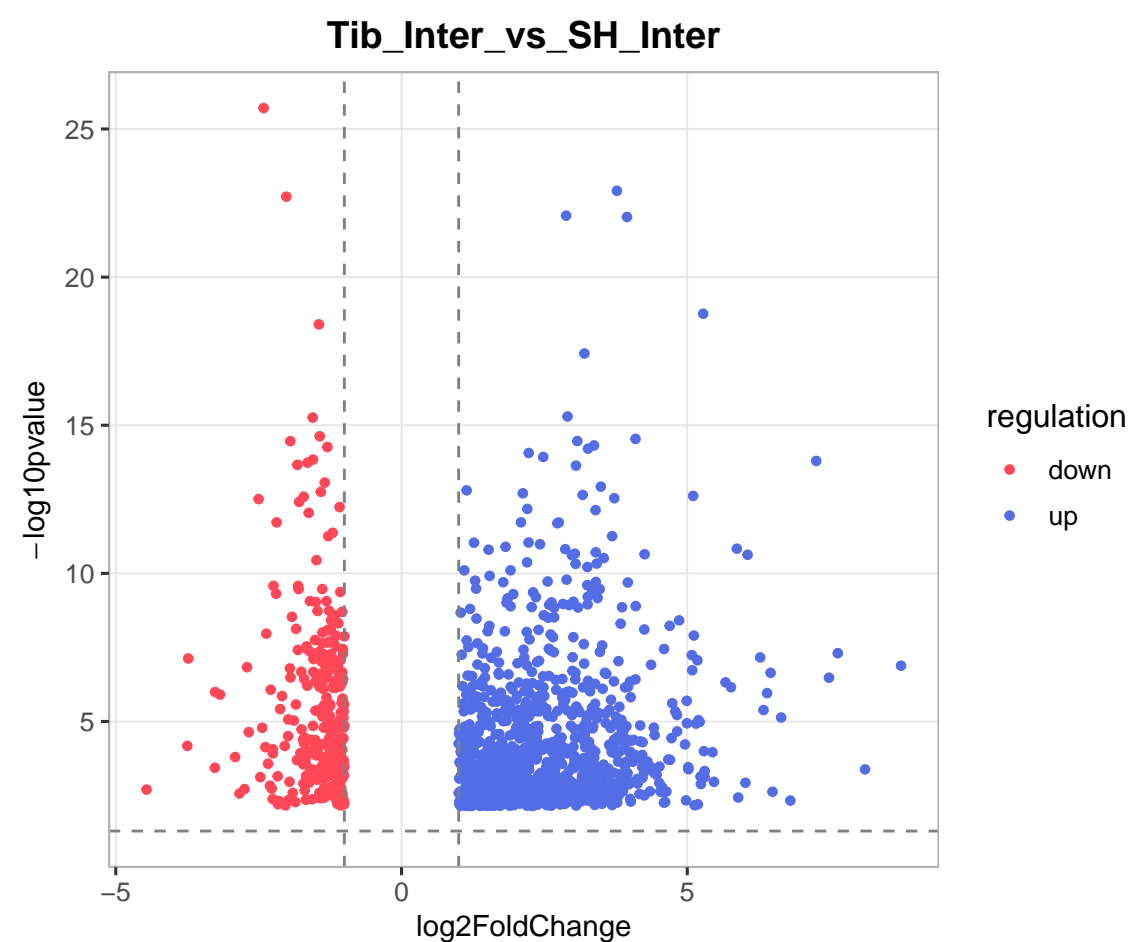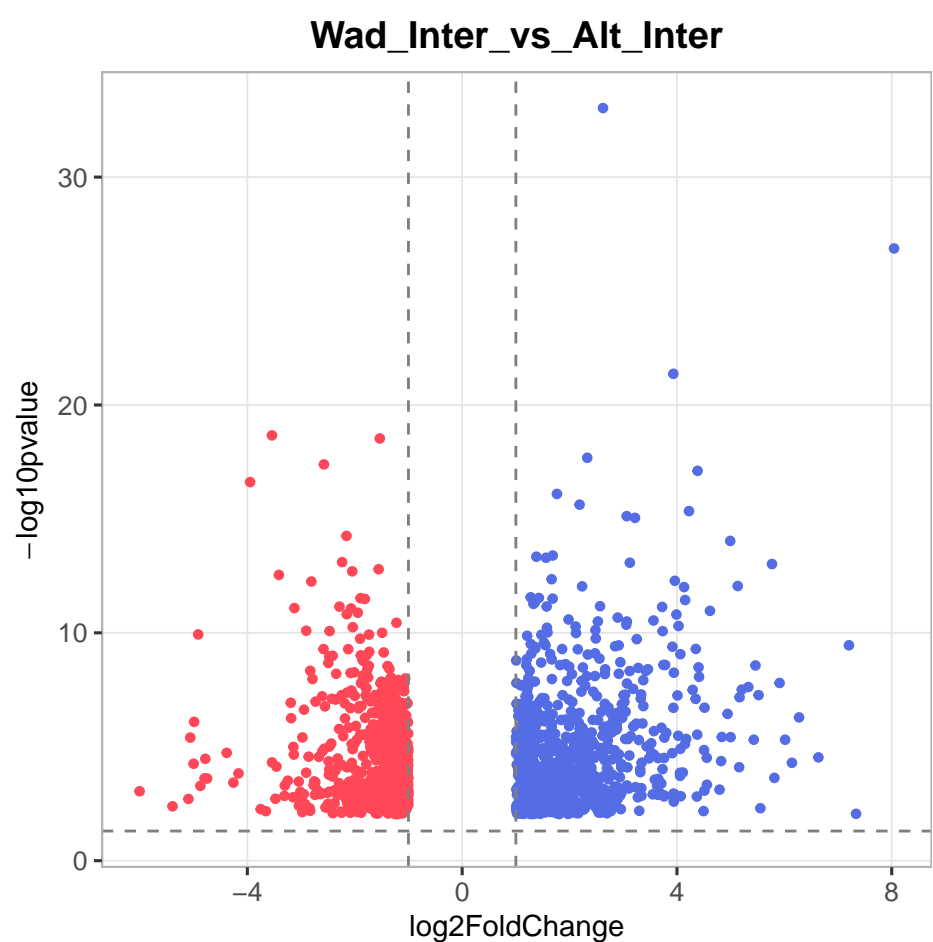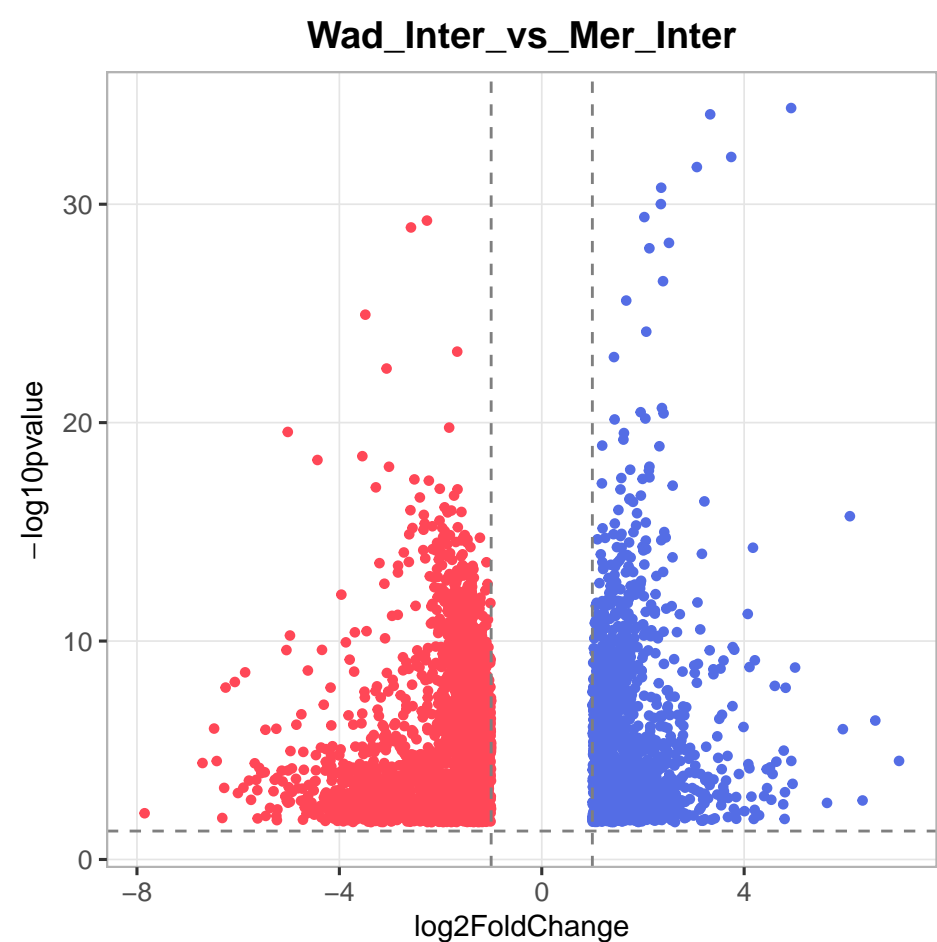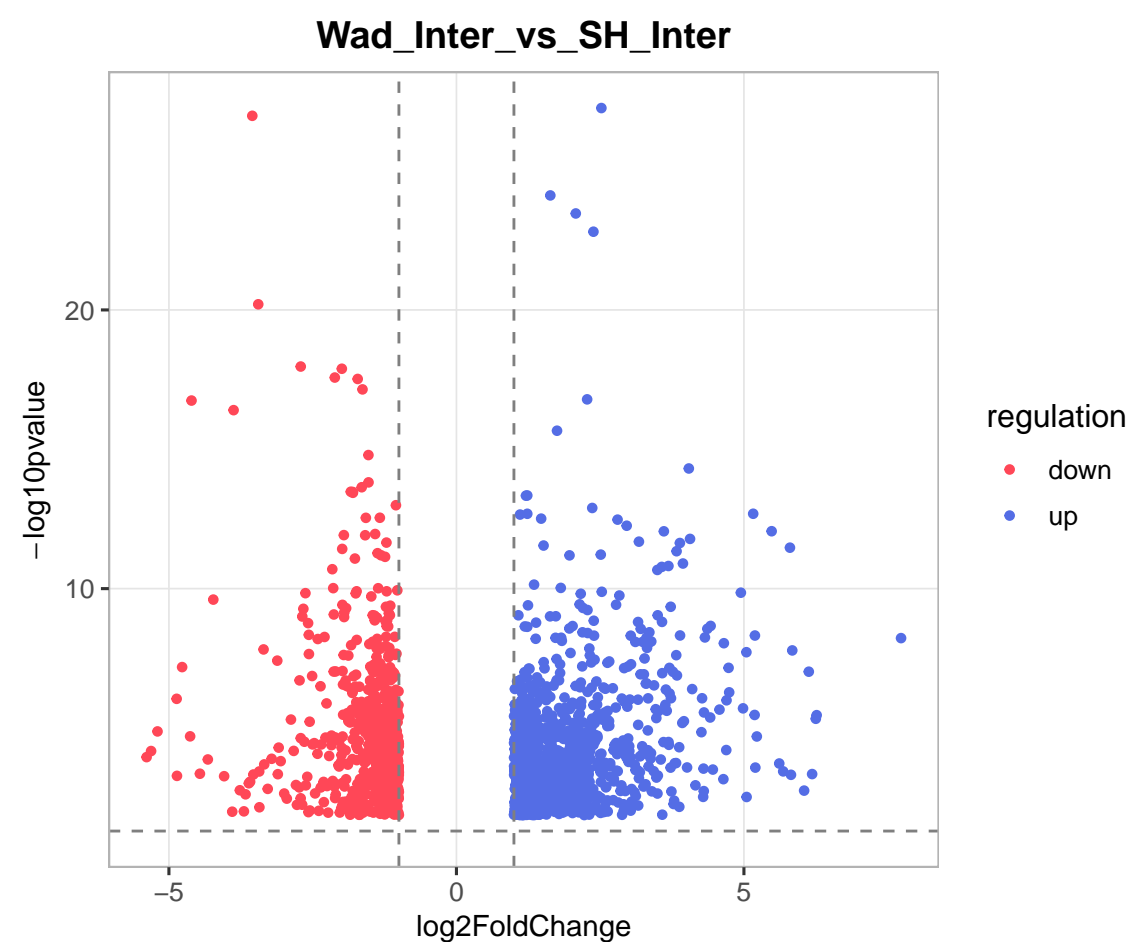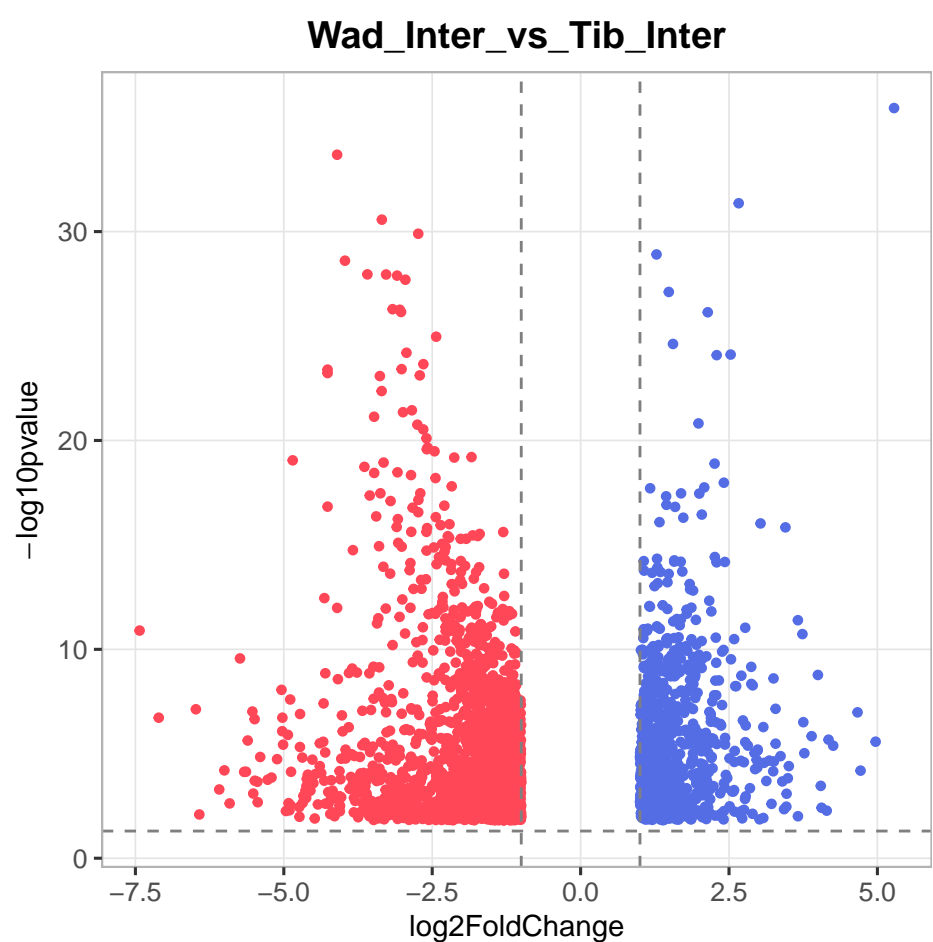

Supplement: Supplementary file 1 [file genes-17-00093-s001.zip › Supplementary Figures/Supplementary Figure 2.pdf]

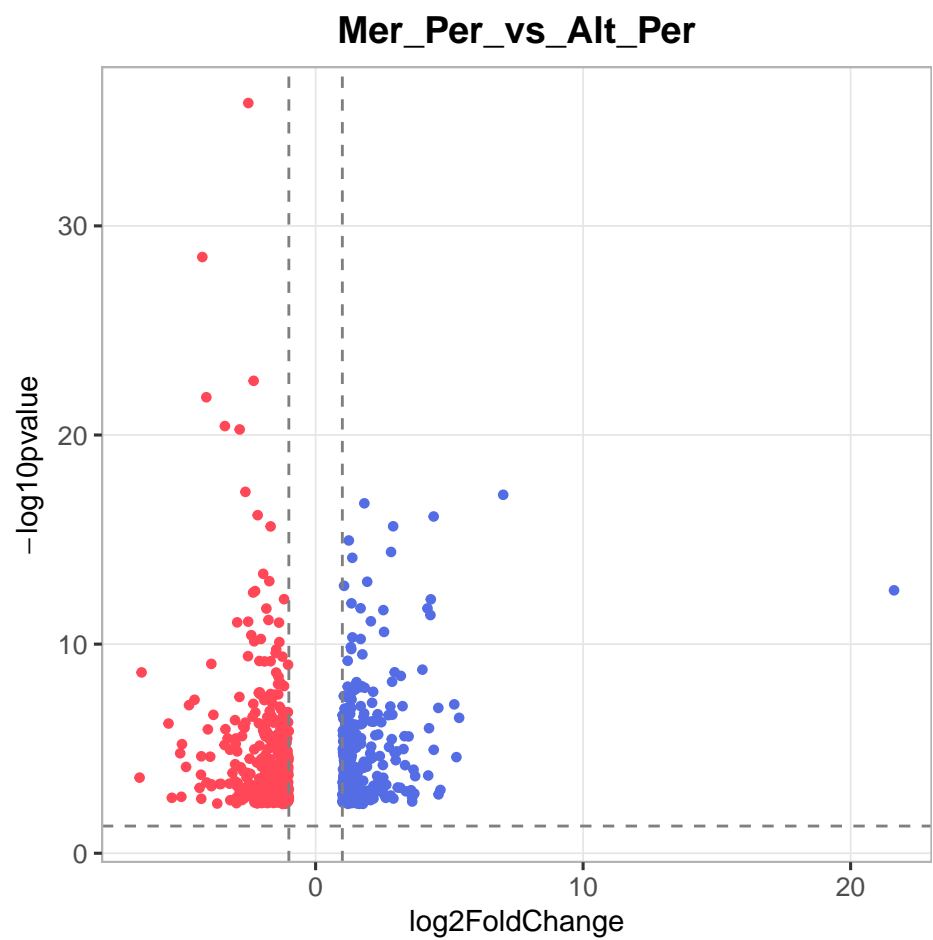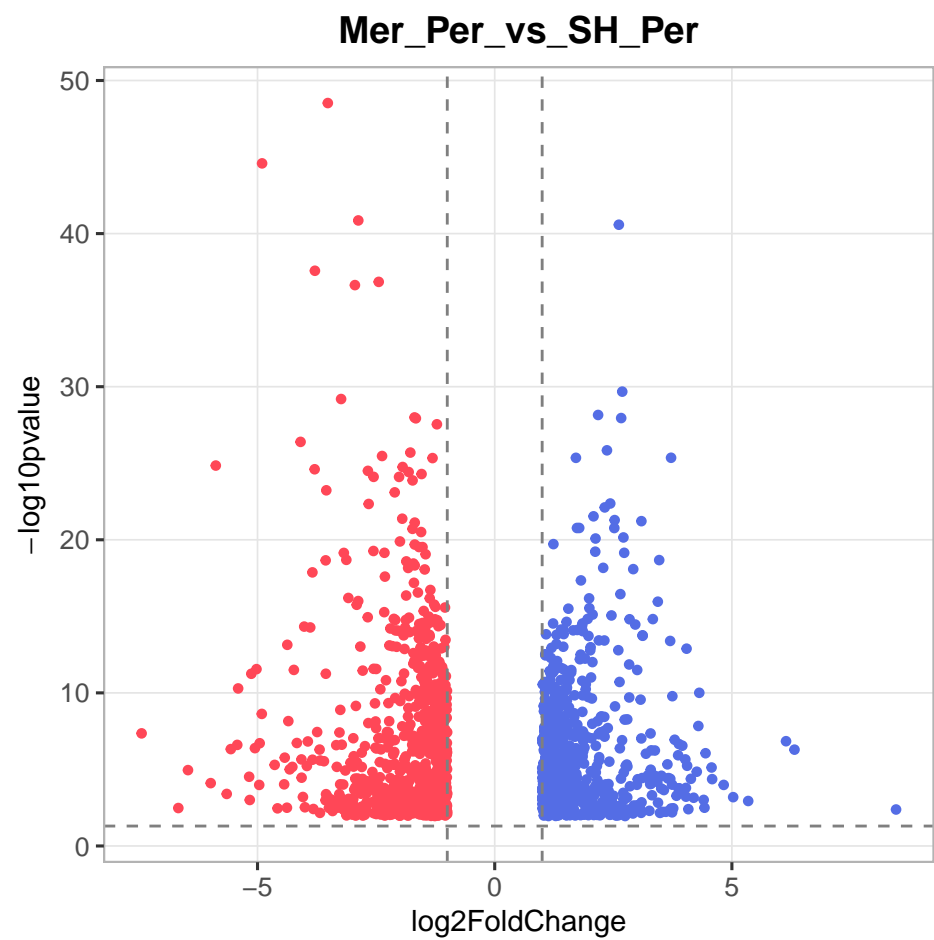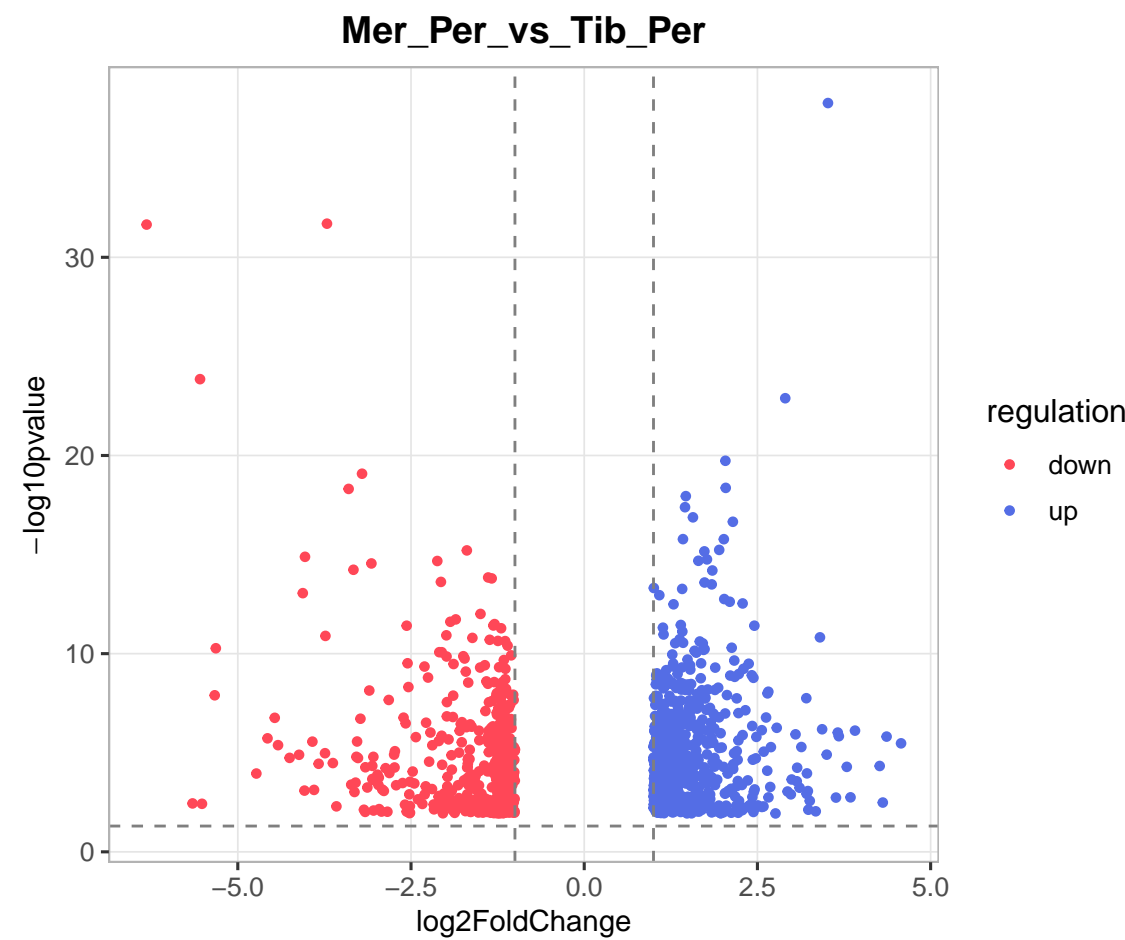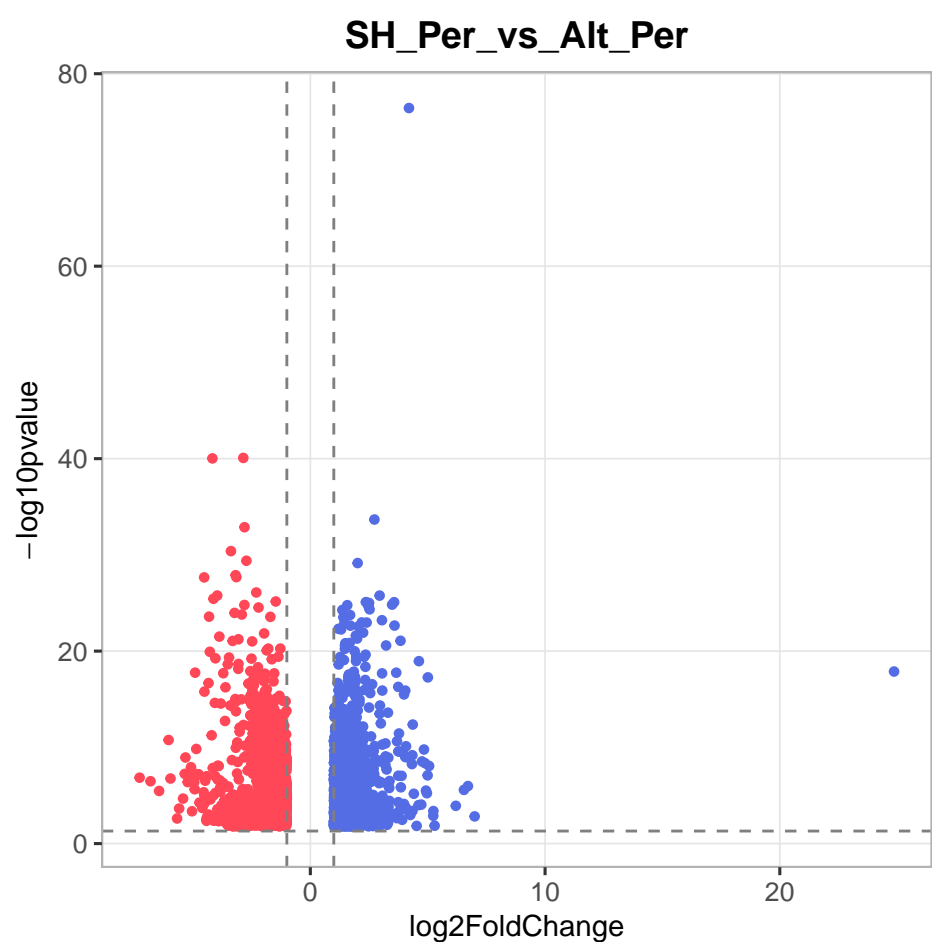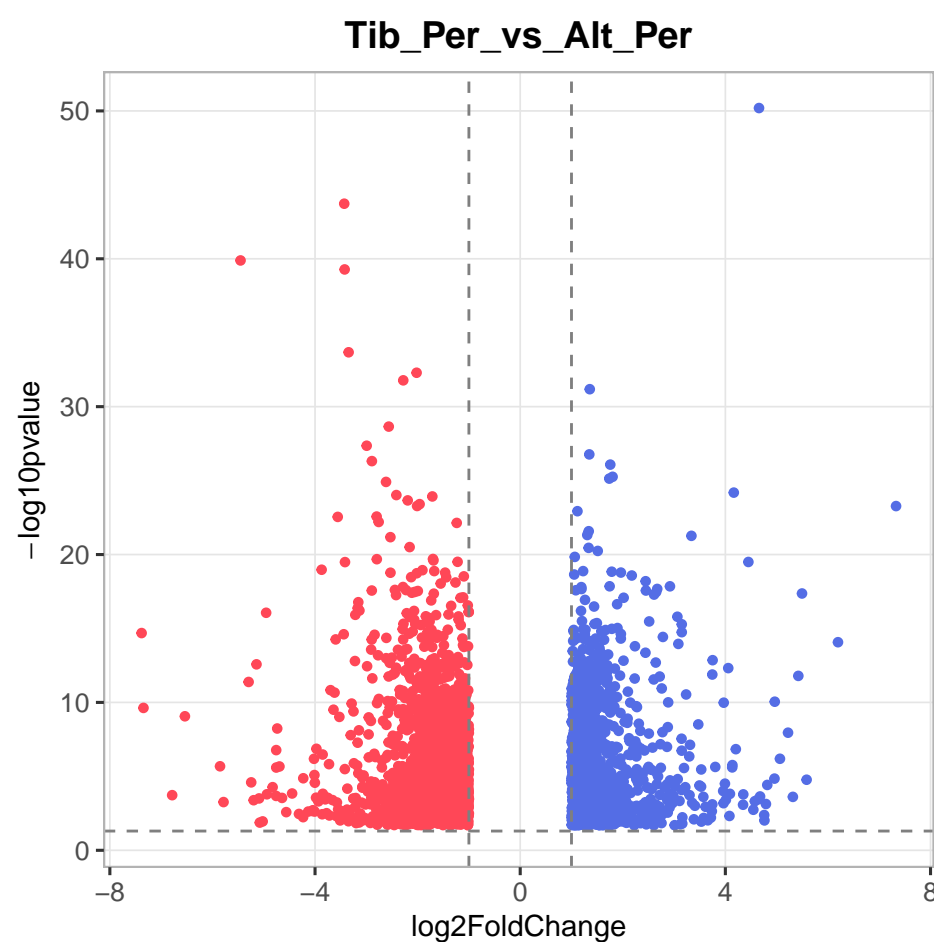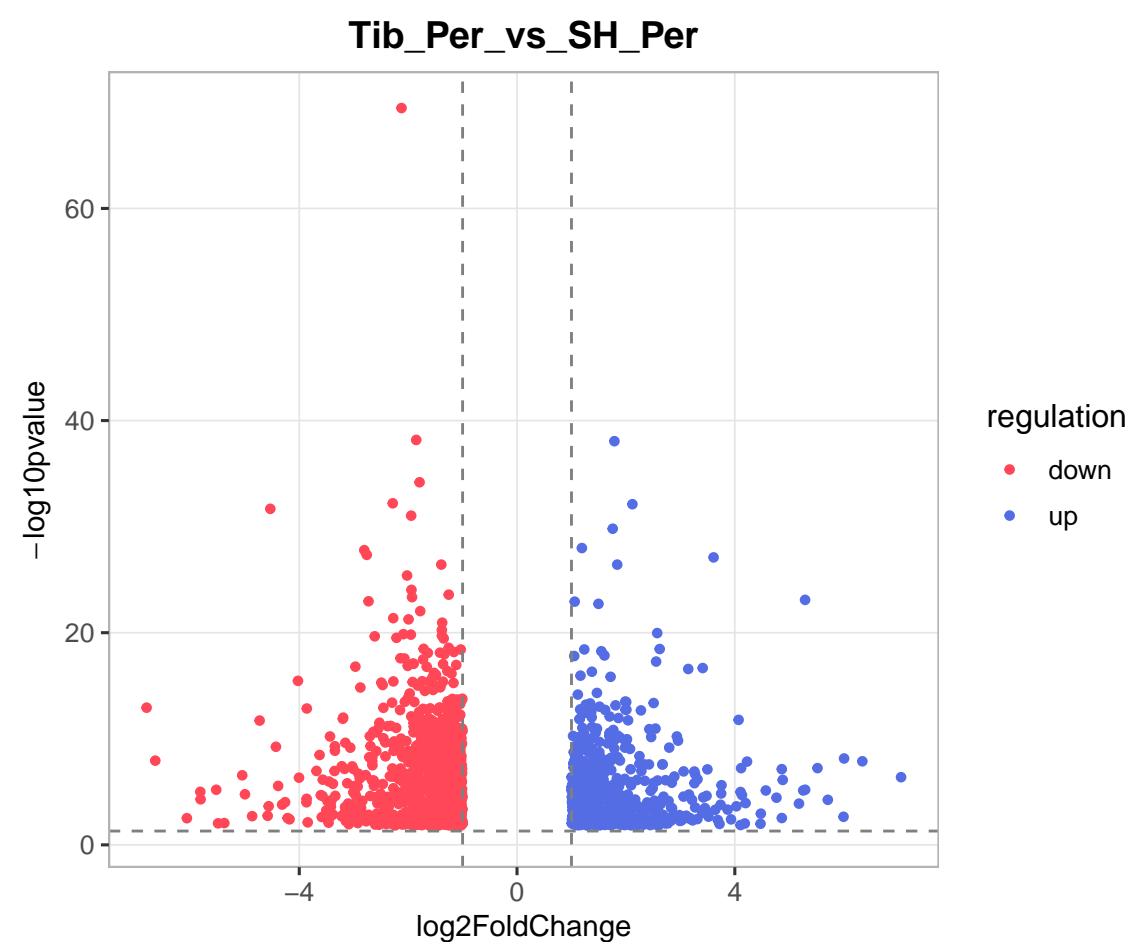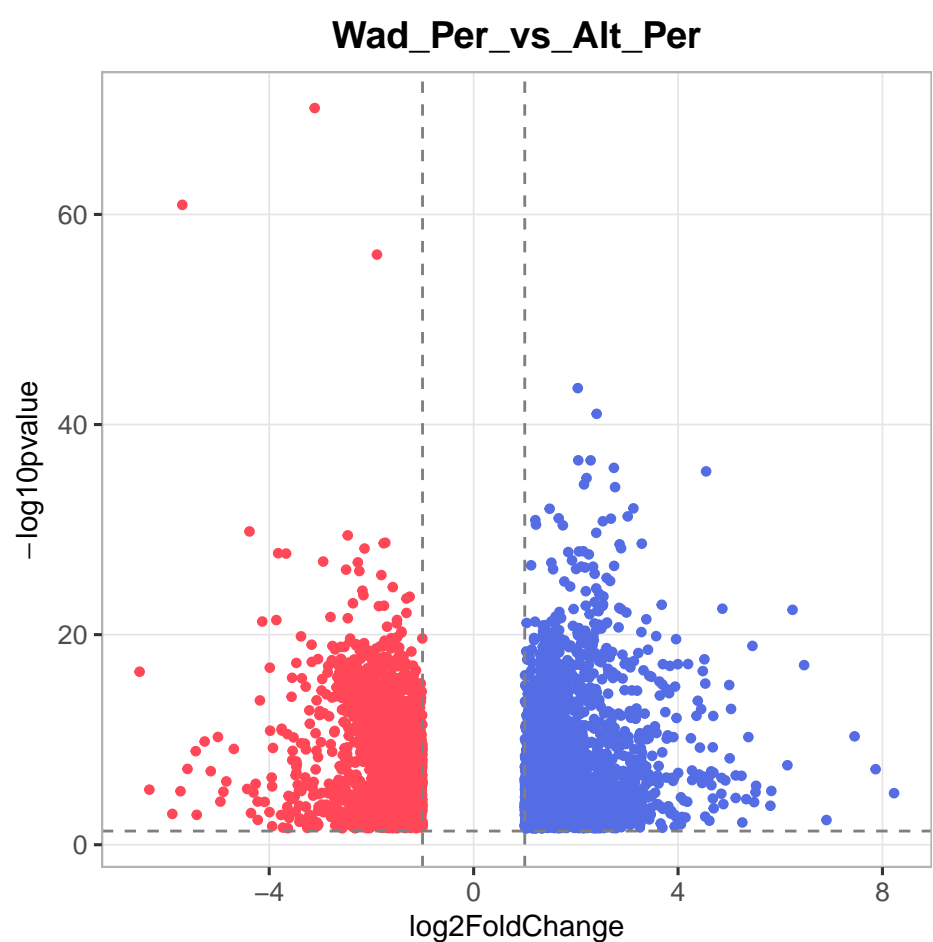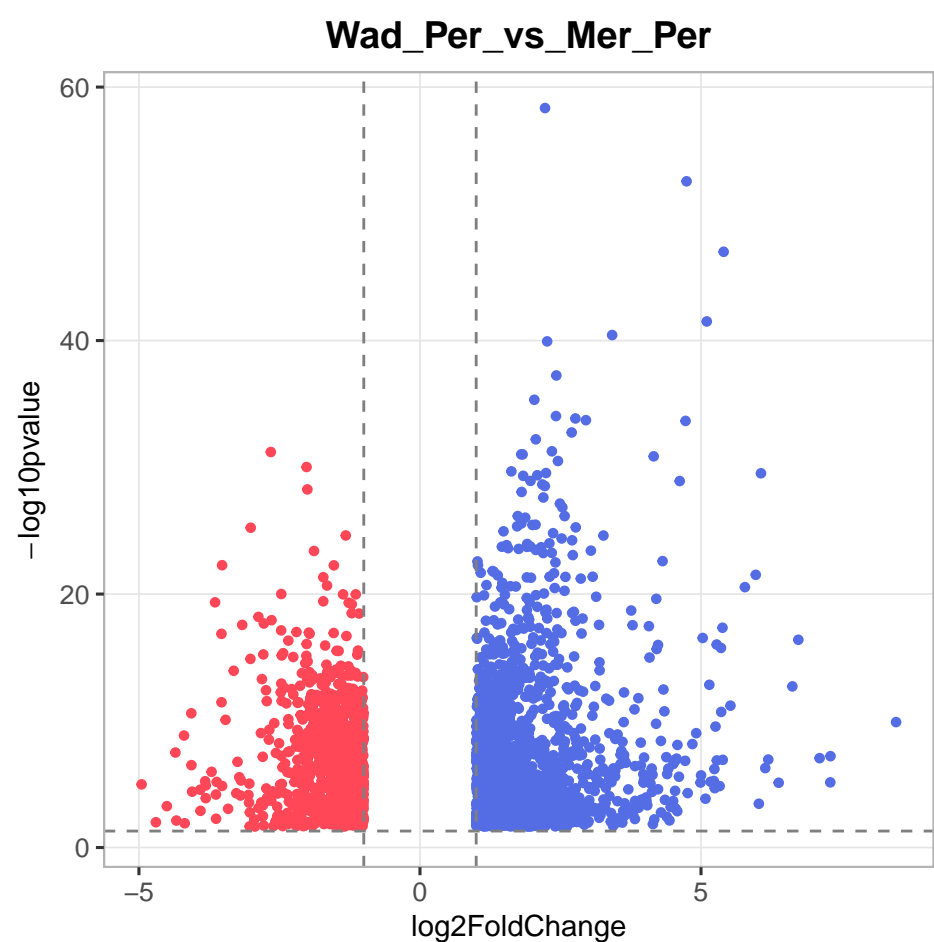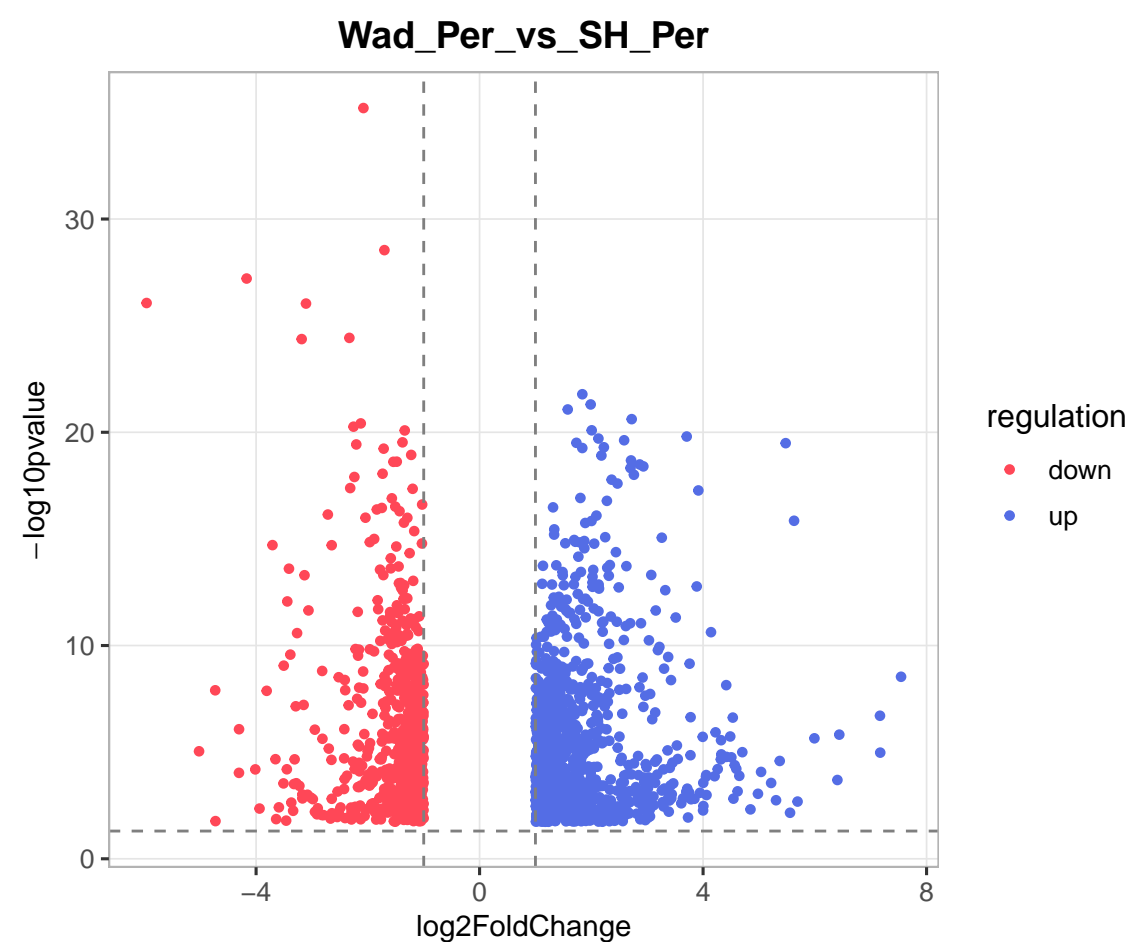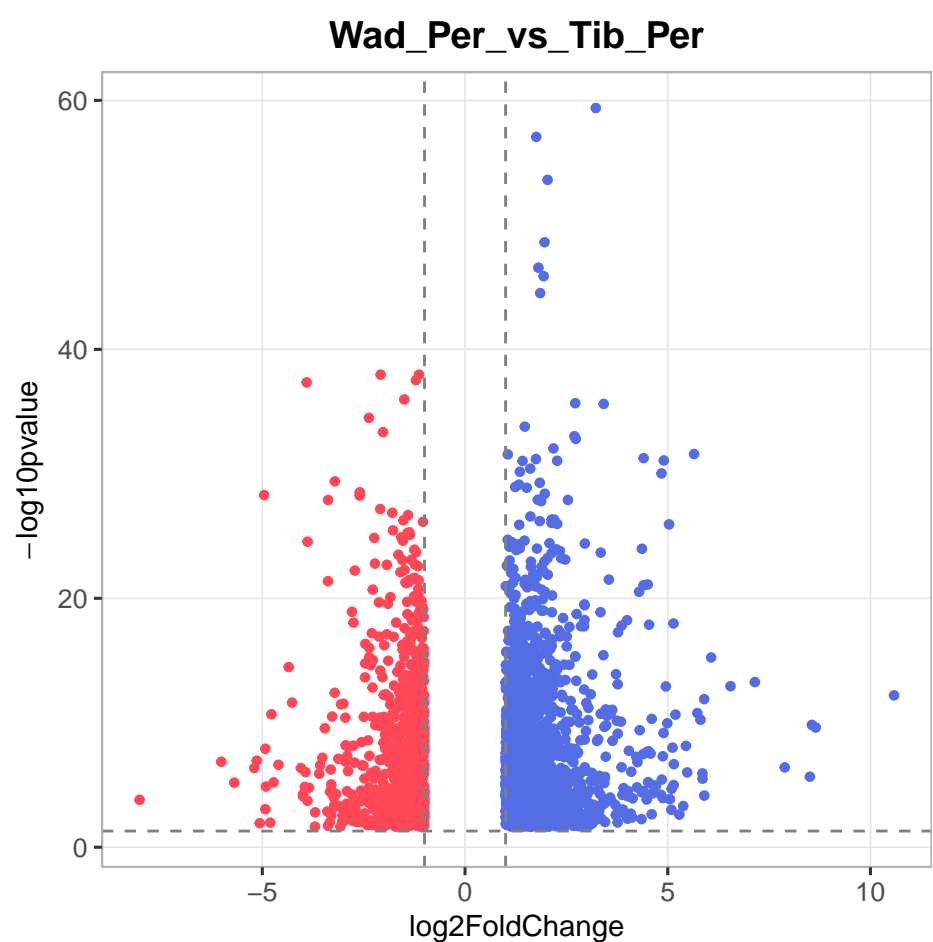

Supplement: Supplementary file 1 [file genes-17-00093-s001.zip › Supplementary Figures/Supplementary Figure 3.pdf]

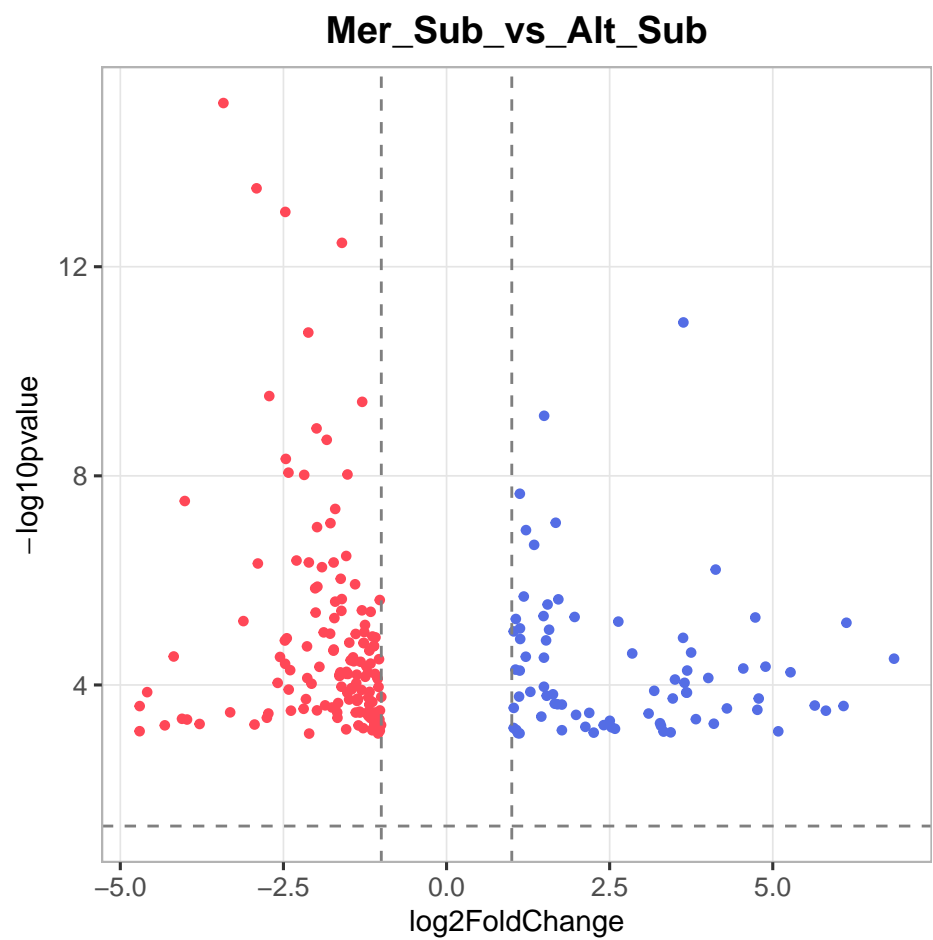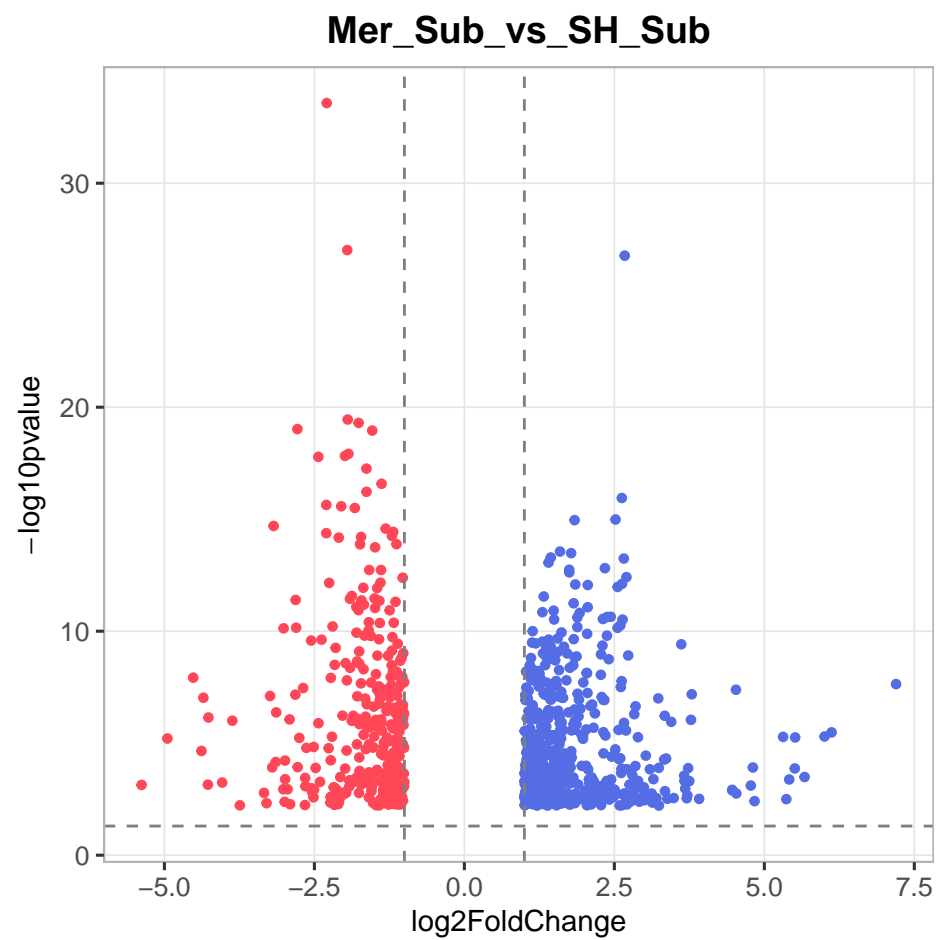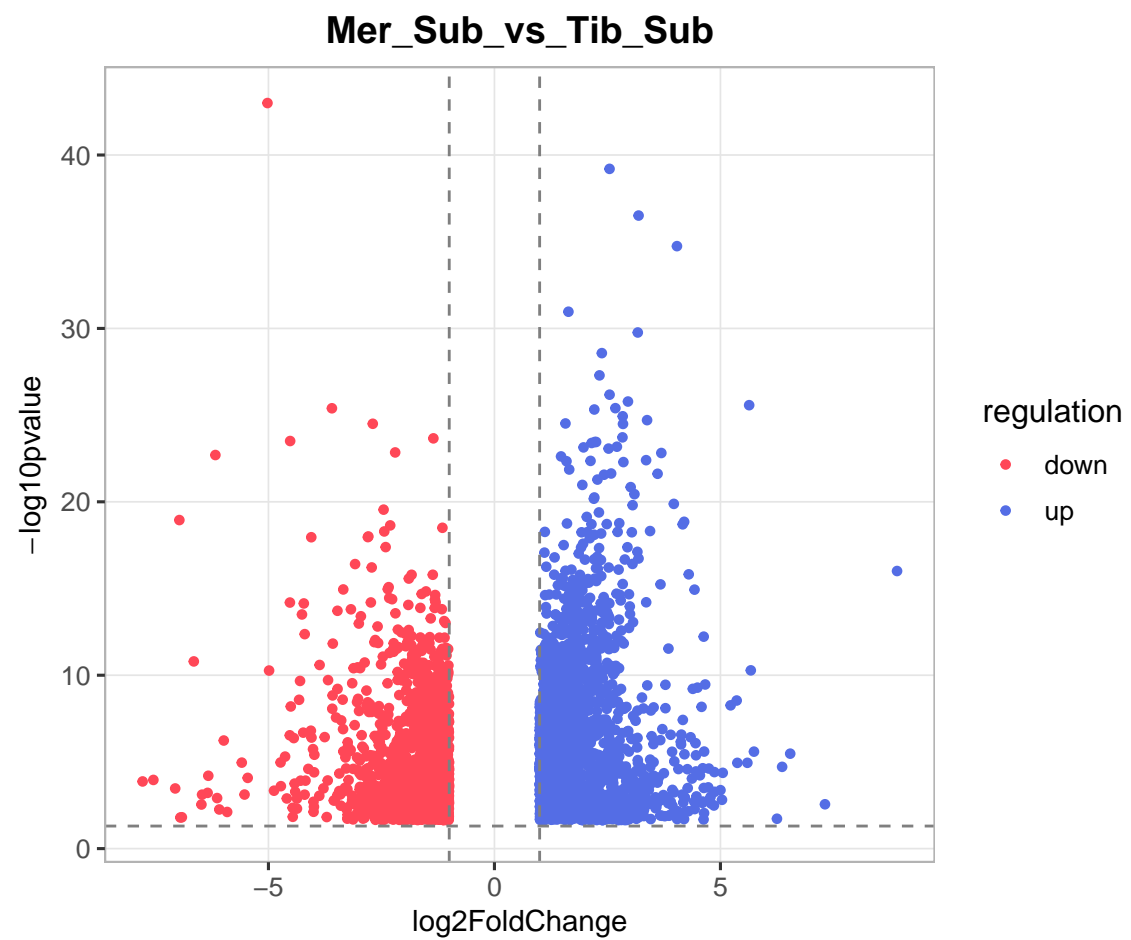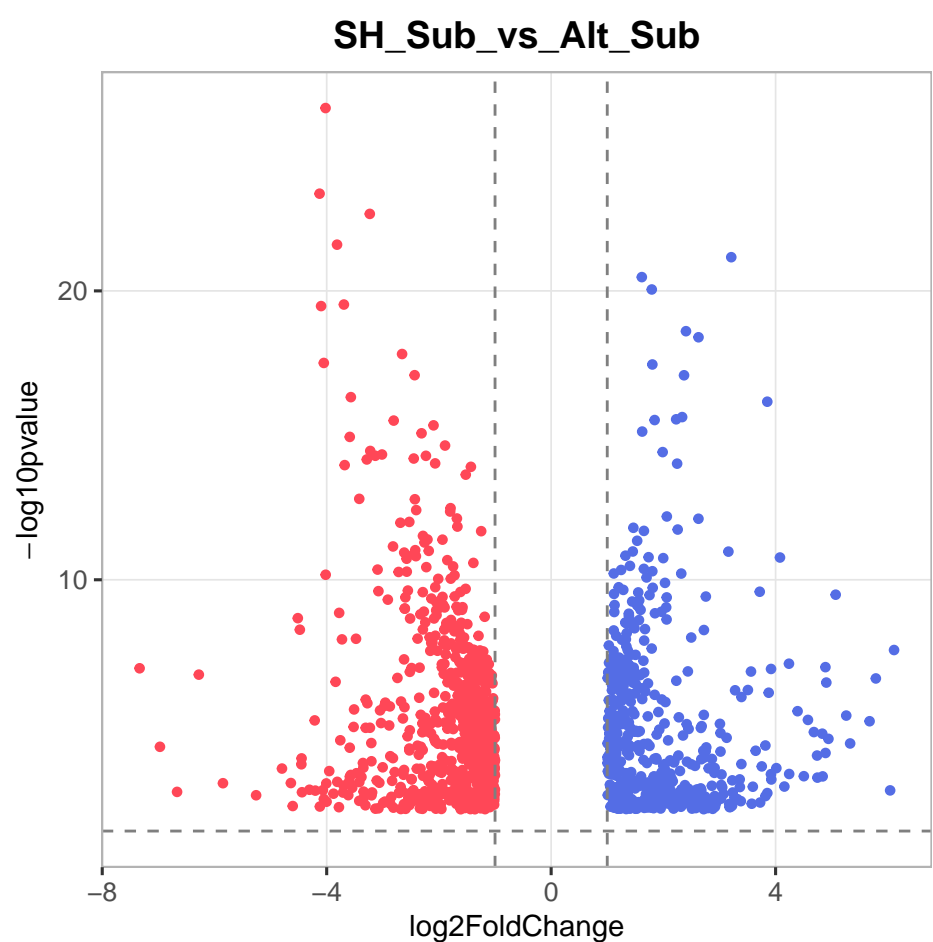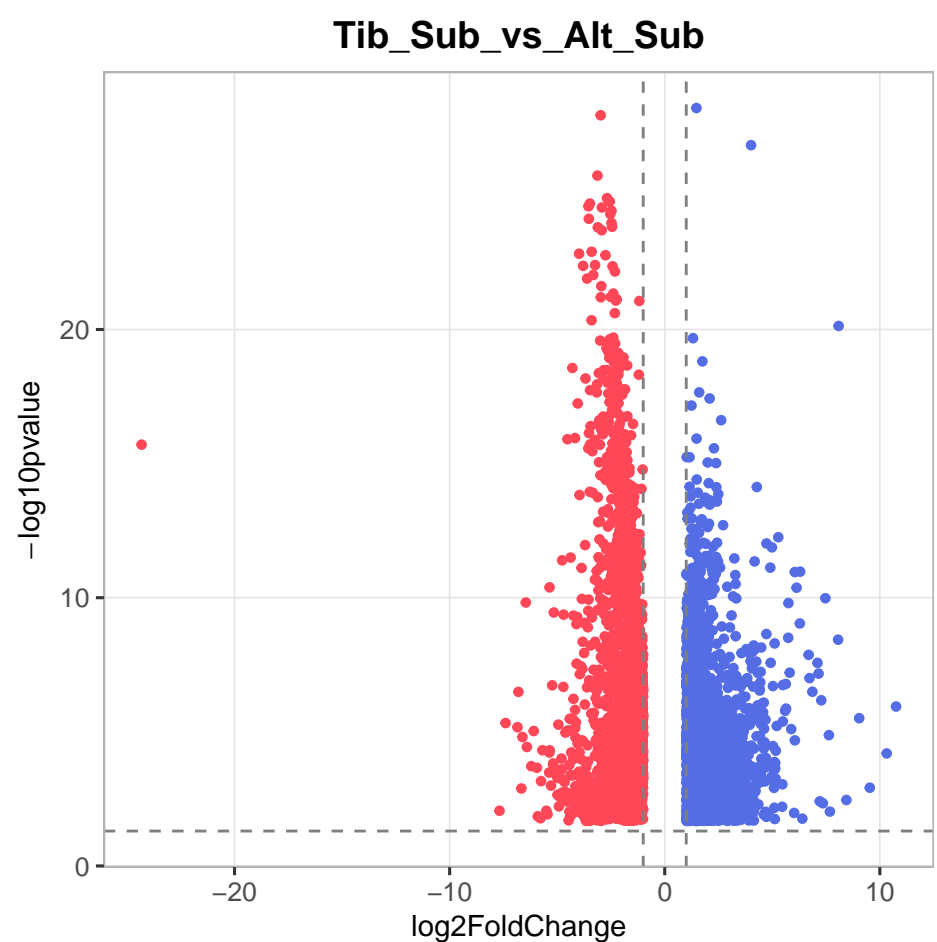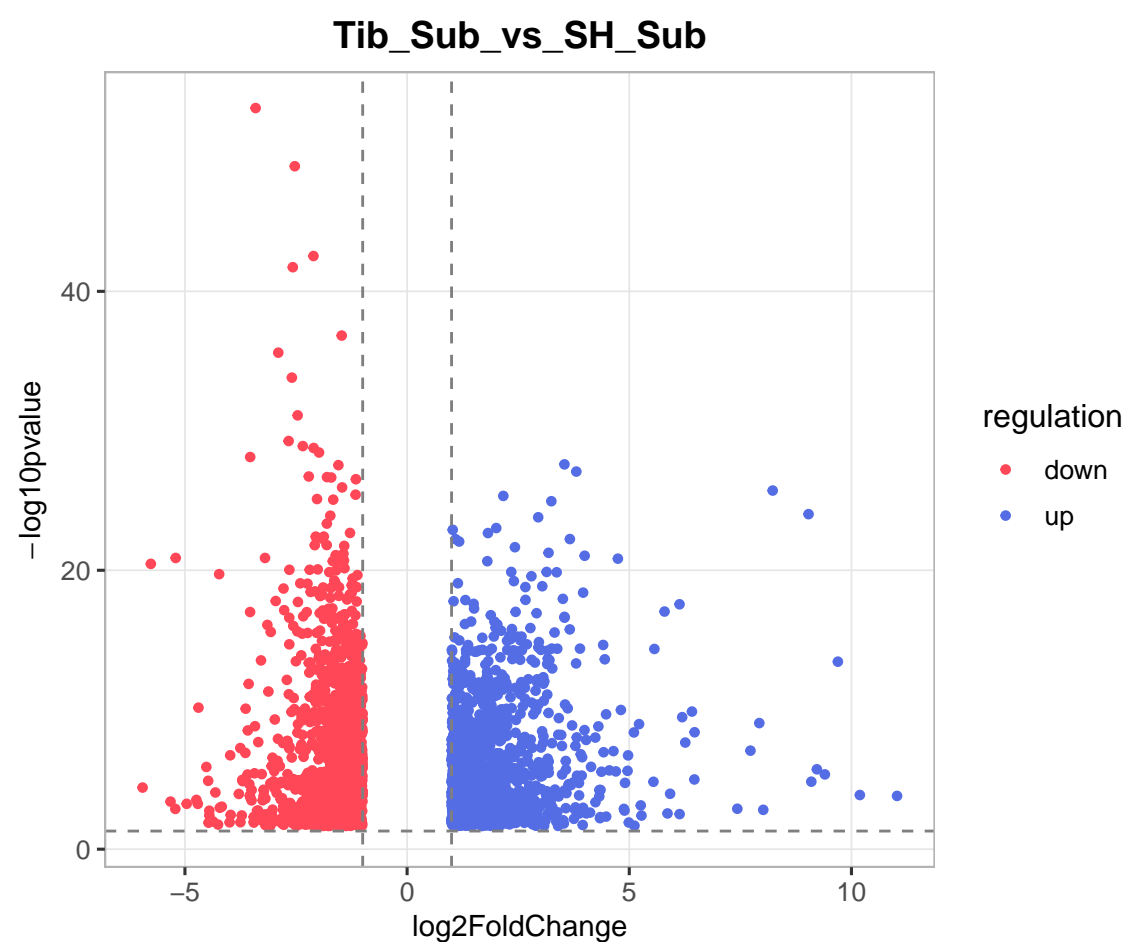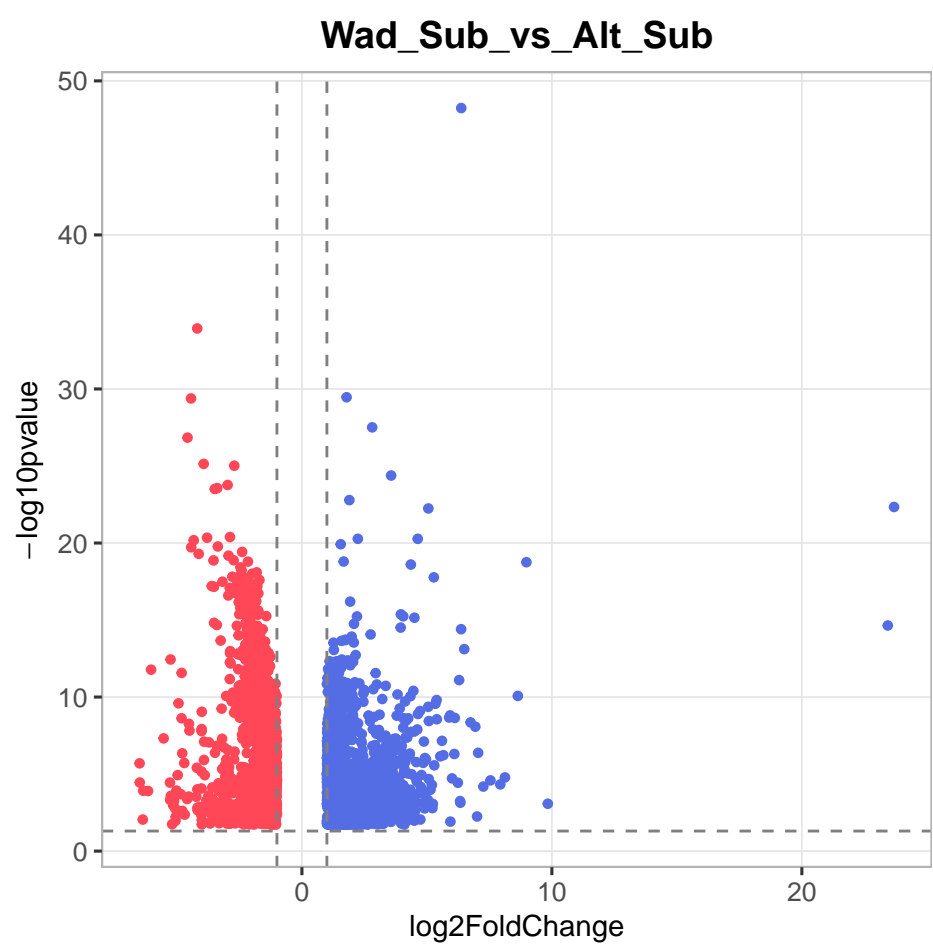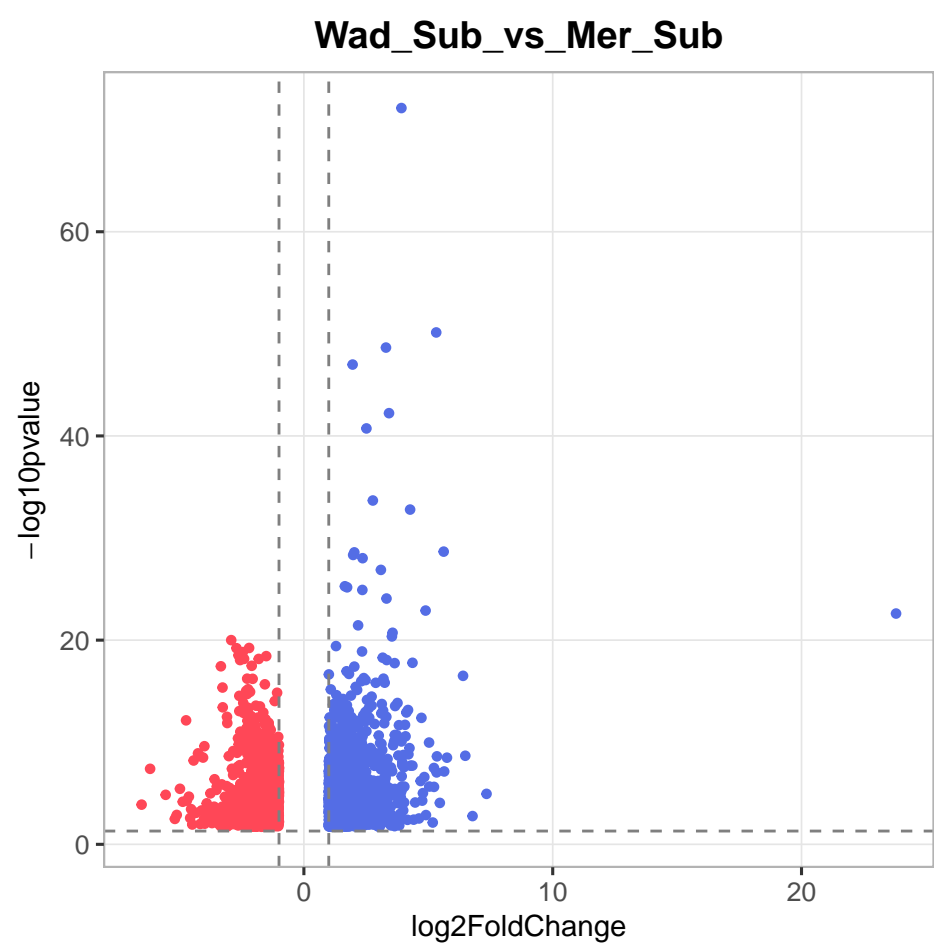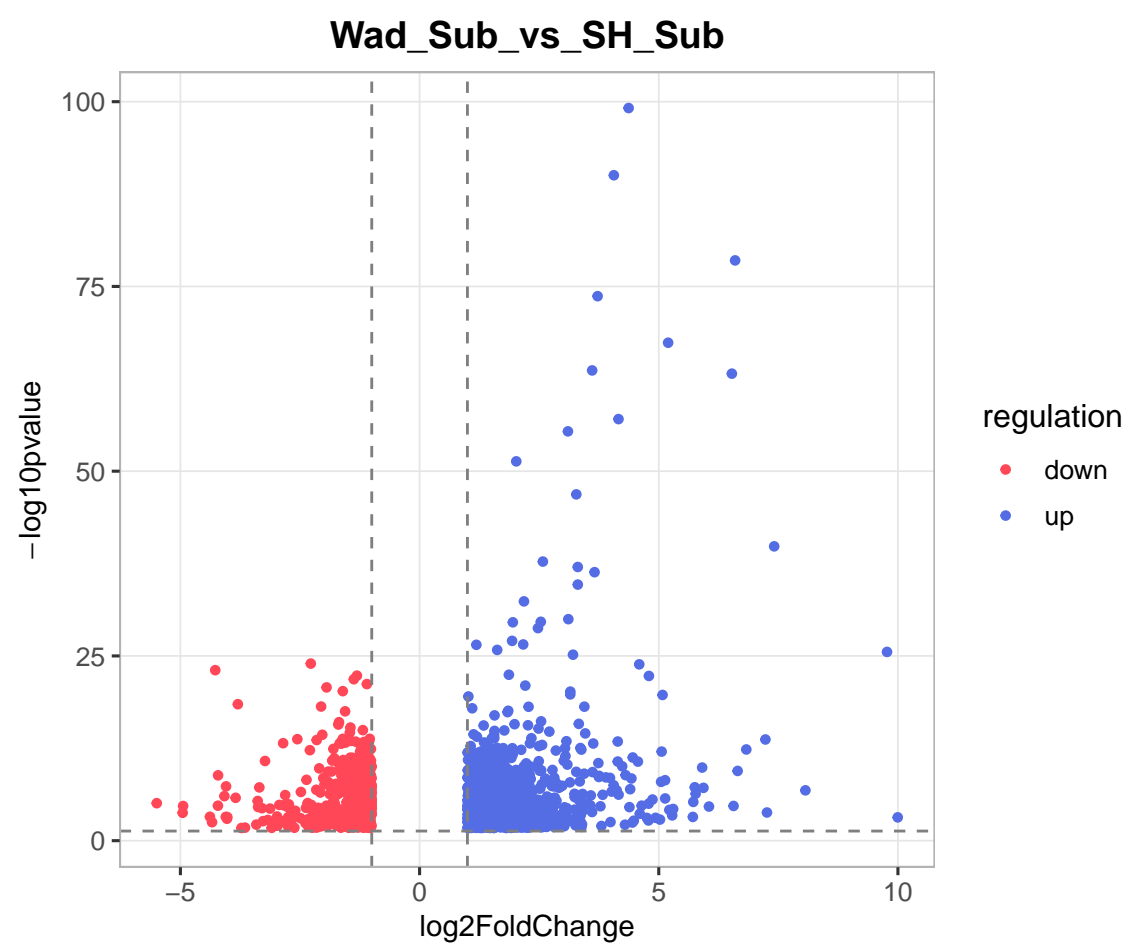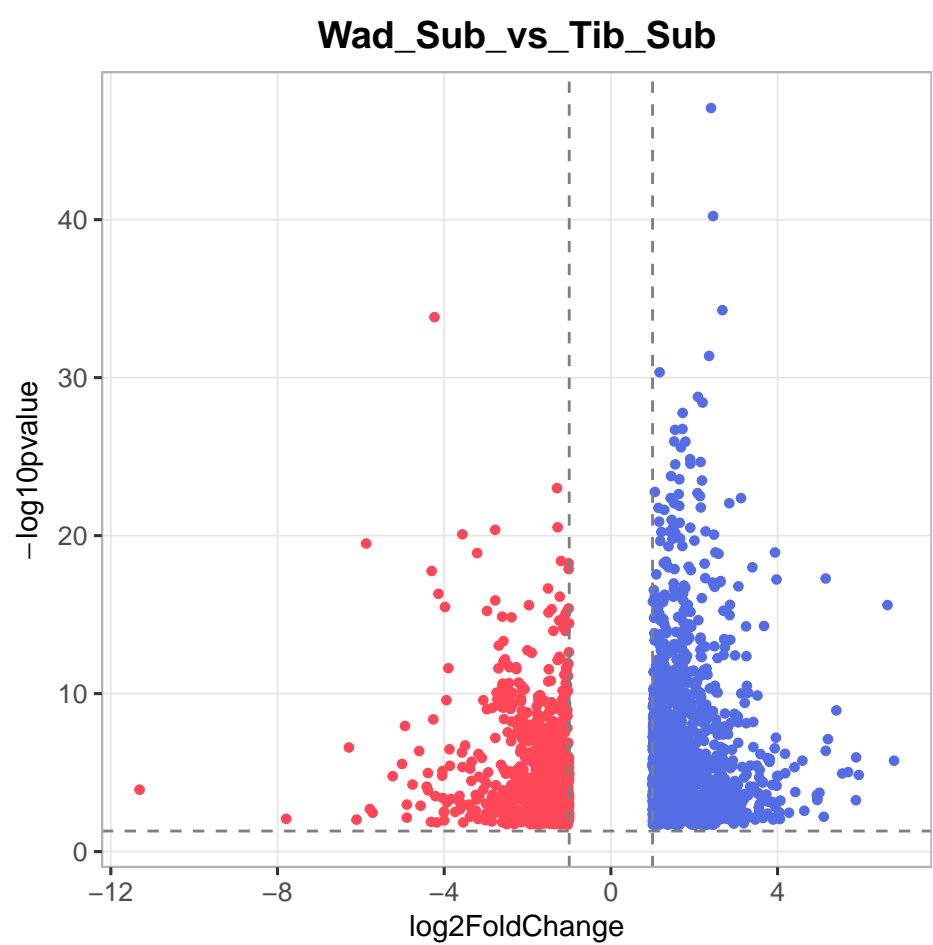

Supplement: Supplementary file 1 [file genes-17-00093-s001.zip › Supplementary Figures/Supplementary Figure 4.pdf]

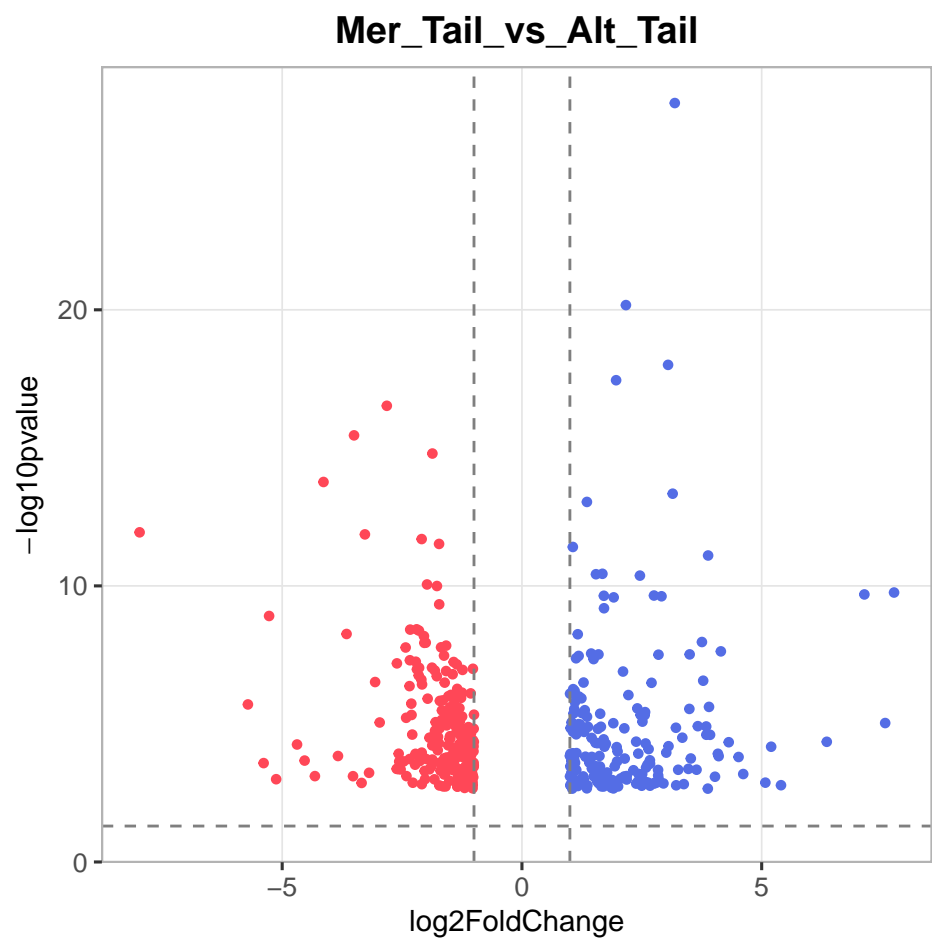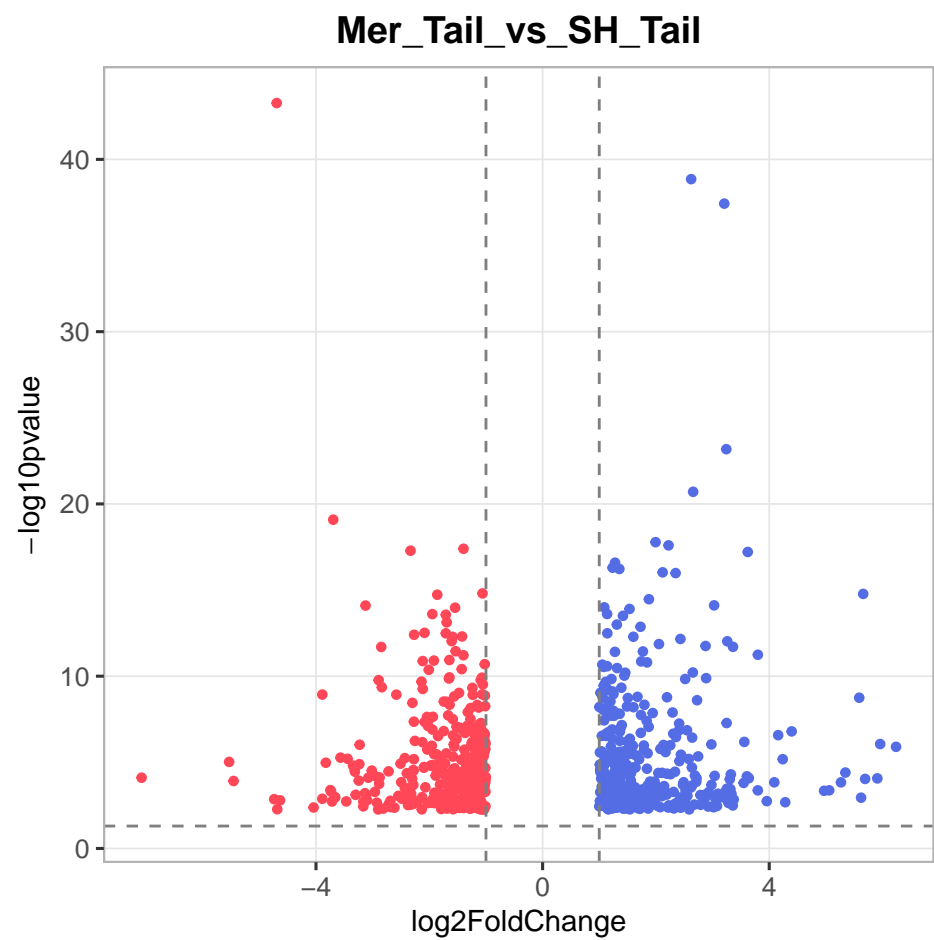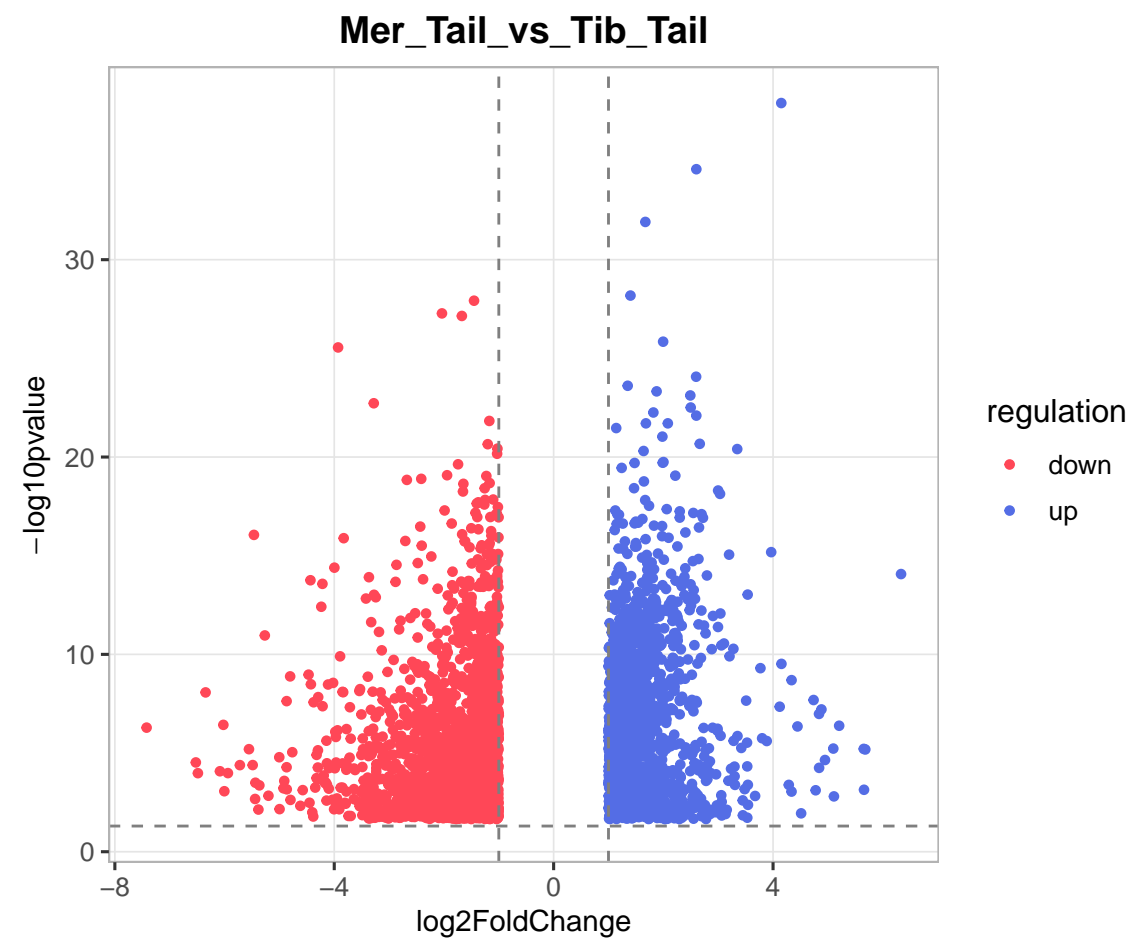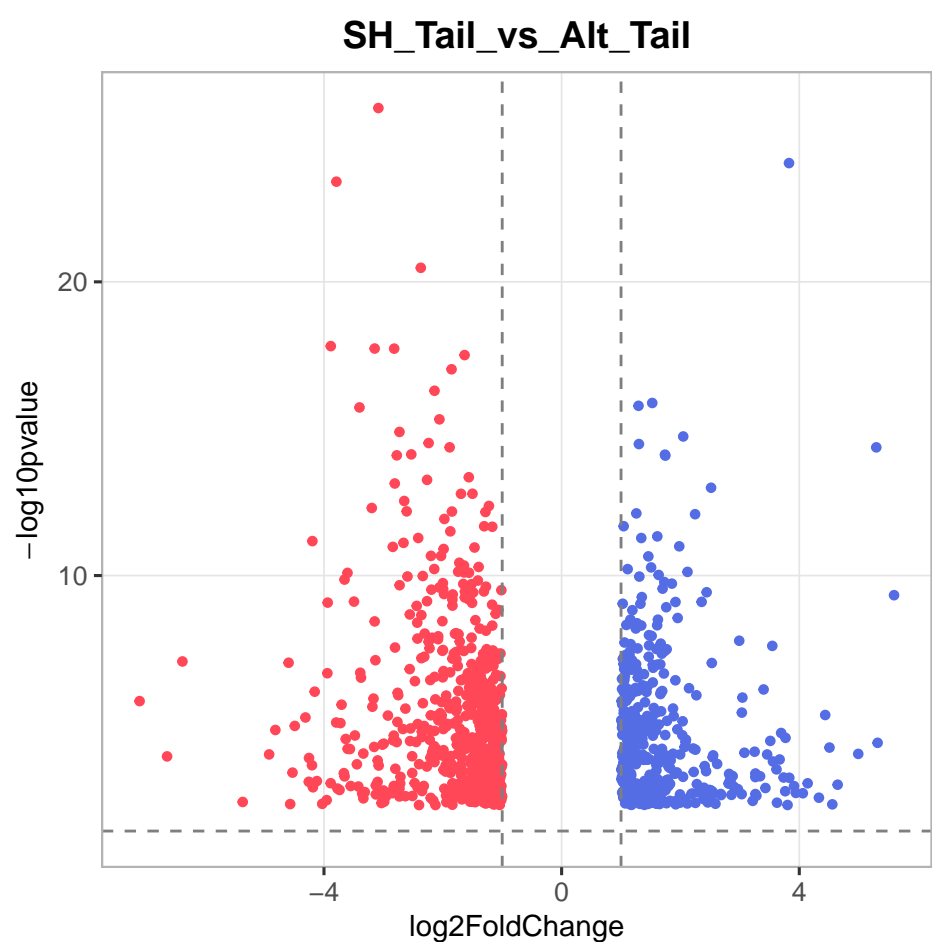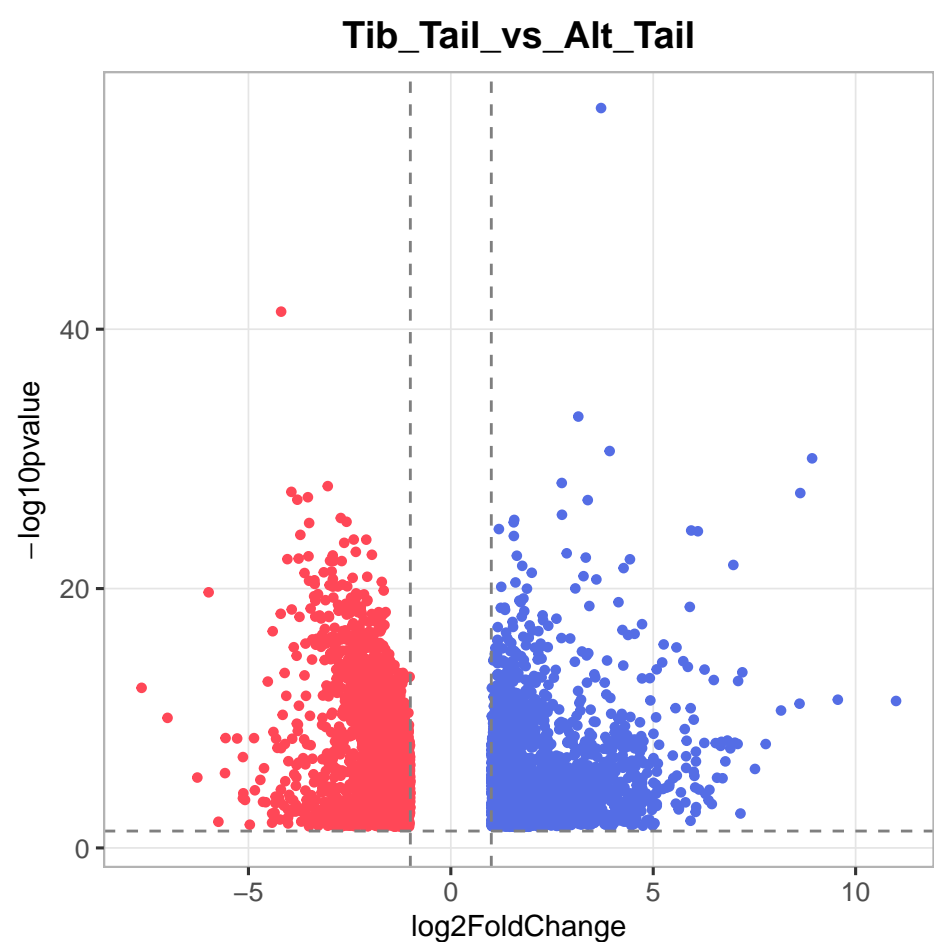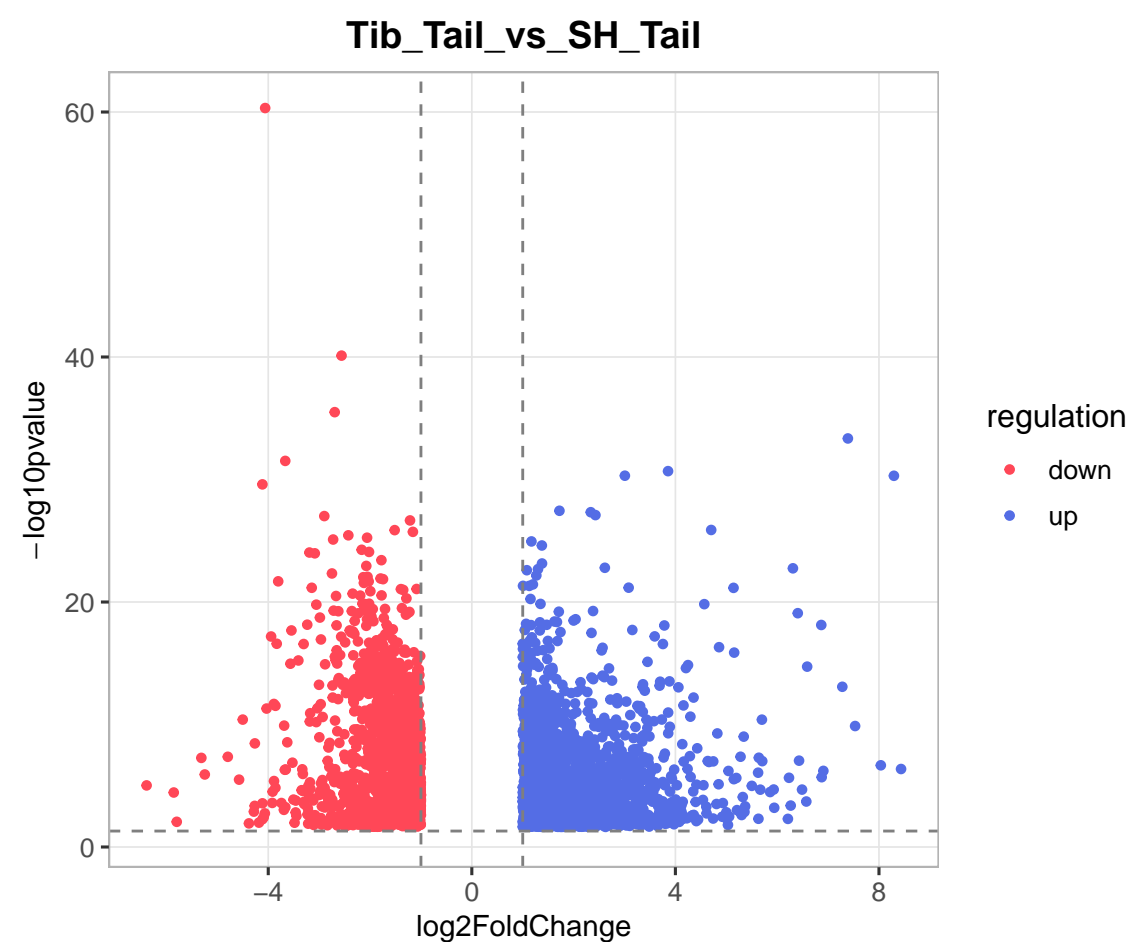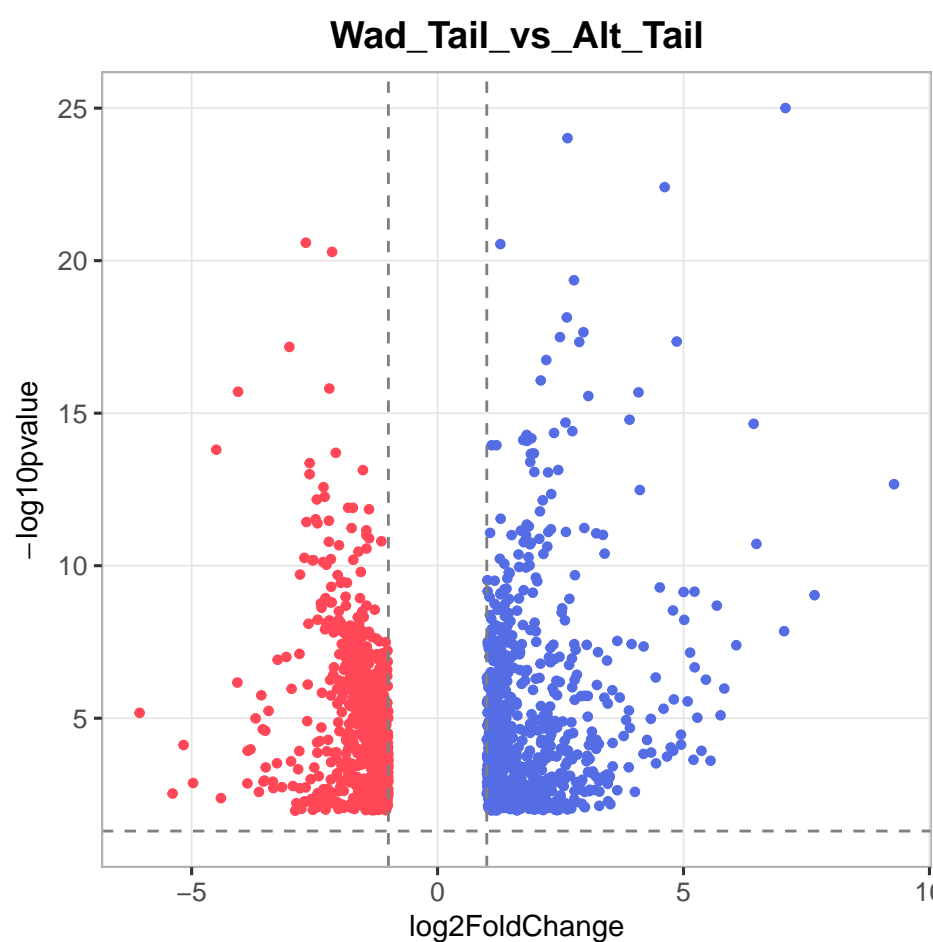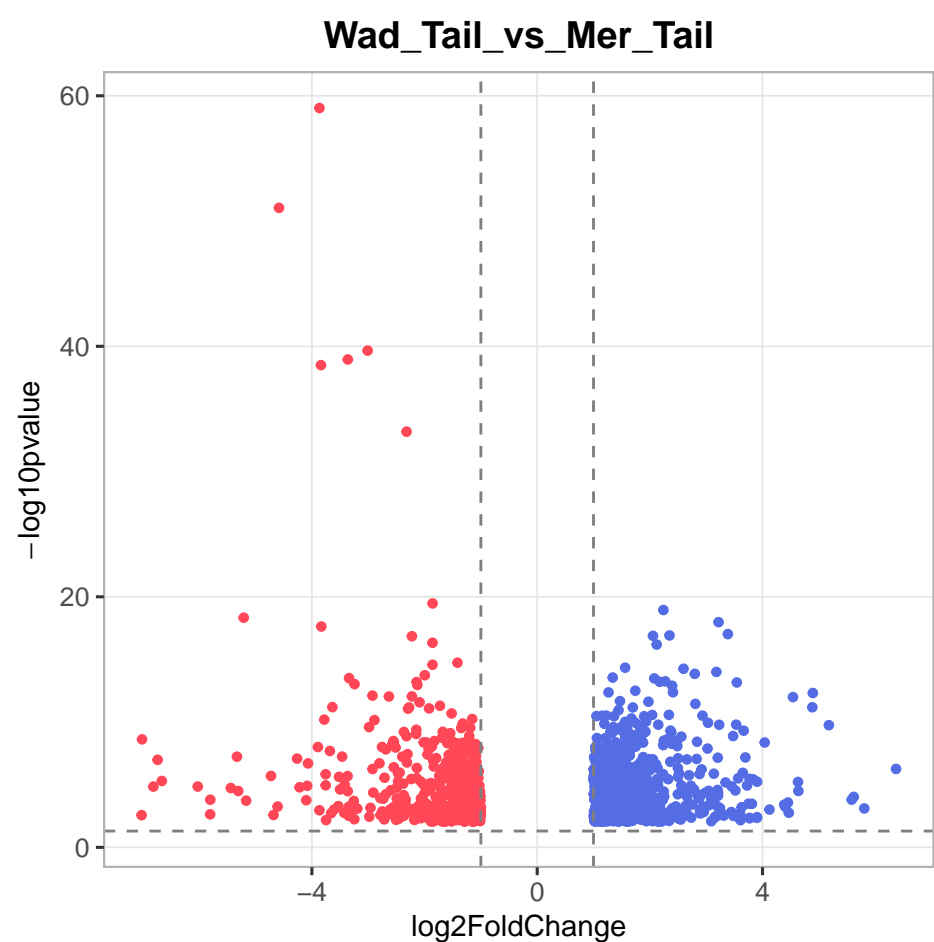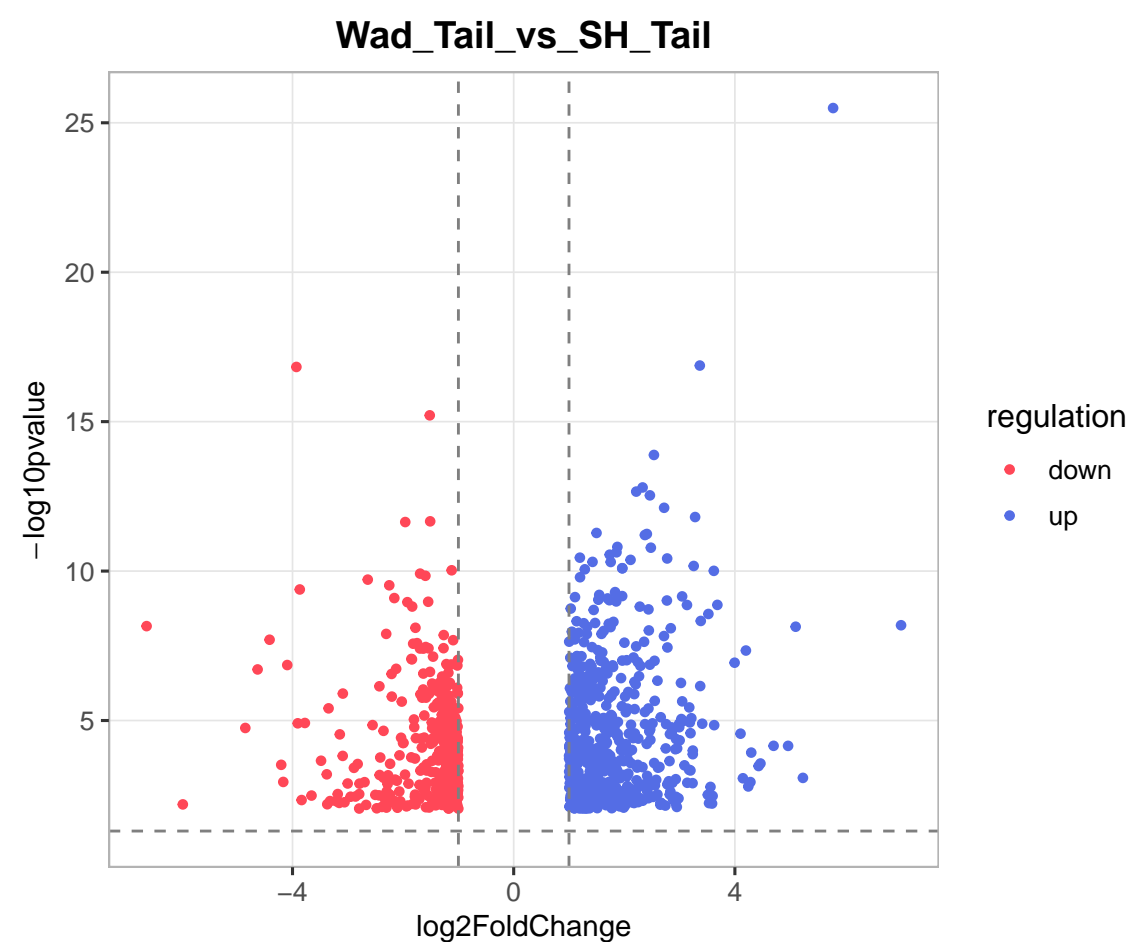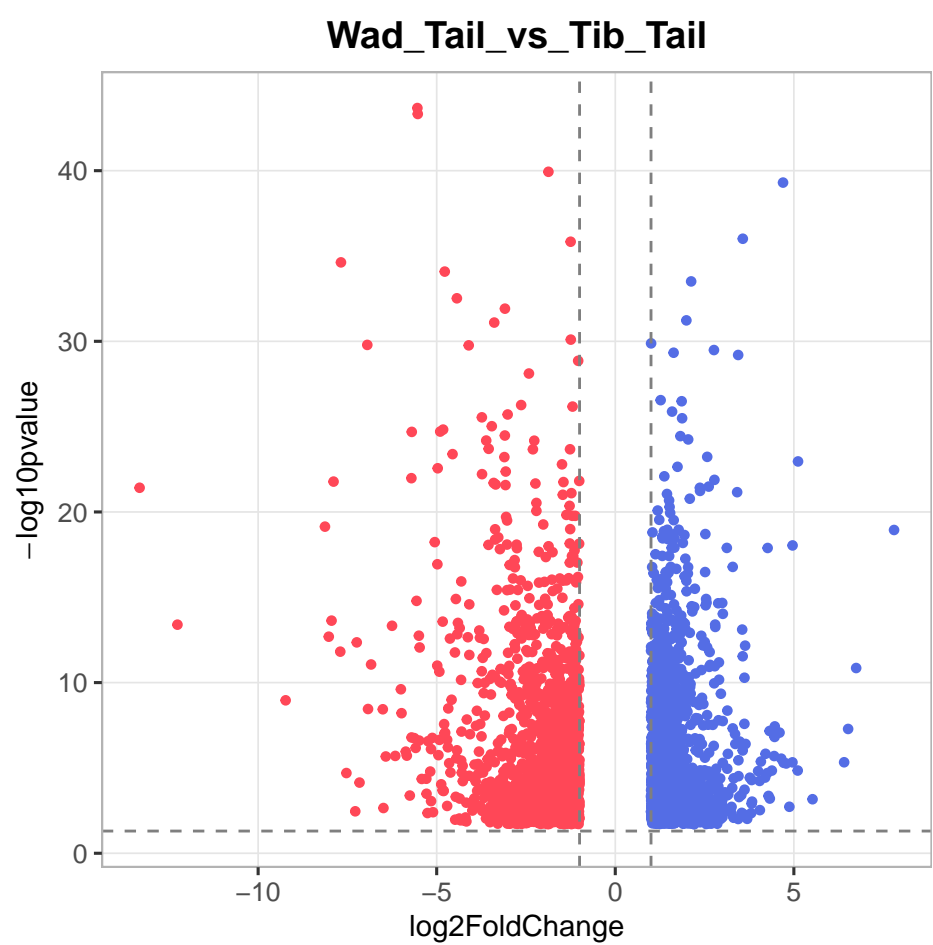

Supplement: Supplementary file 1 [file genes-17-00093-s001.zip › Supplementary Figures/Supplementary Figure 5.pdf]

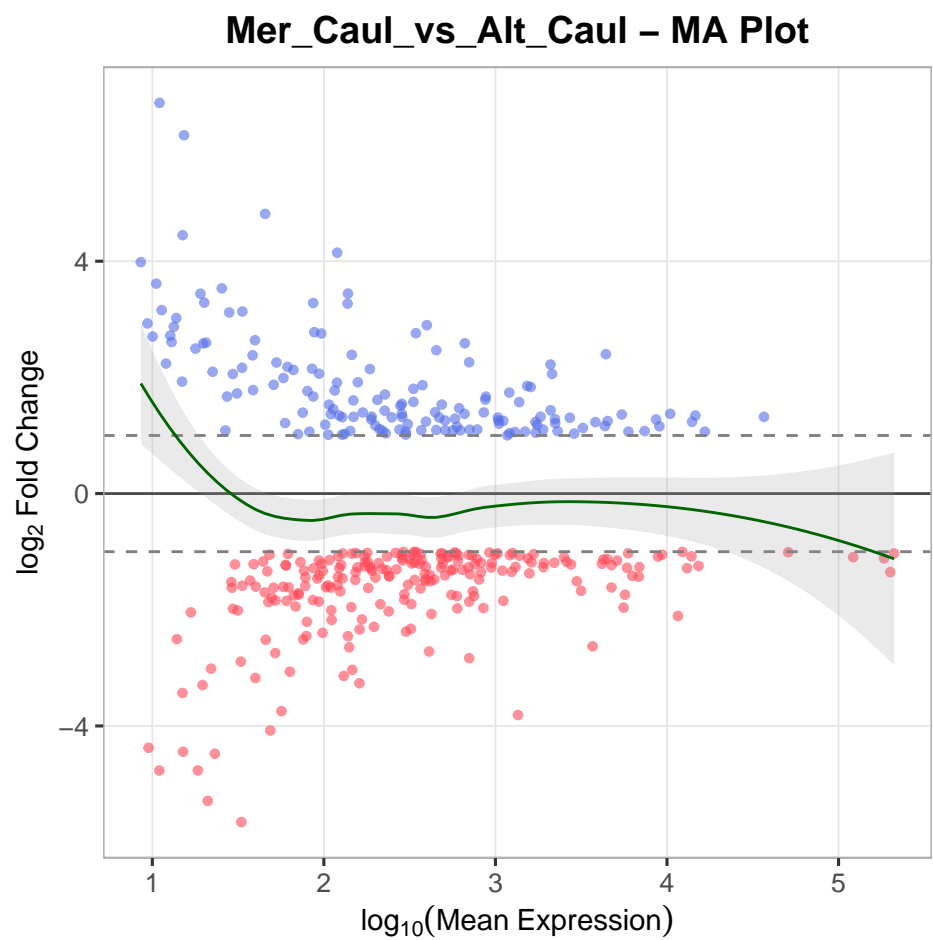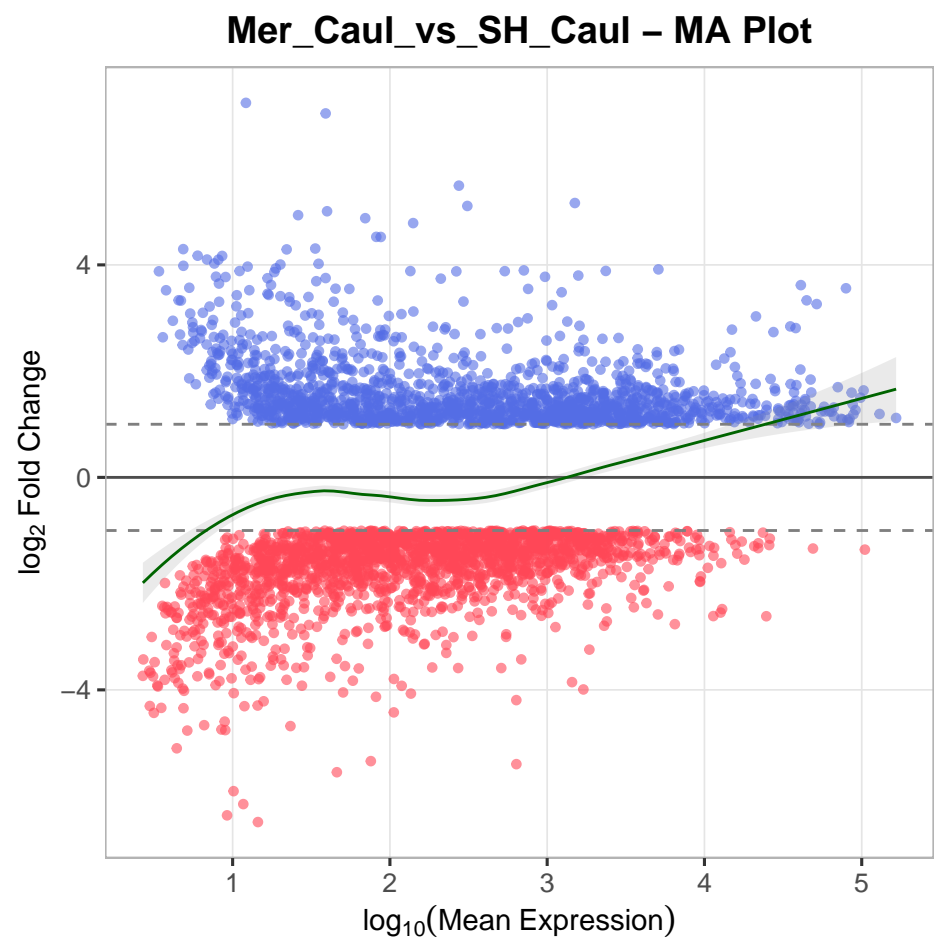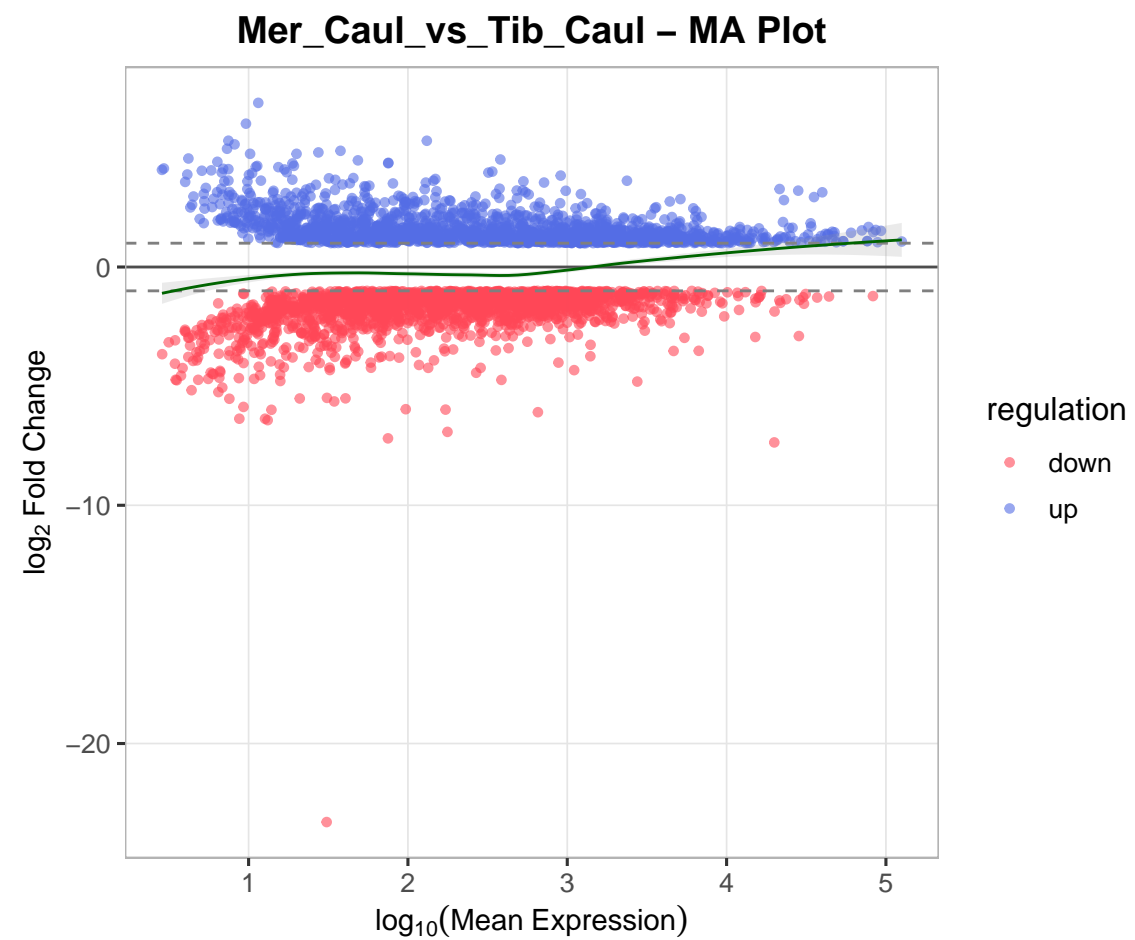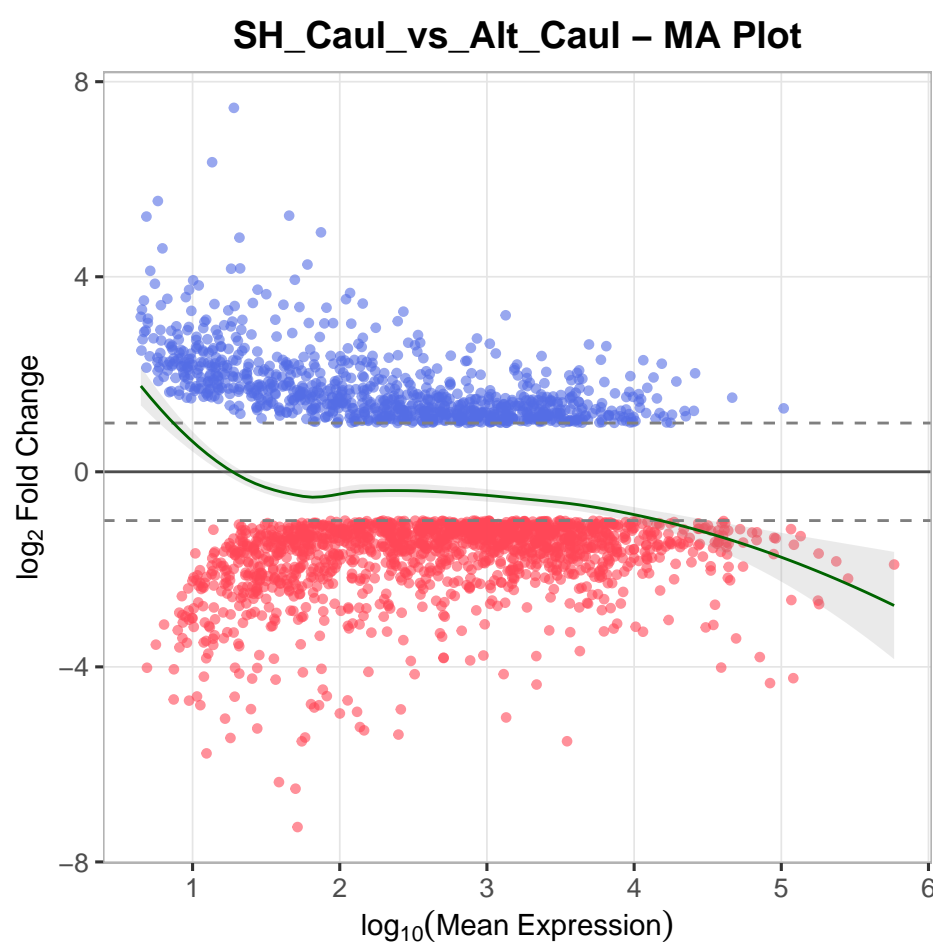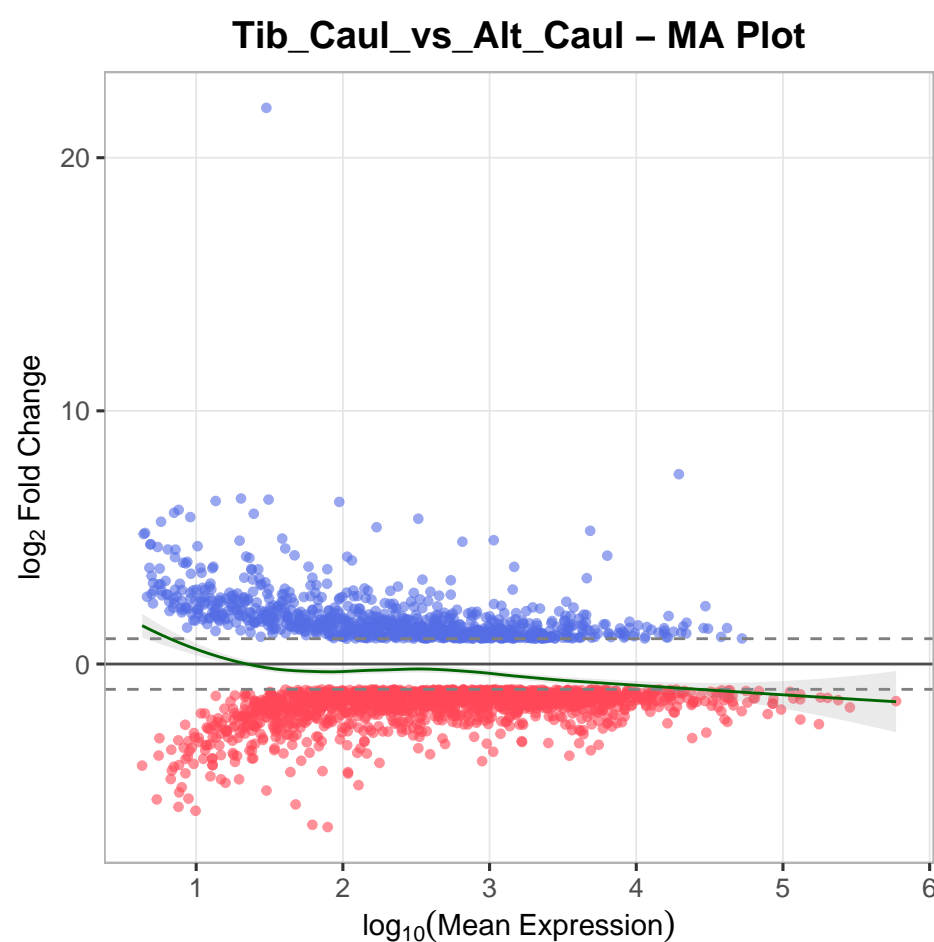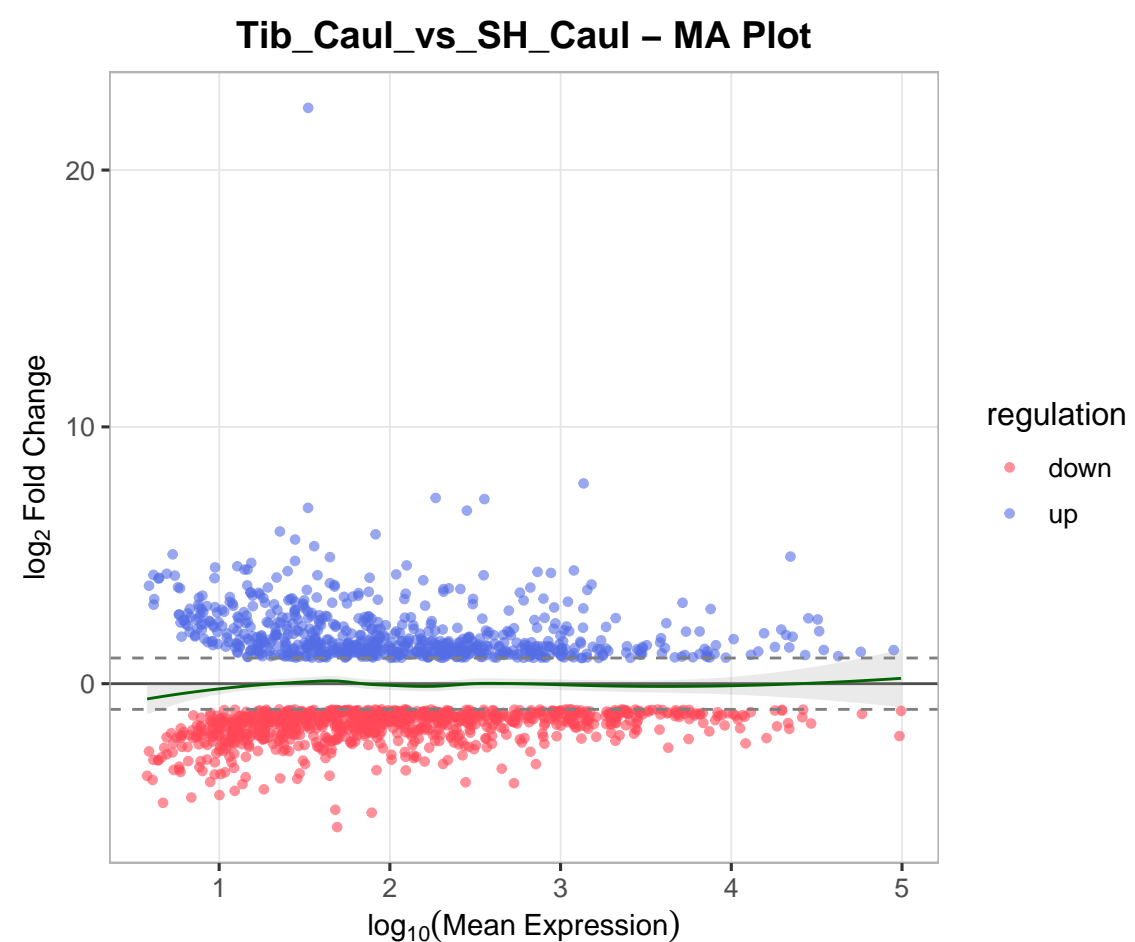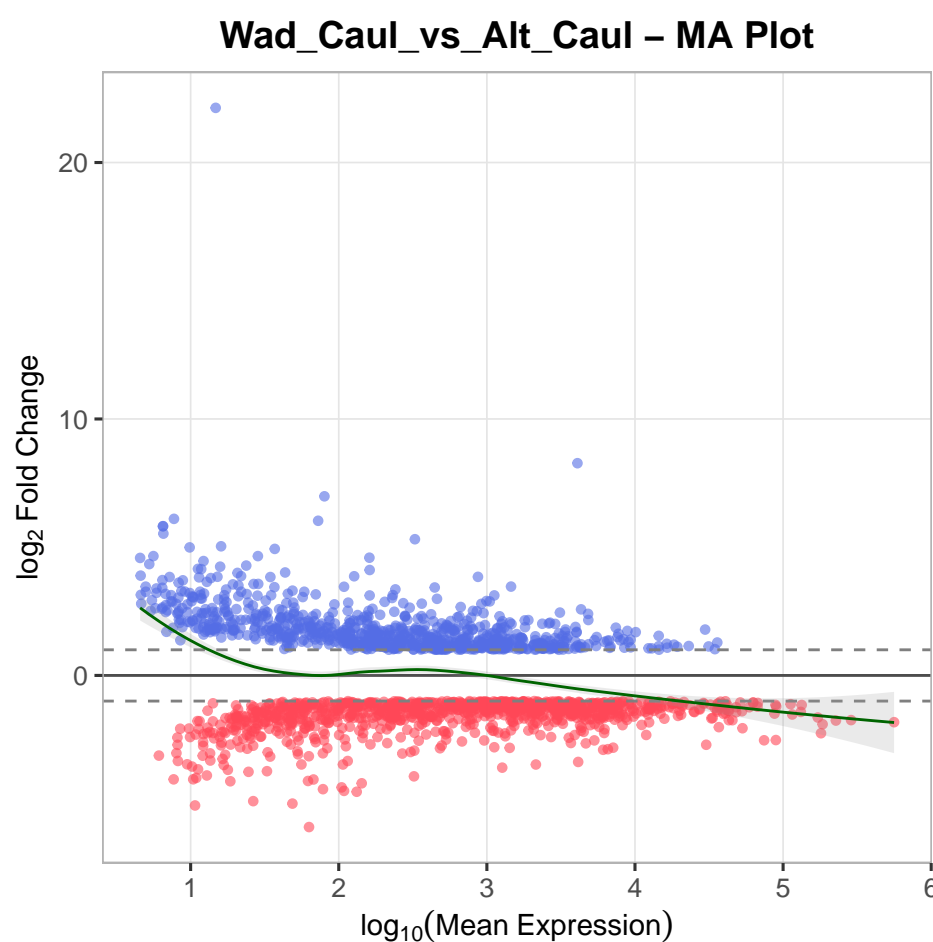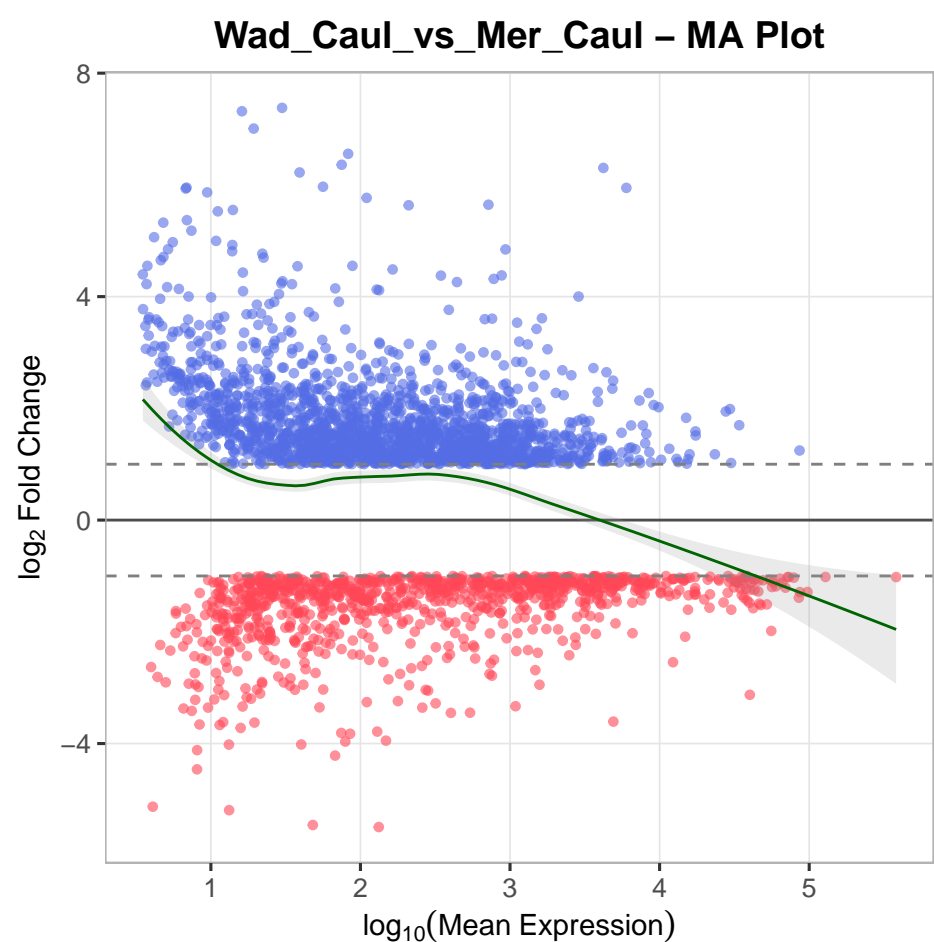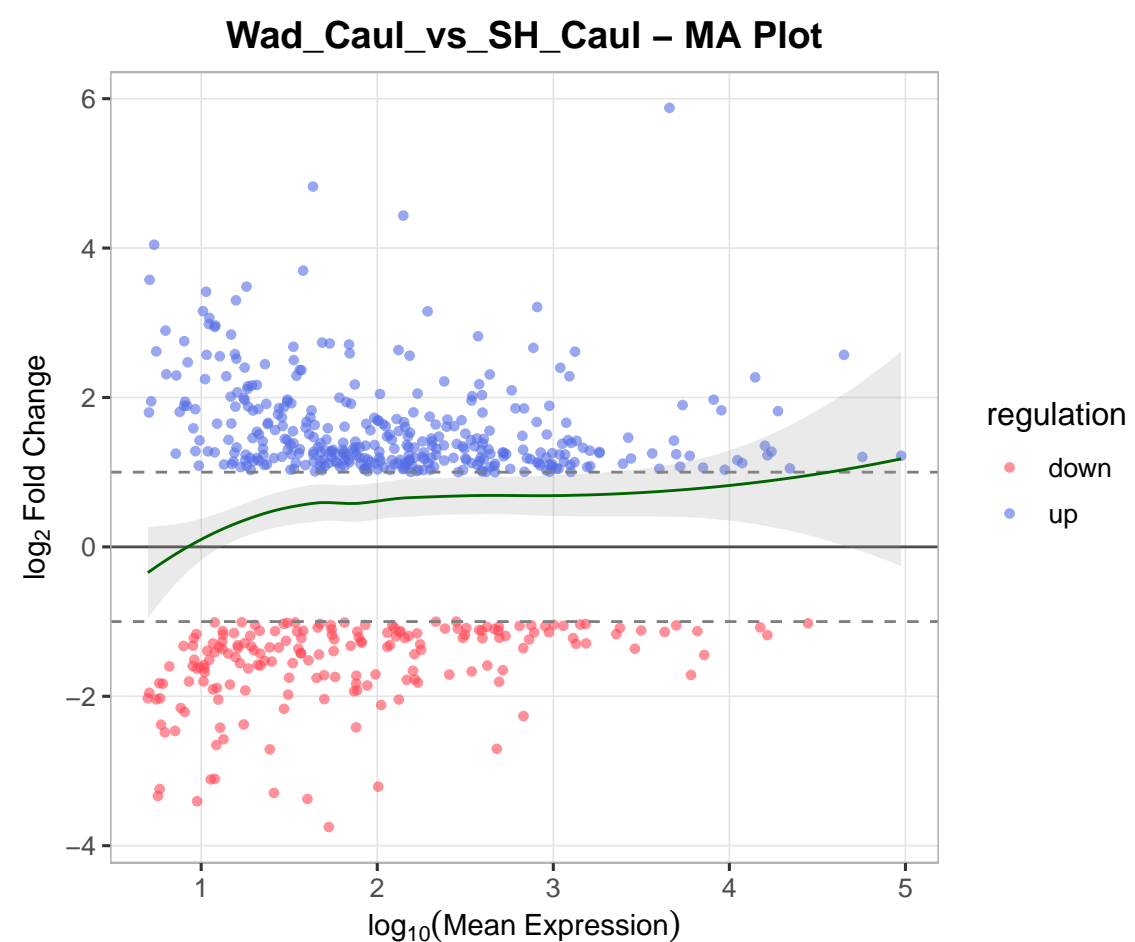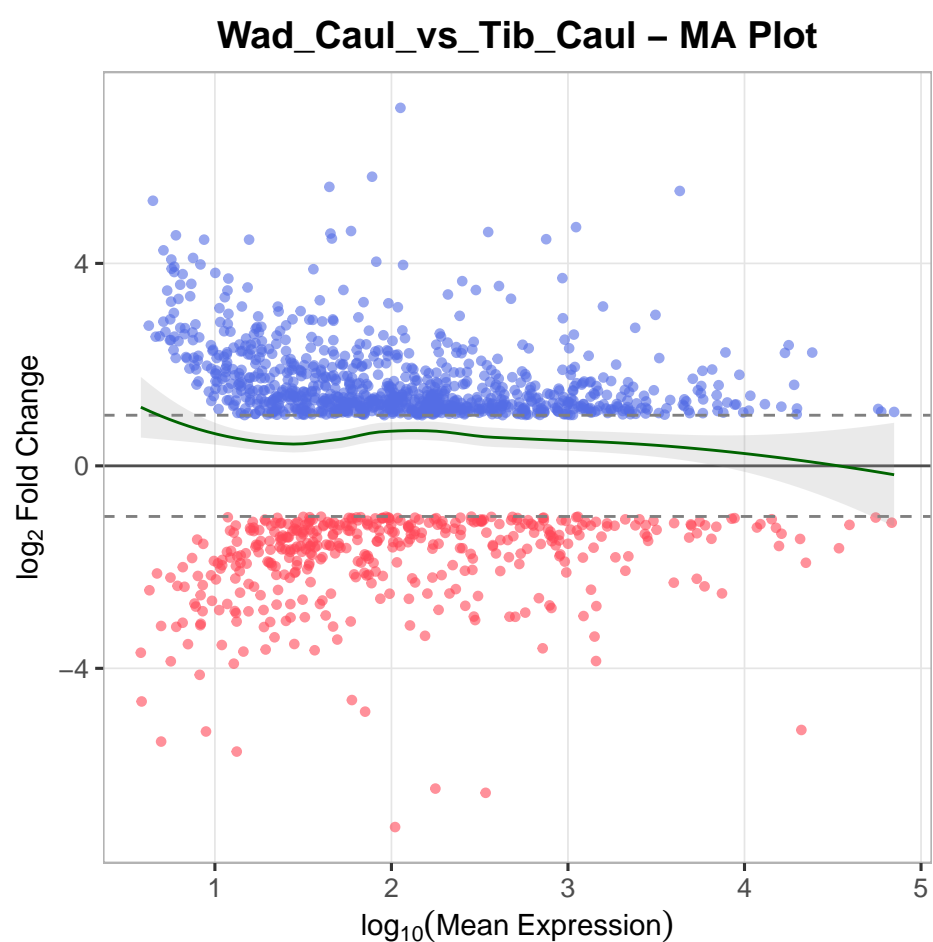

Supplement: Supplementary file 1 [file genes-17-00093-s001.zip › Supplementary Figures/Supplementary Figure 6.pdf]

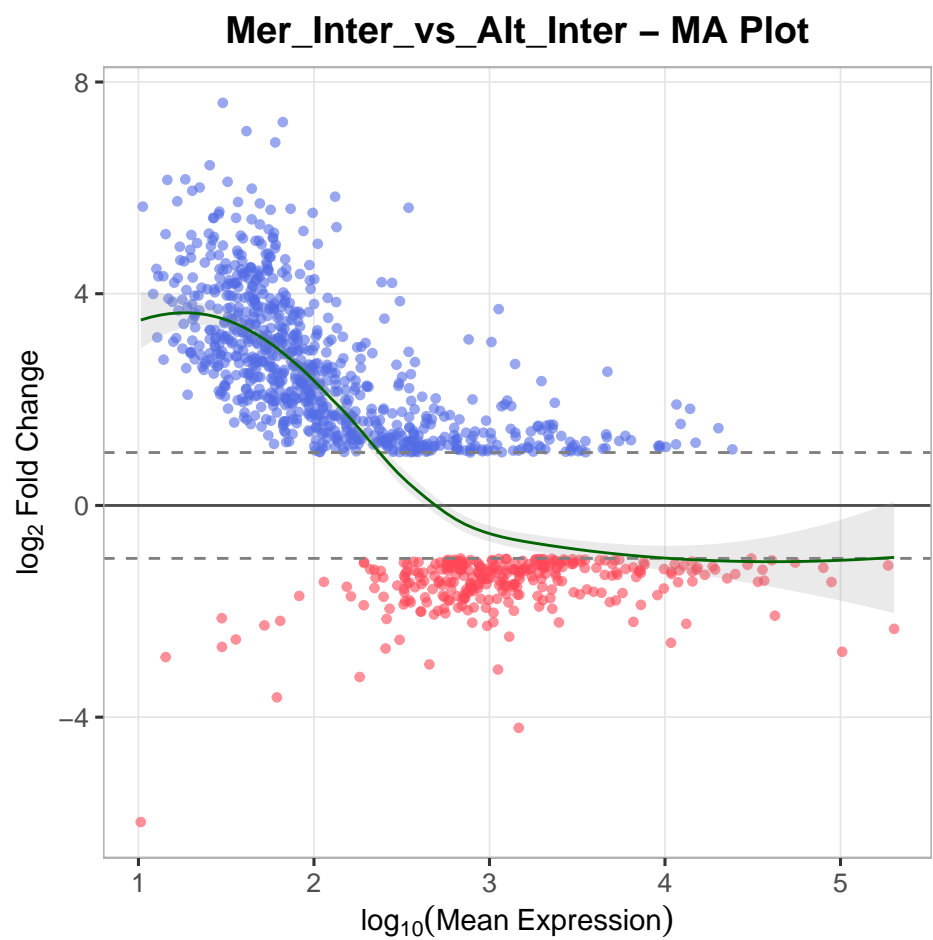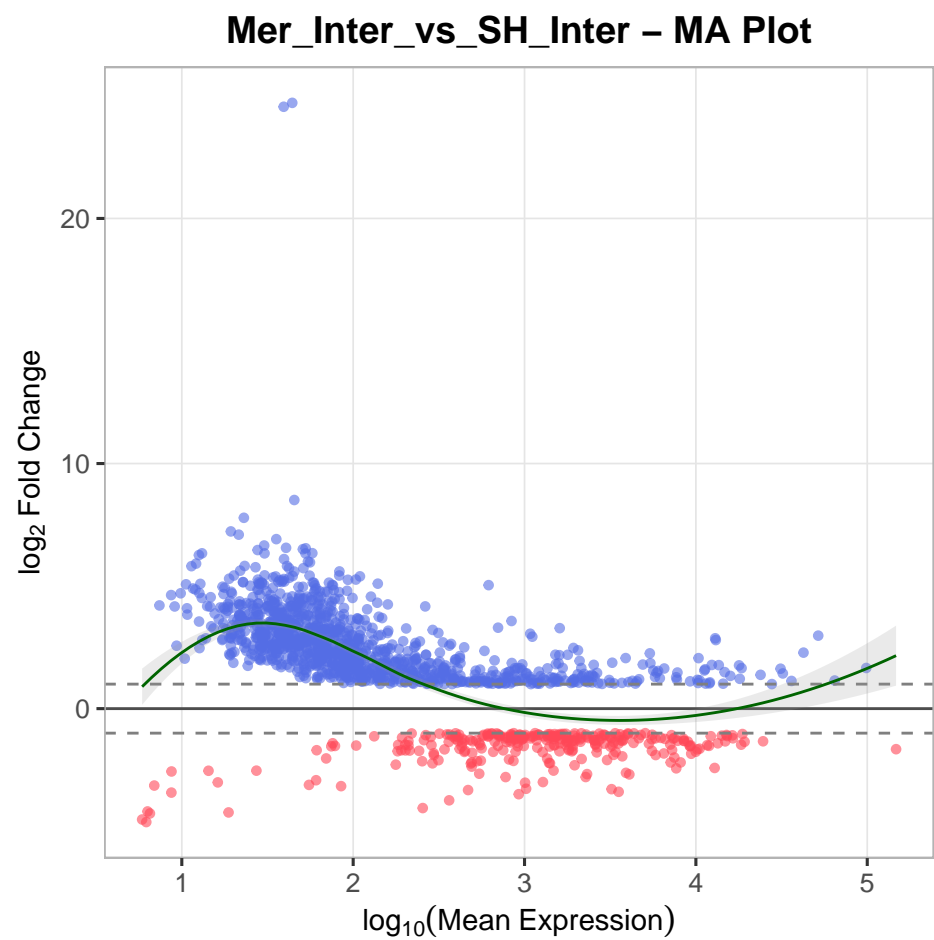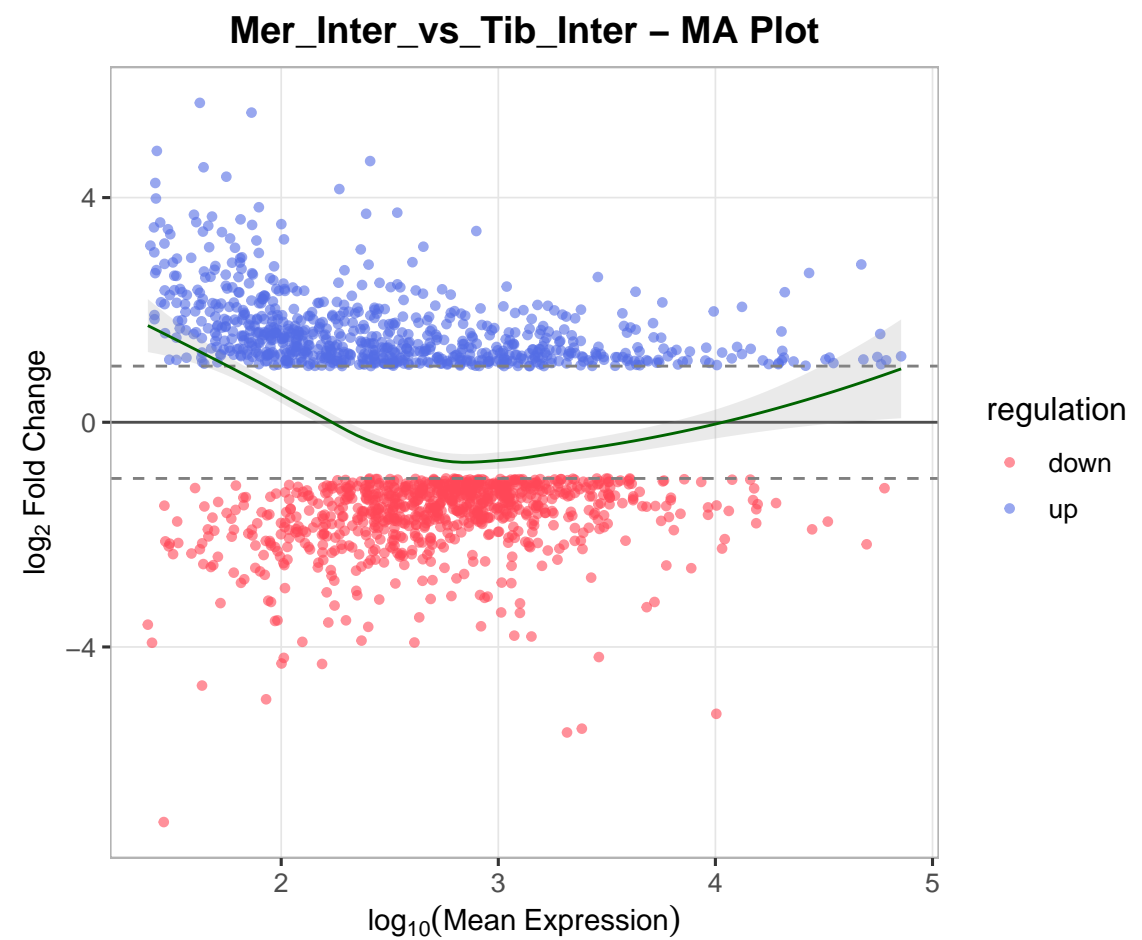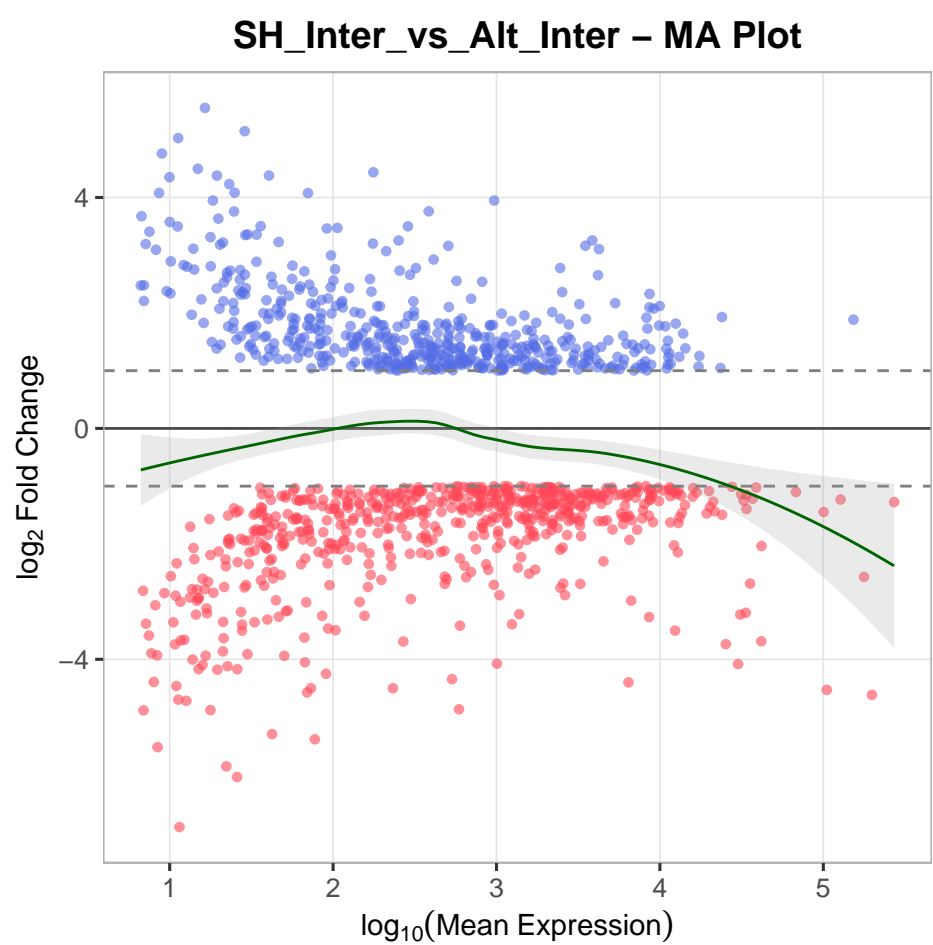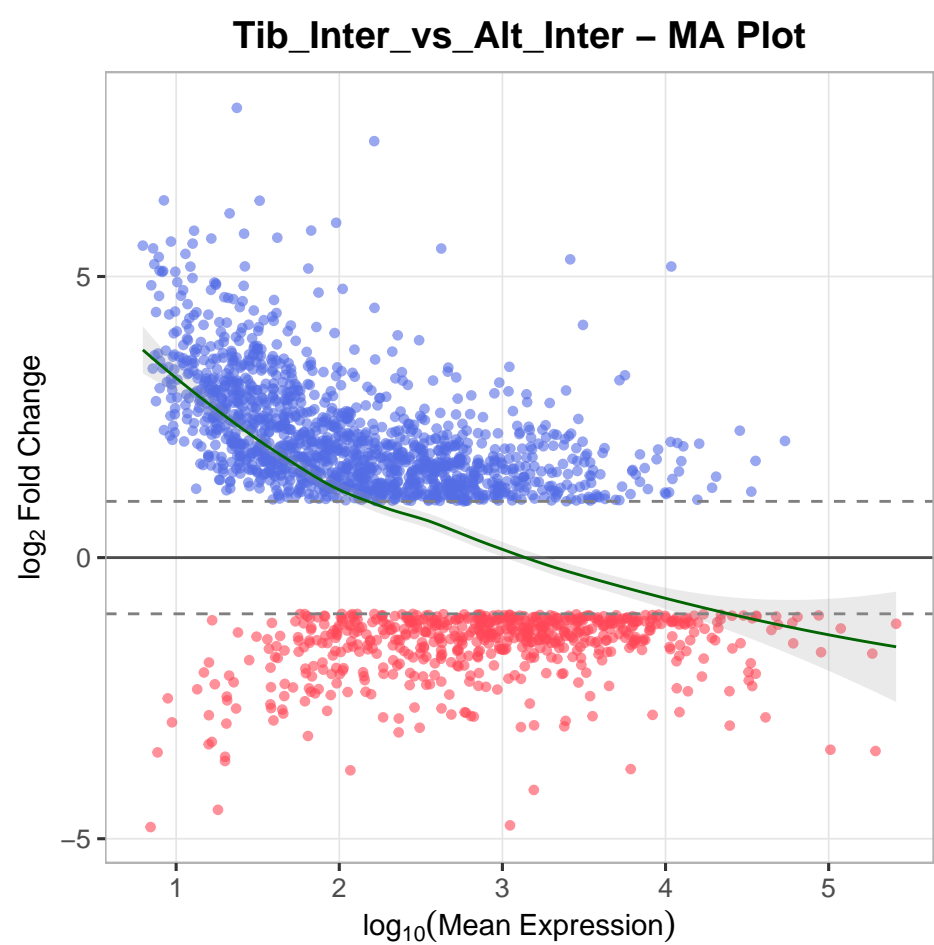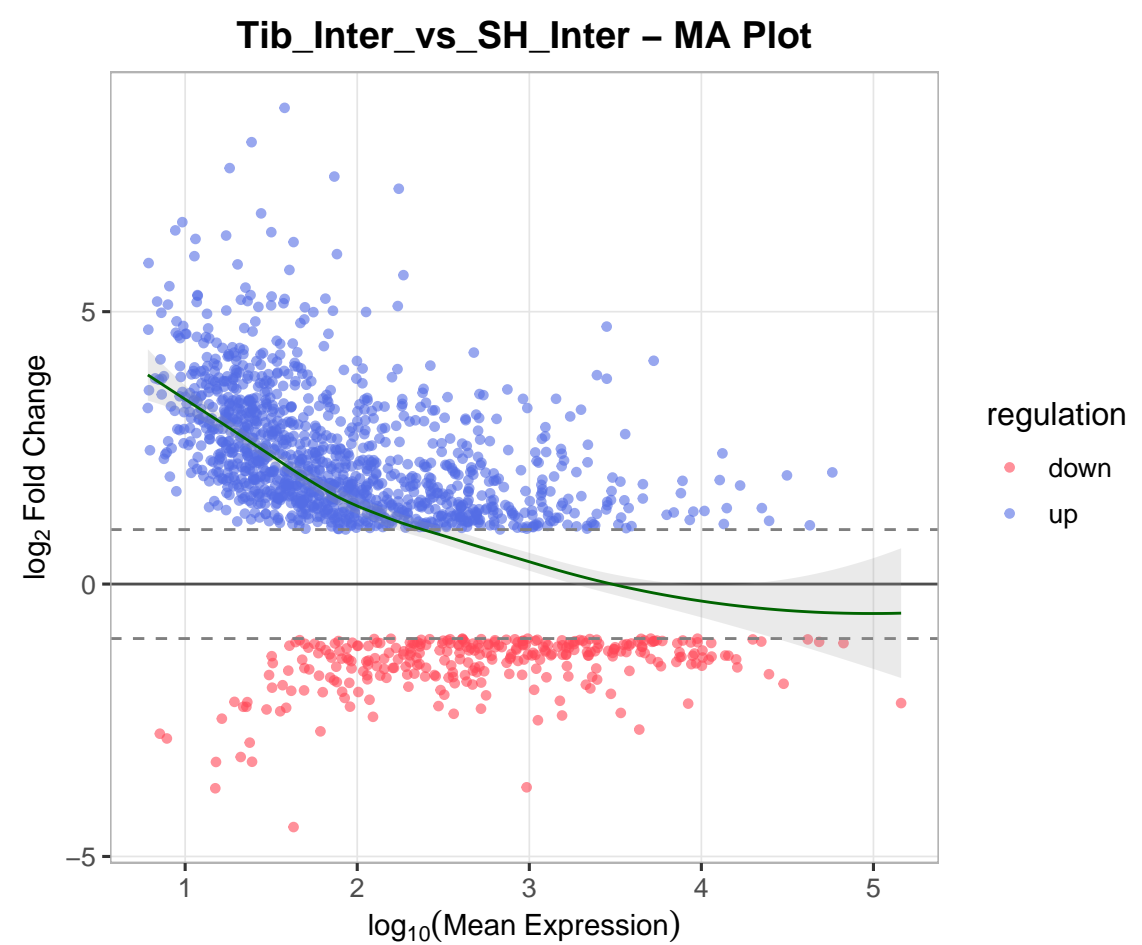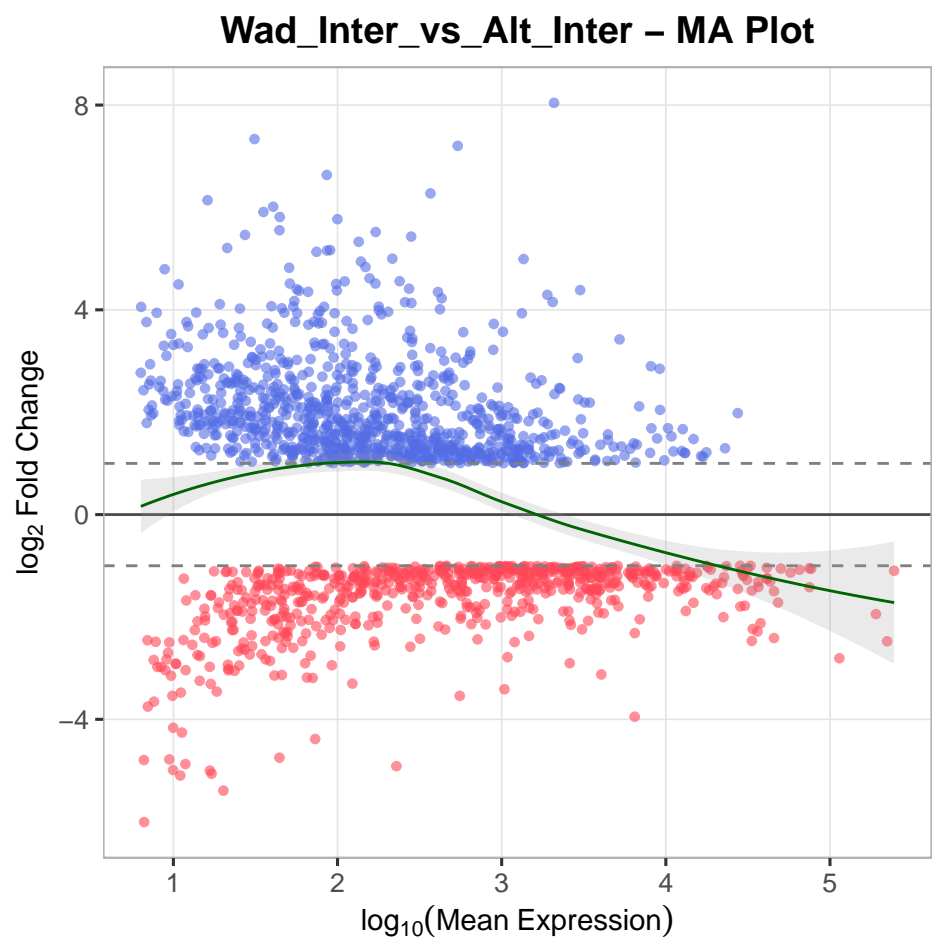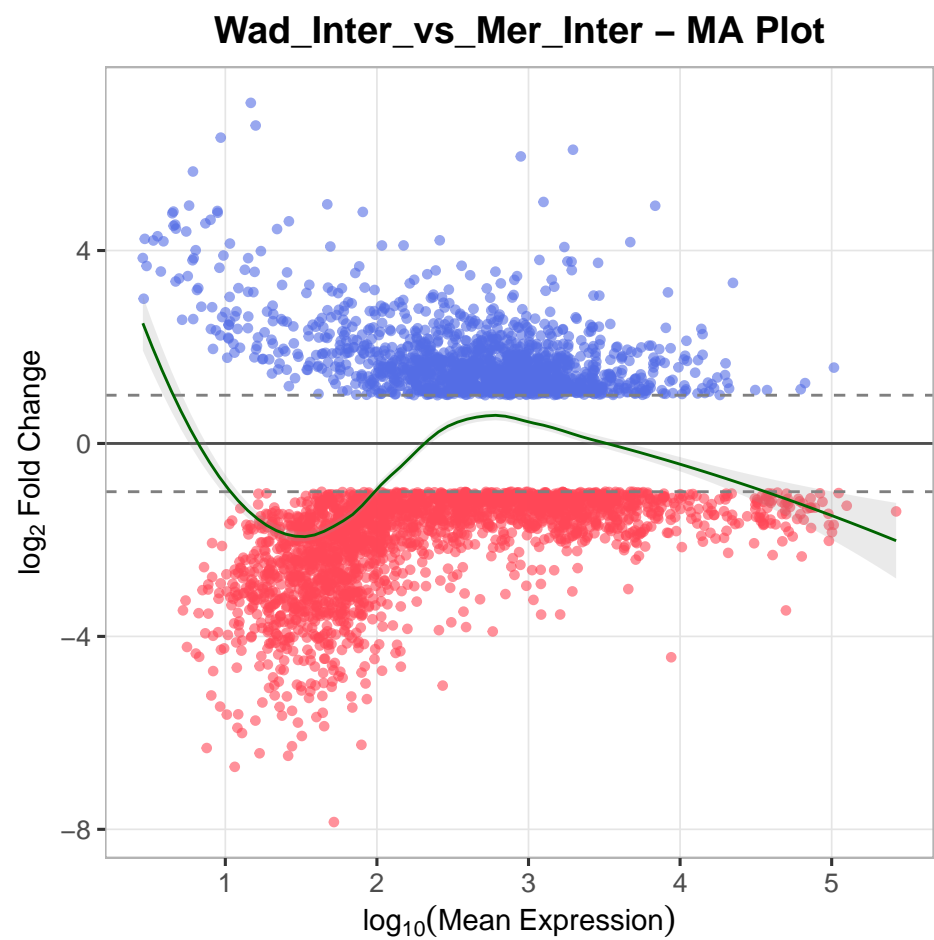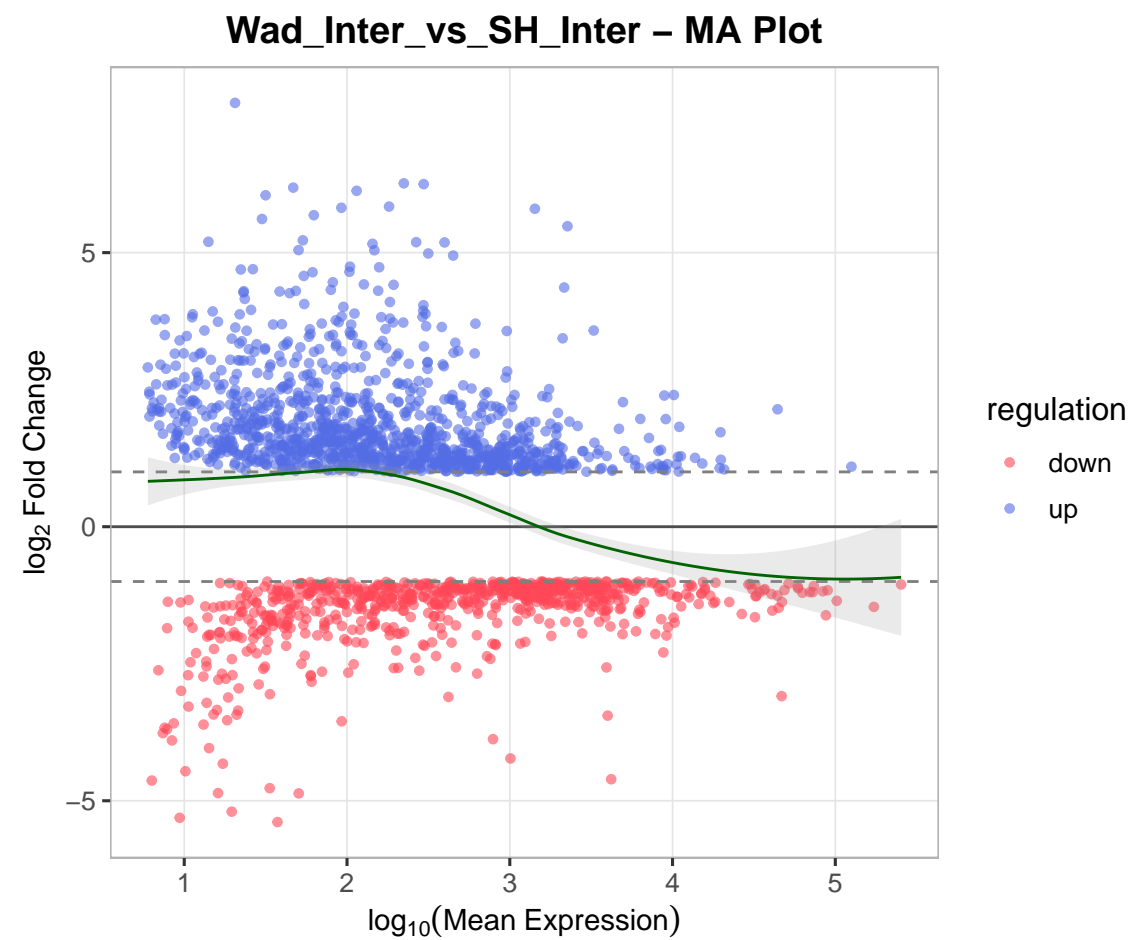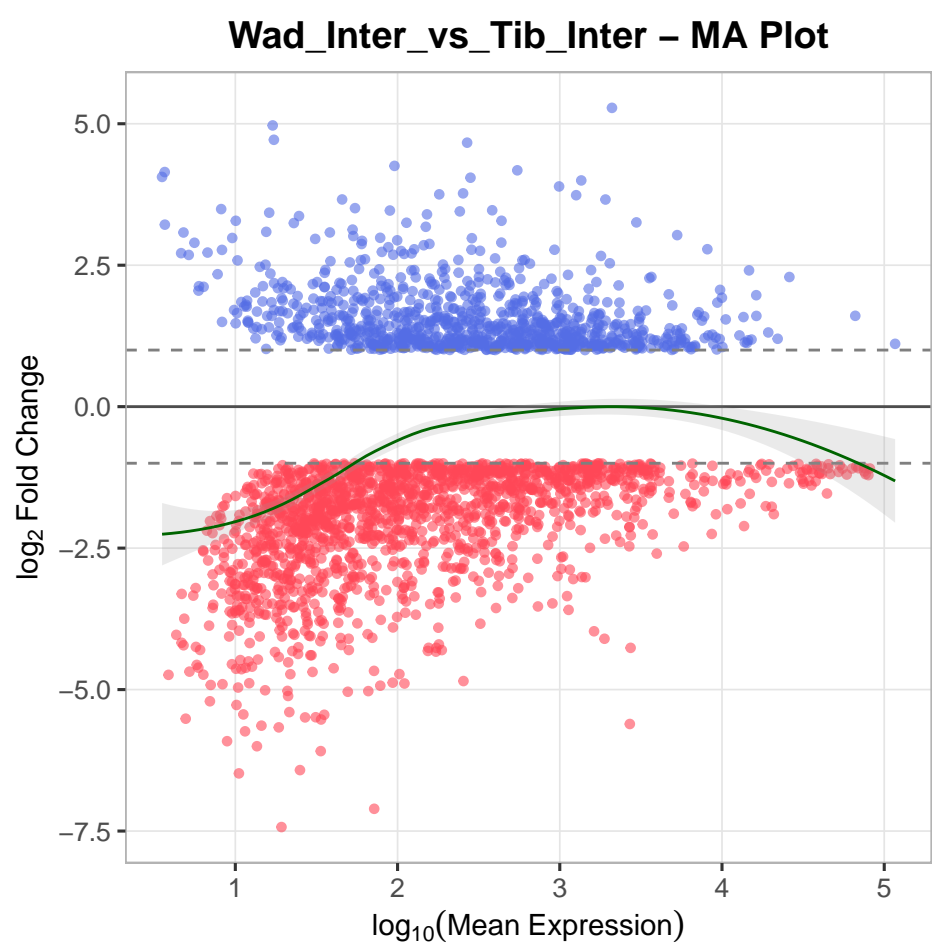

Supplement: Supplementary file 1 [file genes-17-00093-s001.zip › Supplementary Figures/Supplementary Figure 7.pdf]

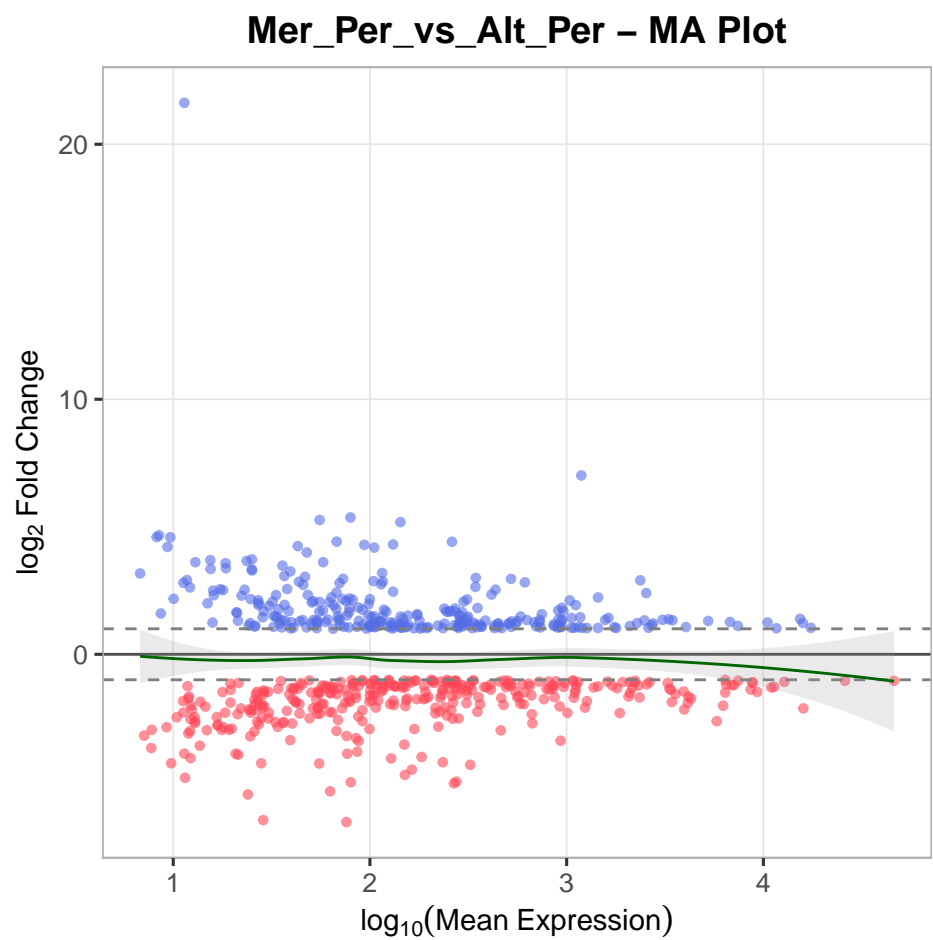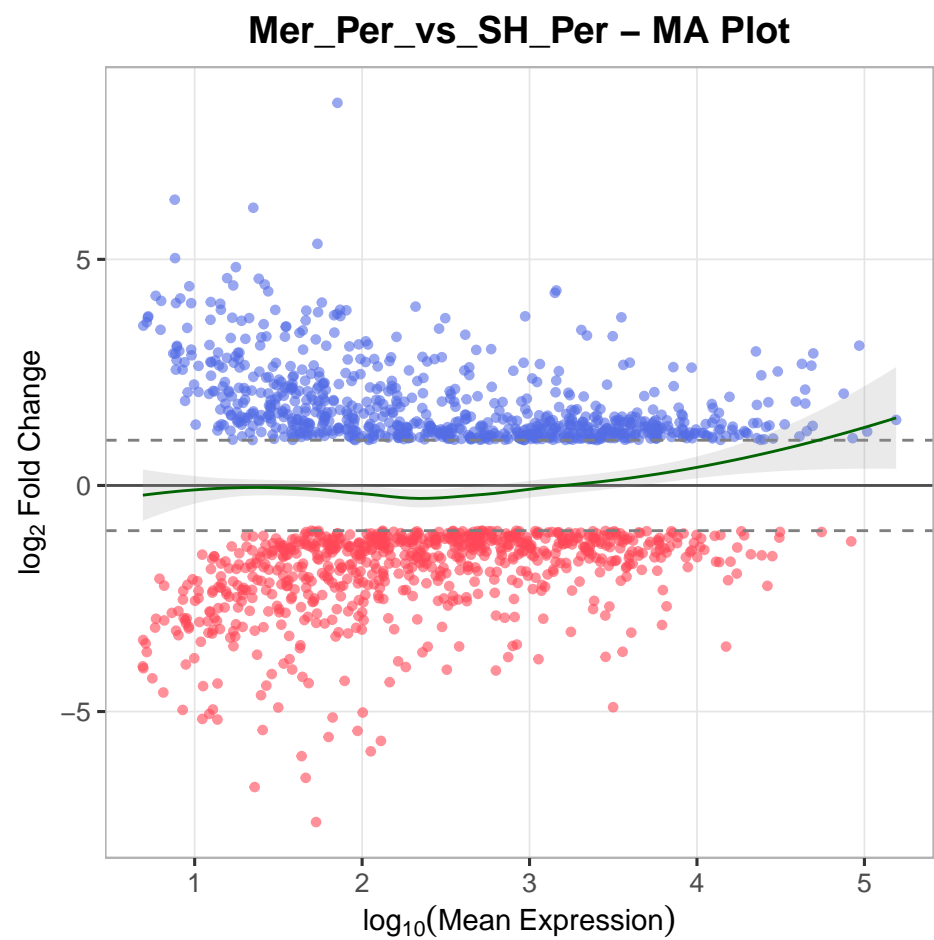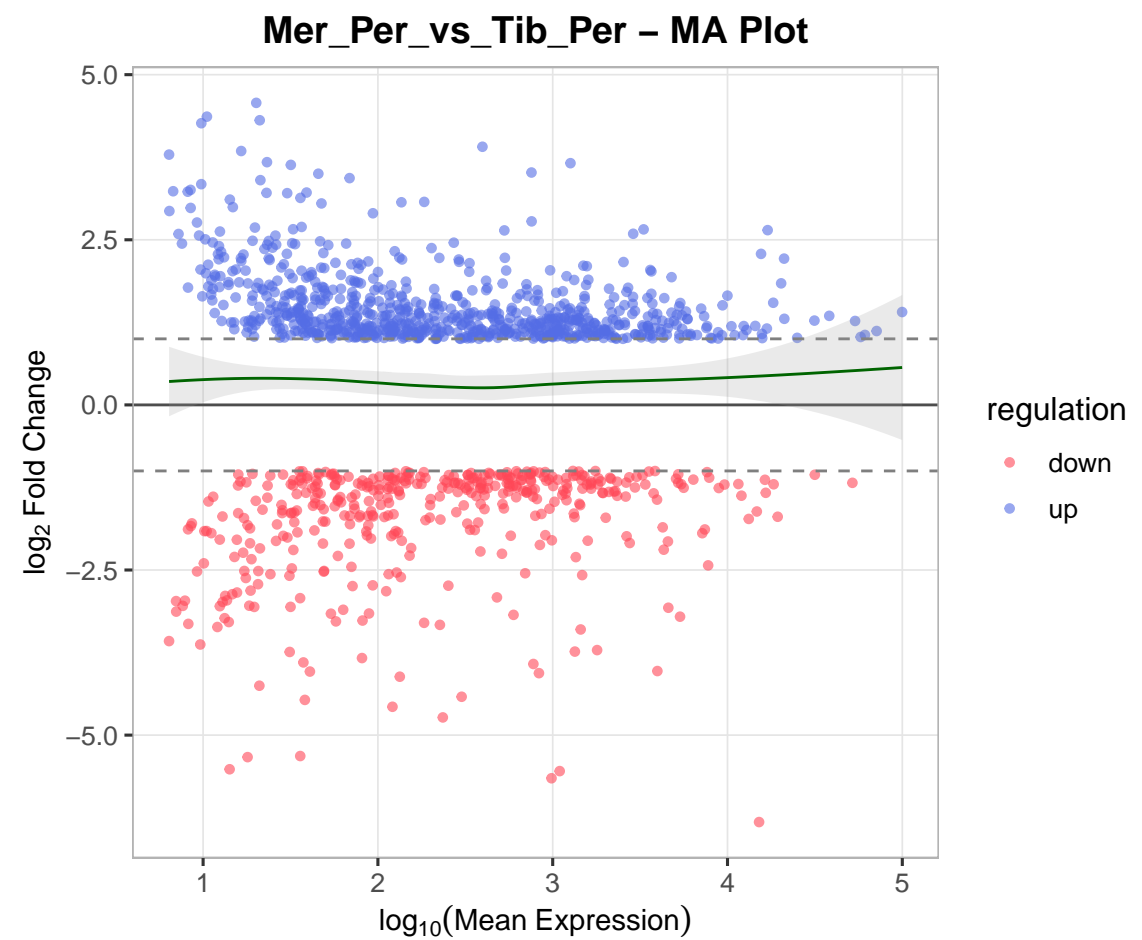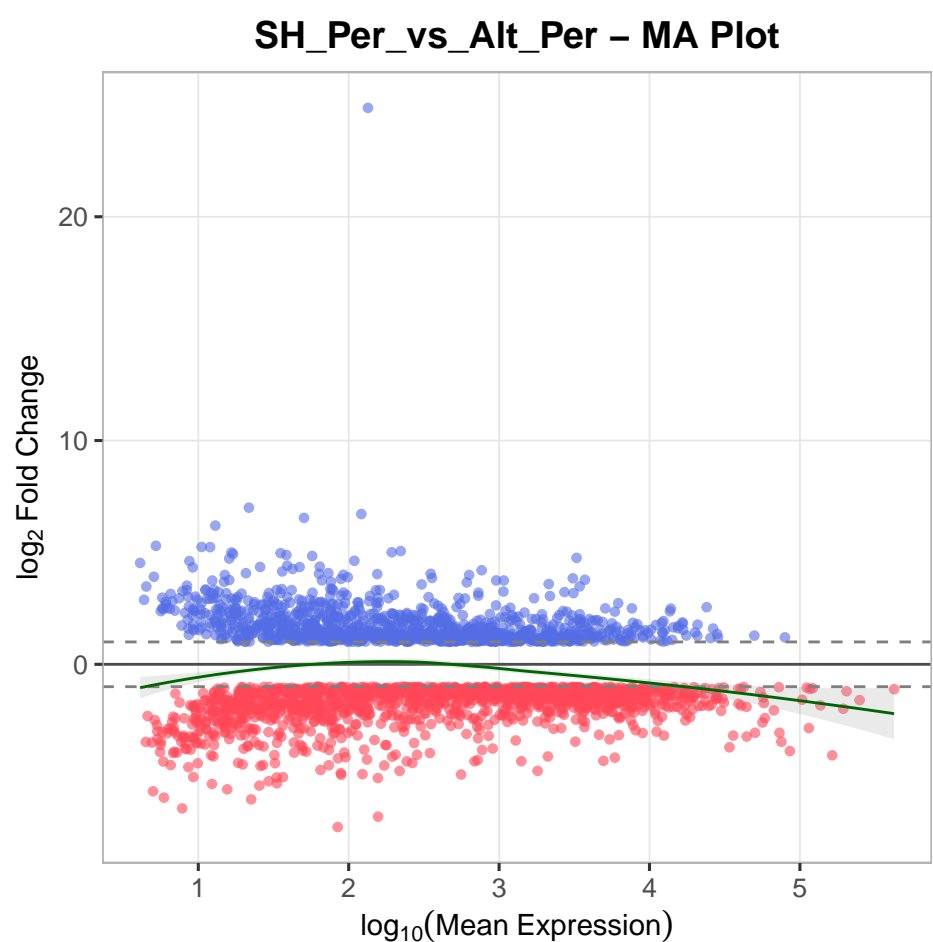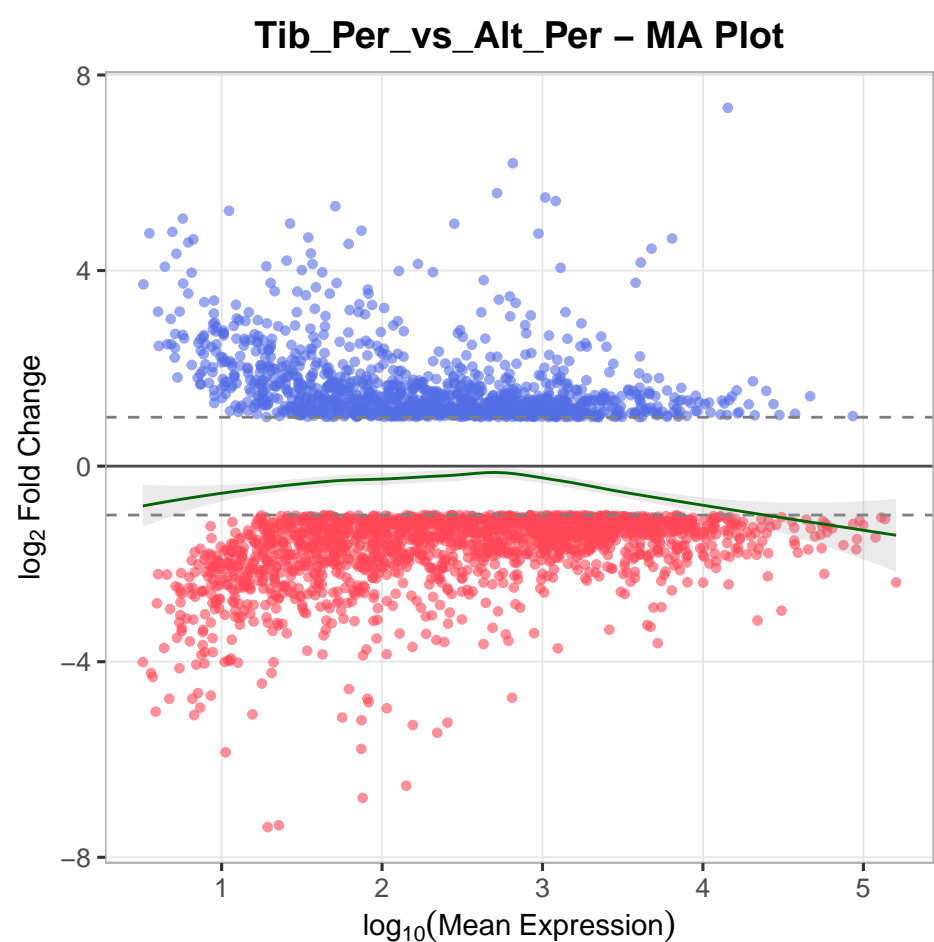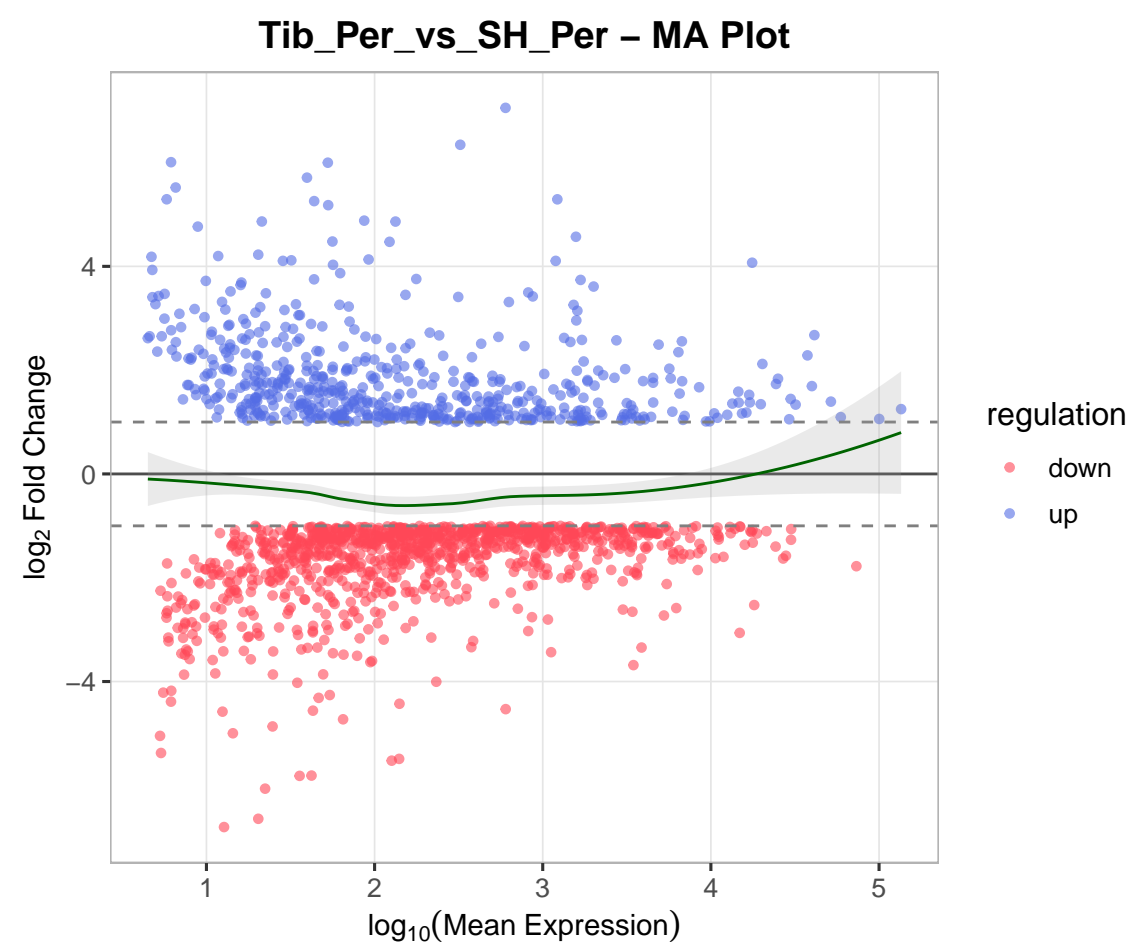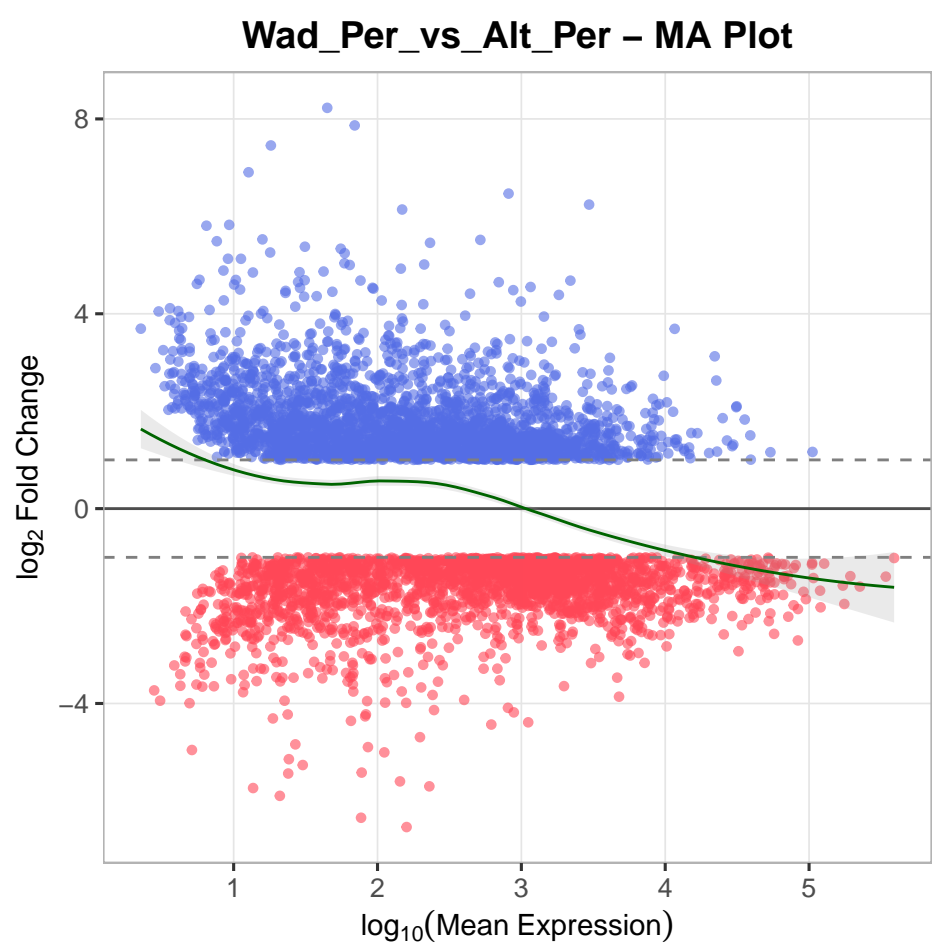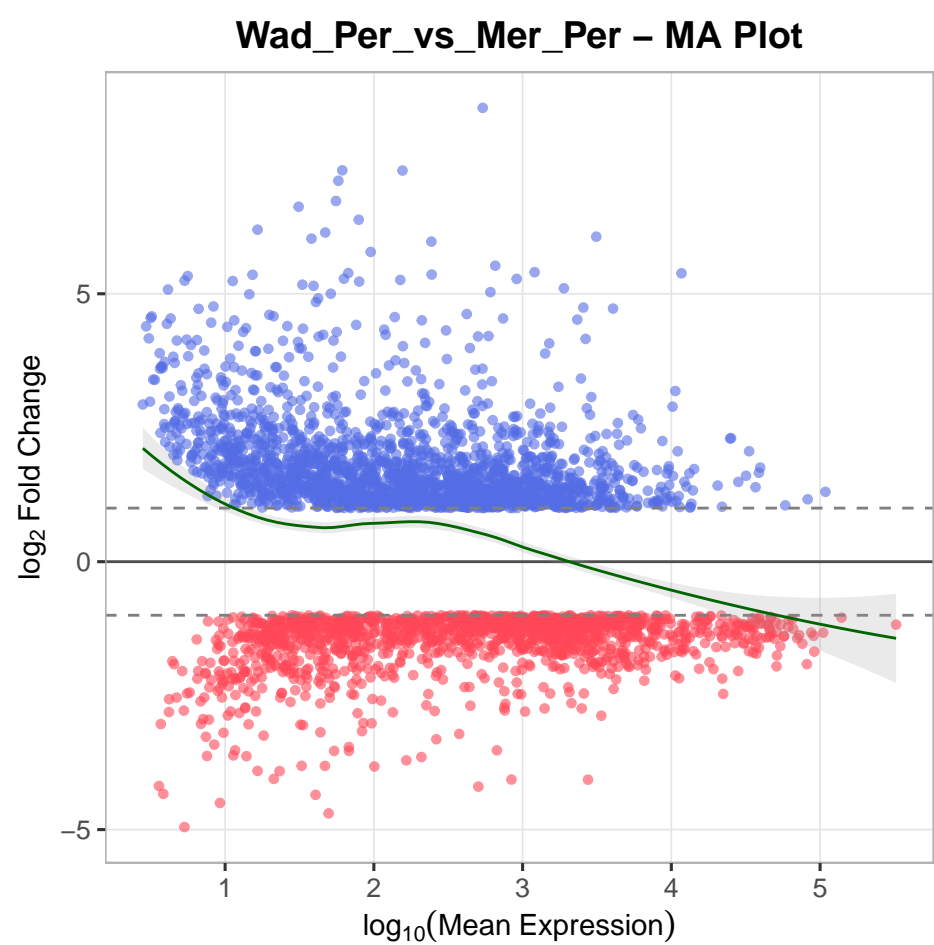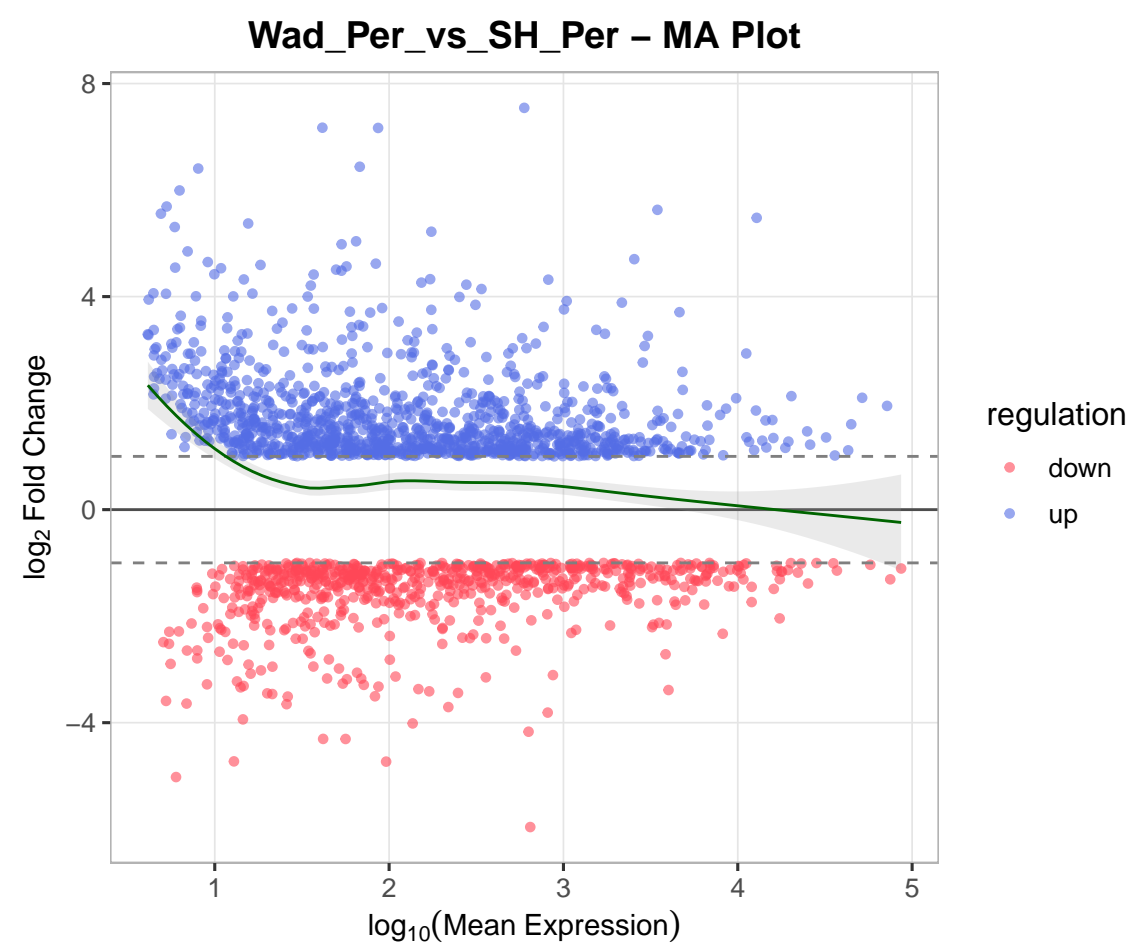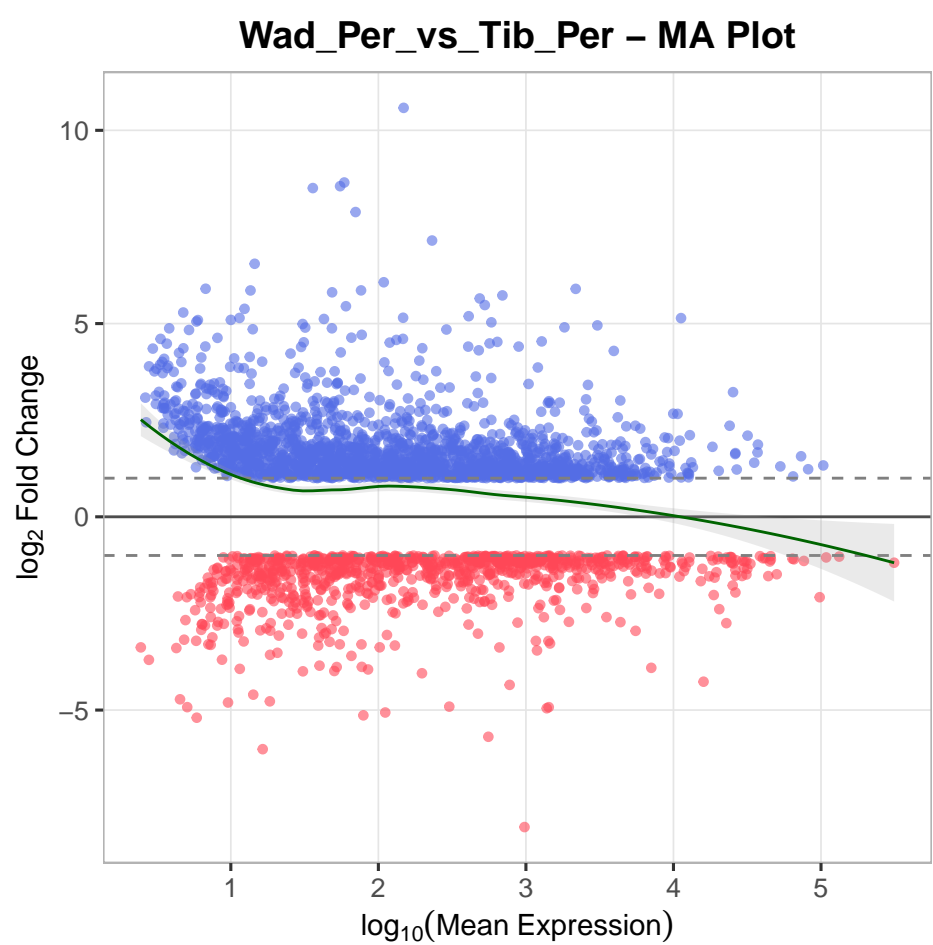

Supplement: Supplementary file 1 [file genes-17-00093-s001.zip › Supplementary Figures/Supplementary Figure 8.pdf]

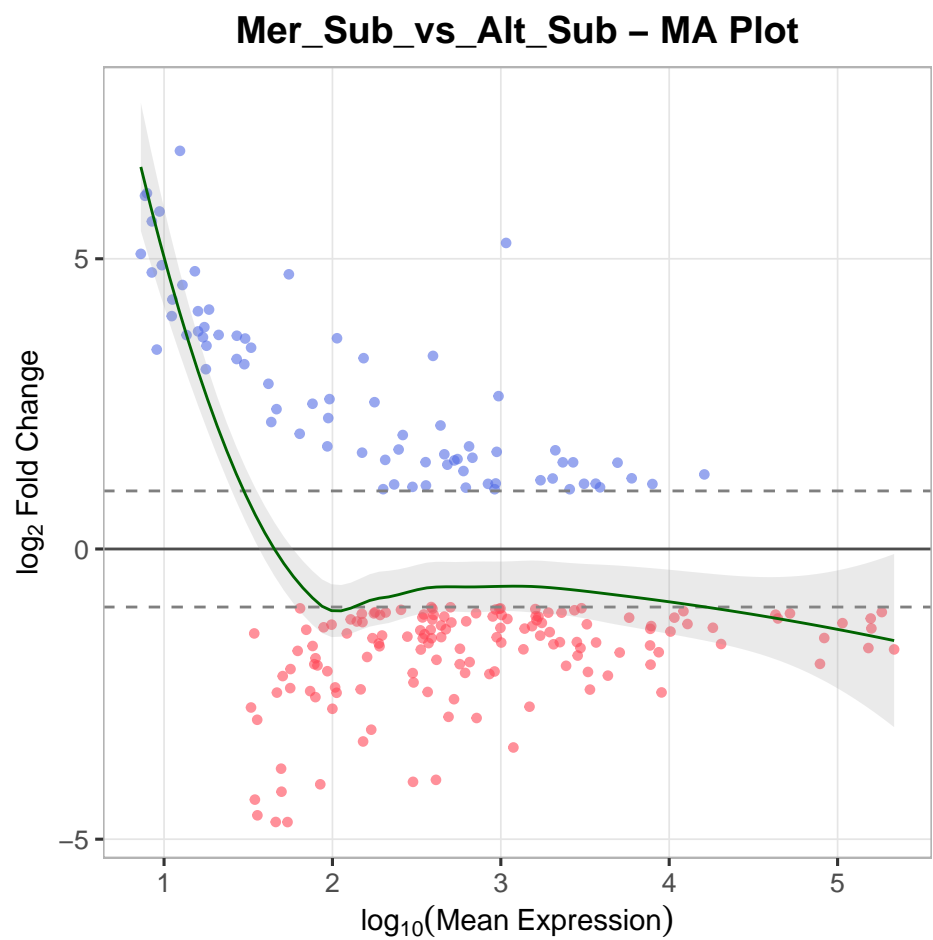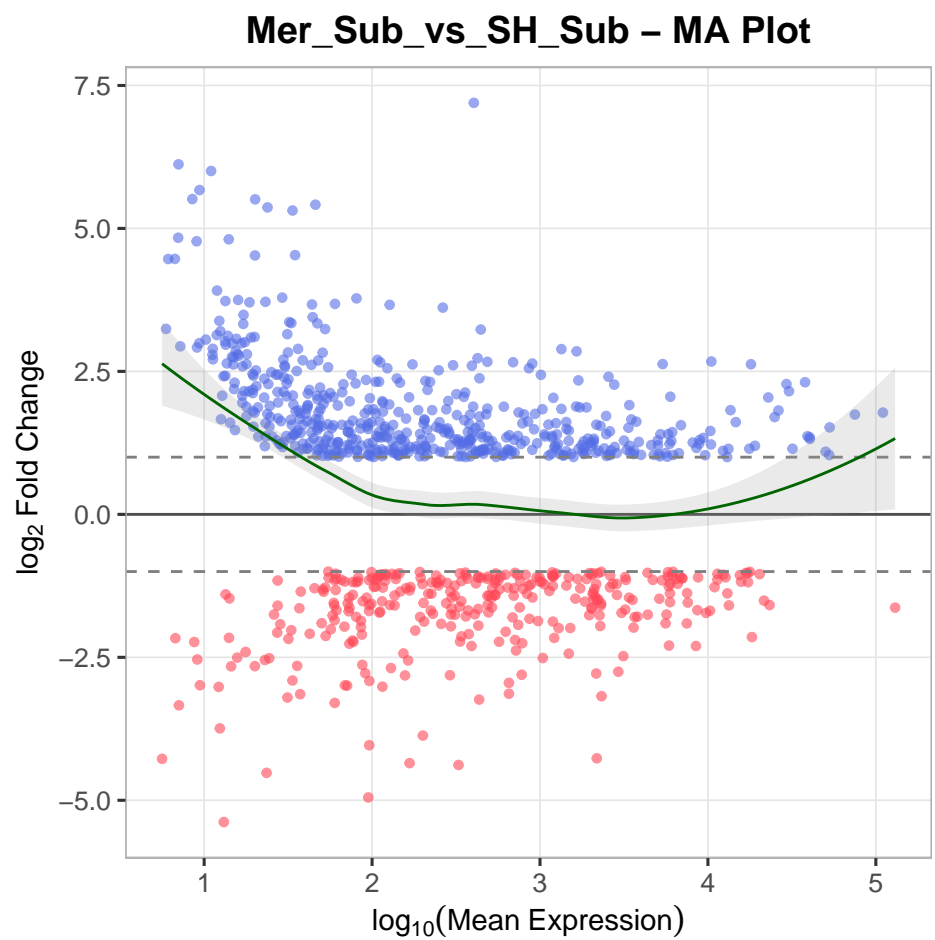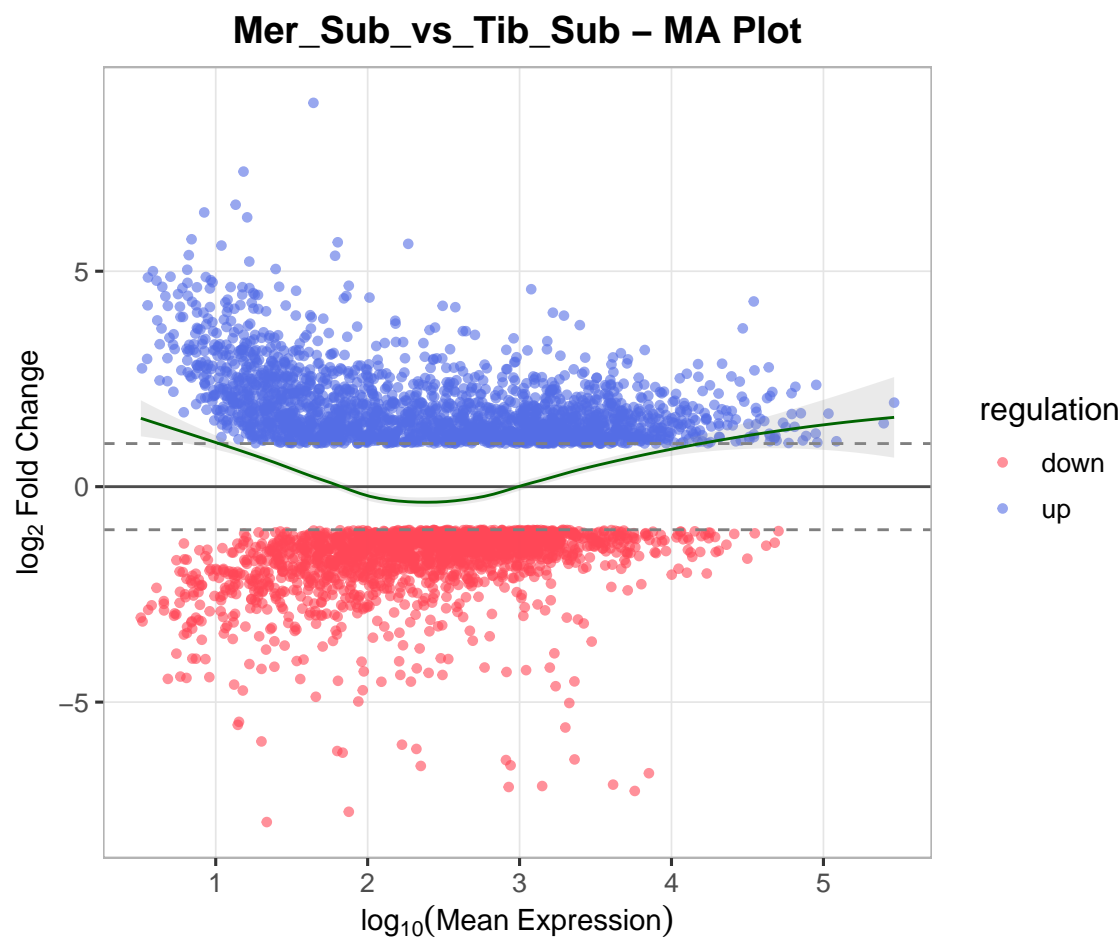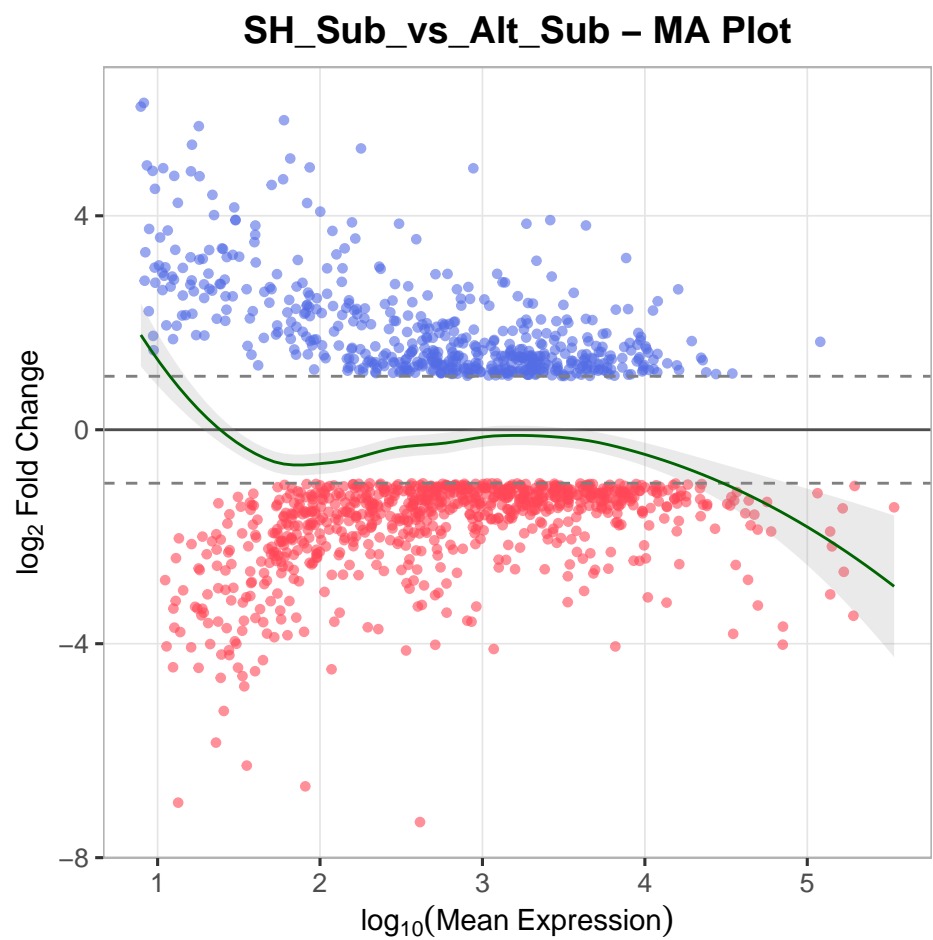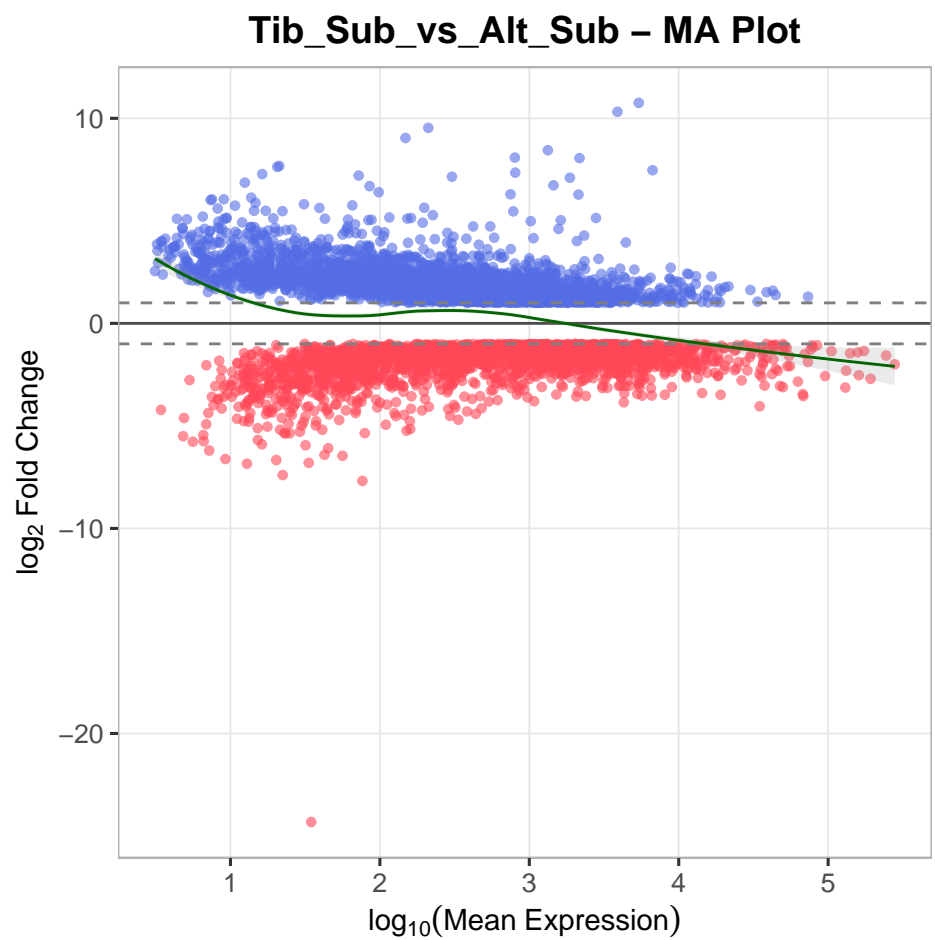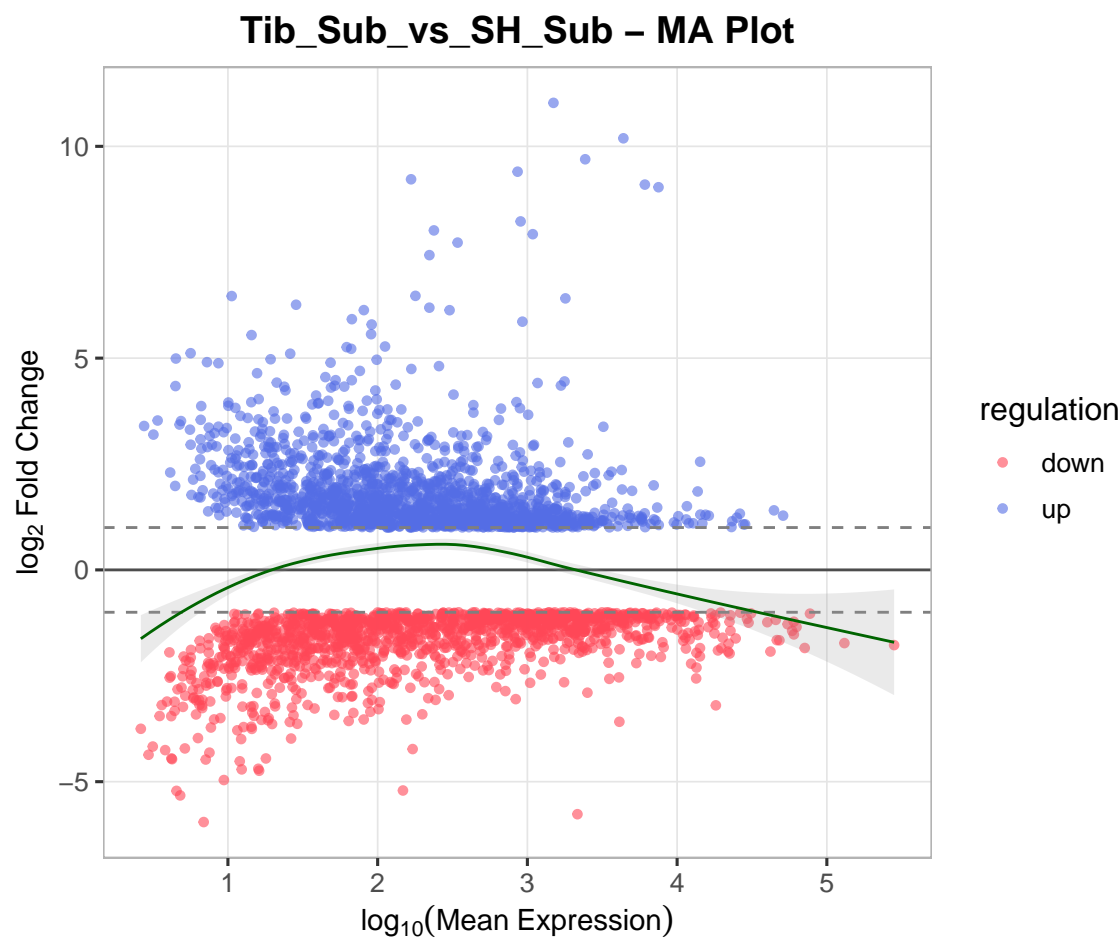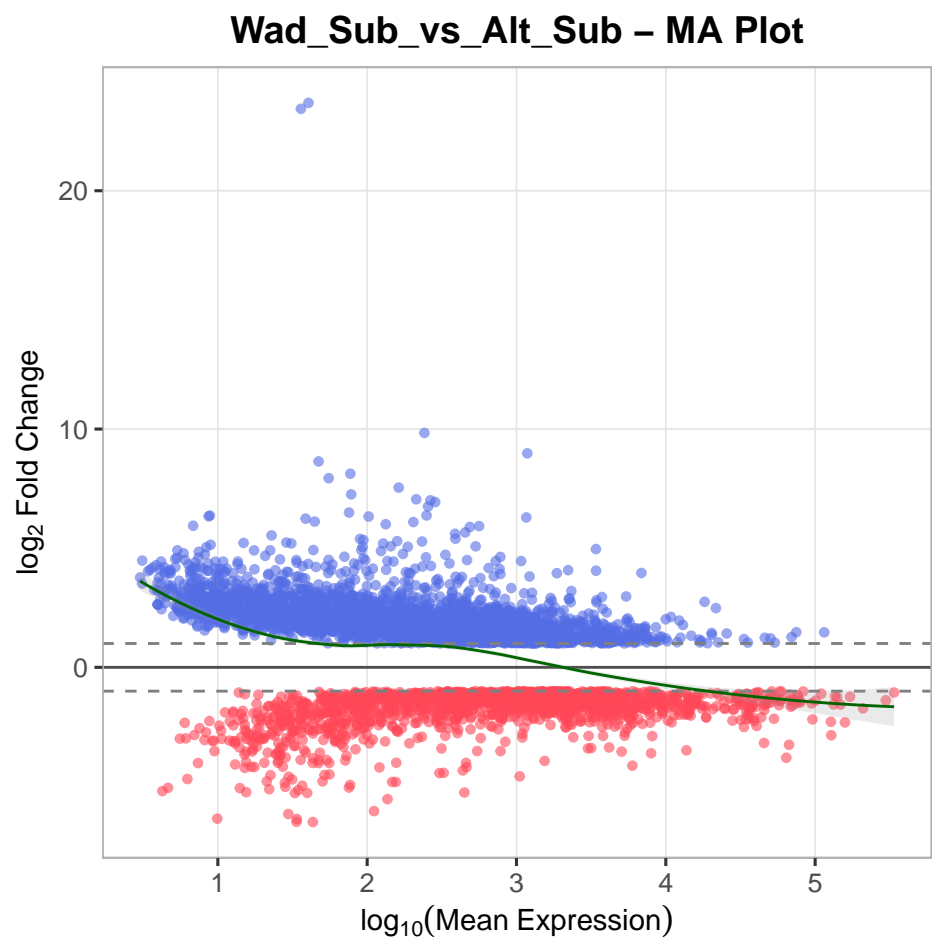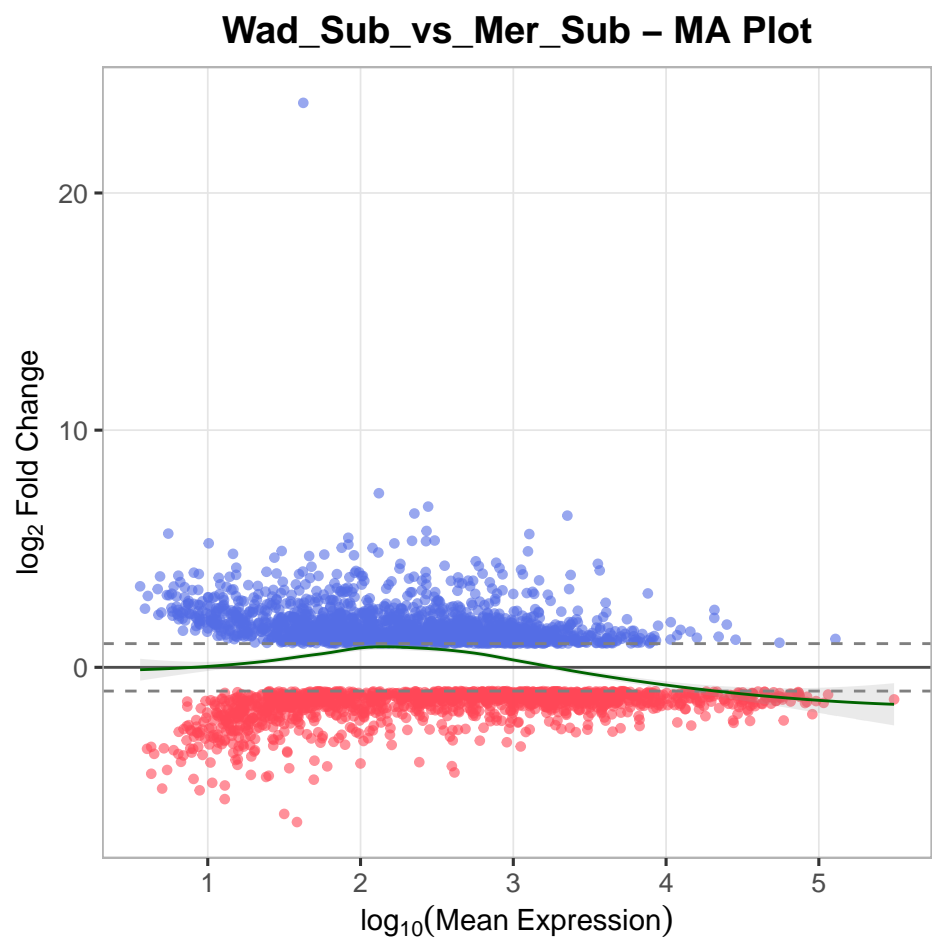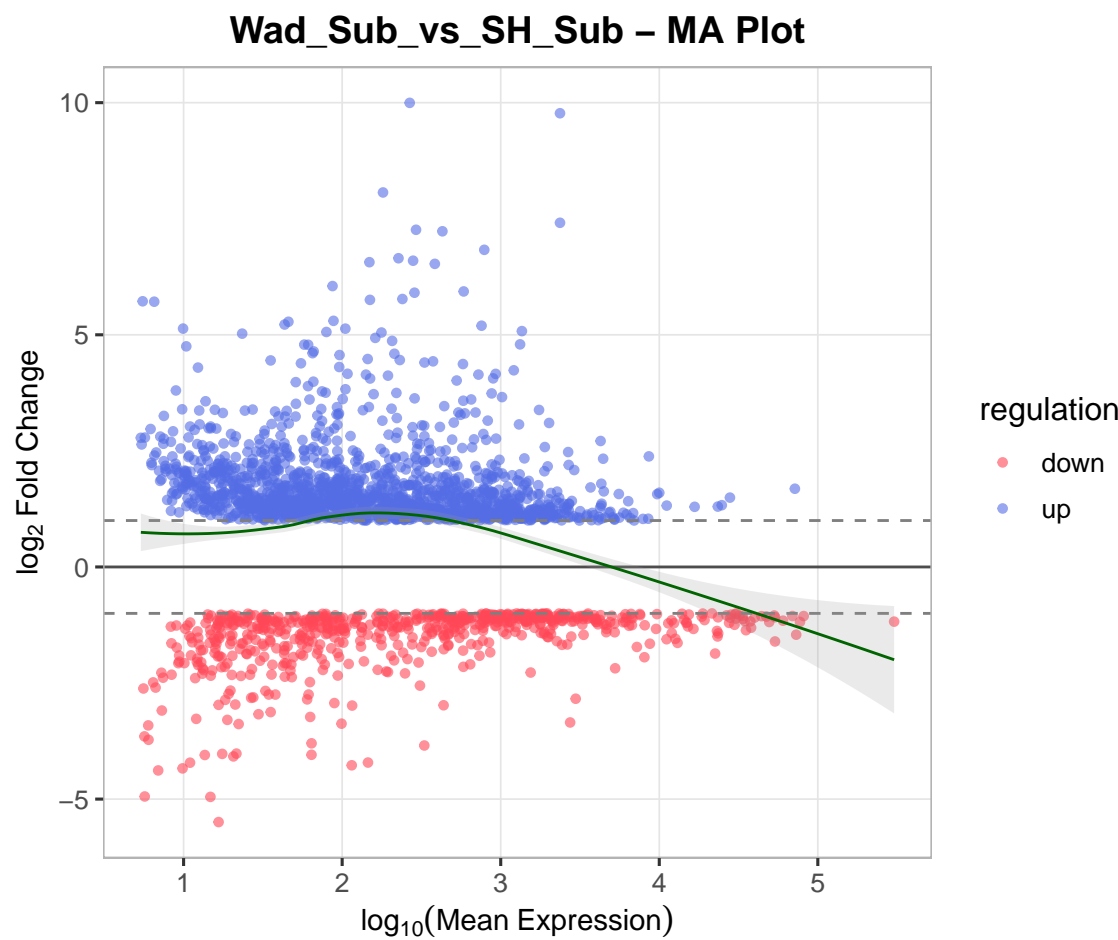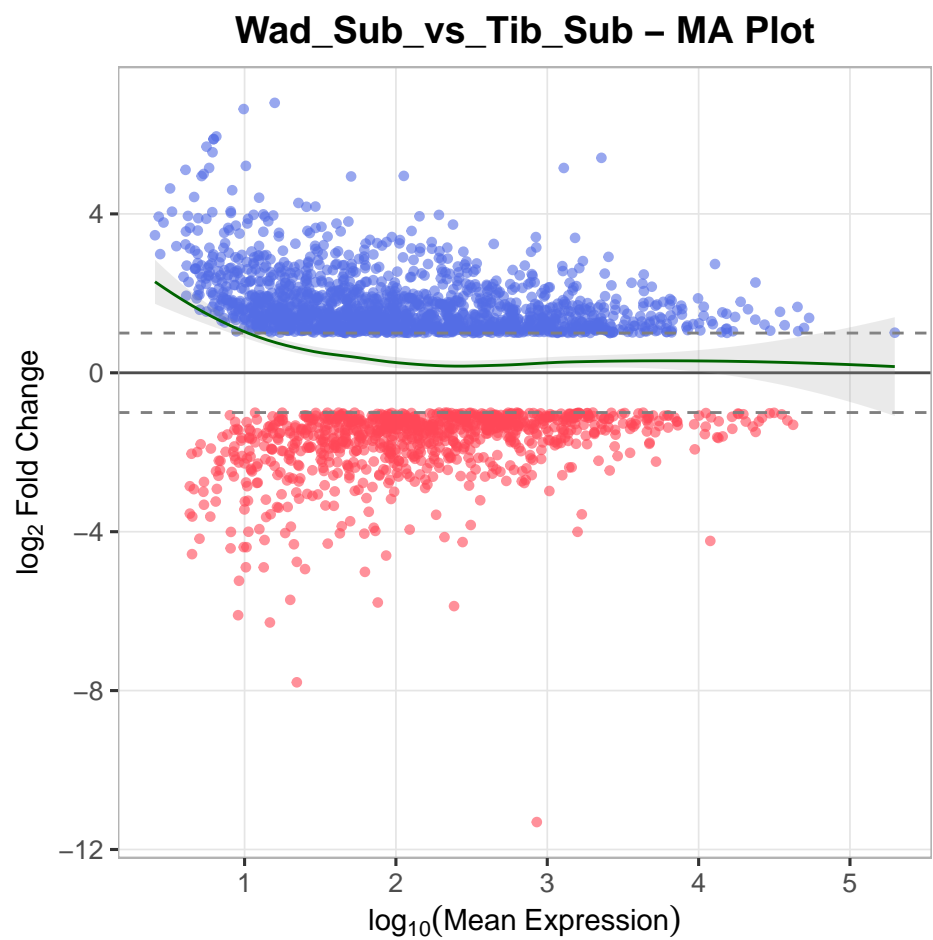

Supplement: Supplementary file 1 [file genes-17-00093-s001.zip › Supplementary Figures/Supplementary Figure 9.pdf]
